# Supplementary material for: Availability of evidence and comparative effectiveness for surgical versus drug interventions: an overview of systematic reviews and meta-analyses
Source: BMJ Open. 2024 Jan 9;14(1):e076675. doi: 10.1136/bmjopen-2023-076675 (PMC10810041; doi:10.1136/bmjopen-2023-076675)
Supplement: Supplementary data [file bmjopen-2023-076675supp001.pdf]

Availability of evidence and comparative effectiveness for surgical versus drug interventions: an overview of systematic reviews

Emmanuel A. Zavalis[1,2*], Anaïs Rameau[3*], Anirudh Saraswathula[4*], Joachim Vist[1], Ewoud Schuit[5,6],

John P. A. Ioannidis[2,7]

*co-first authors

1 Department of Learning Informatics Management and Ethics, Karolinska Institutet, Stockholm, Sweden

2 Meta-Research Innovation Center at Stanford (METRICS), Stanford University, Stanford, CA, USA

3 Sean Parker Institute for the Voice, Department of Otolaryngology–Head and Neck Surgery, Weill Cornell Medical College, New York, NY, USA

4 Department of Otolaryngology–Head and Neck Surgery, Johns Hopkins University School of Medicine, Baltimore, MD, USA

5 Julius Center, University Medical Center Utrecht, Utrecht University, Utrecht, the Netherlands

6 Cochrane Netherland, University Medical Center Utrecht, Utrecht University, Utrecht, the Netherlands

7 Stanford Prevention Research Center, Department of Medicine, and Department of Epidemiology and Population Health, Stanford University School of Medicine, Stanford, CA, USA

Supplementary Materials - Index

Supplementary Data

Supplement 1 – List of included studies *pag. 2*

Supplementary Figures and Tables

Supplementary Table 1 *pag. 7*

Supplementary Table 2 *pag. 8*

Zavalis EA, *et al. BMJ Open* 2024; 14:e076675. doi: 10.1136/bmjopen-2023-076675

Supplementary Data

Supplement 1 – List of included studies

CDSR_ID	Title	Specialty	Comparison available
CD005624.PUB4	Interventions for great saphenous vein incompetence	vascular surgery	No
CD006931.PUB2	Submacular surgery for choroidal neovascularisation secondary to age-related macular degeneration	ophthalmology	No
CD002764.PUB2	Surgery for the resolution of symptoms in malignant bowel obstruction in advanced gynaecological and gastrointestinal cancer	general surgery	No
CD007119.PUB2	Interventions for restoring patency of occluded central venous catheter lumens	vascular surgery	No
CD008509.PUB3	Alpha-blockers as medical expulsive therapy for ureteral stones	urology	No
CD013085.PUB2	Balneotherapy for chronic venous insufficiency	vascular surgery	No
CD009959.PUB2	Interventions for the treatment of Frey's syndrome	otolaryngology	No
CD004008.PUB3	Interventions for trachoma trichiasis	ophthalmology	No
CD006134.PUB5	Oral contraceptives for functional ovarian cysts	obstetrics and gynecology	No
CD011650.PUB2	Management of people with early- or very early-stage hepatocellular carcinoma	general surgery	No
CD001081.PUB4	Carotid endarterectomy for symptomatic carotid stenosis	vascular surgery	Yes
CD010244.PUB2	Resection versus other treatments for locally advanced pancreatic cancer	general surgery	Yes
CD012432.PUB2	Interventions for managing medication-related osteonecrosis of the jaw	otolaryngology	No
CD010260.PUB2	Hysterectomy with radiotherapy or chemotherapy or both for women with locally advanced cervical cancer	obstetrics and gynecology	No
CD012602.PUB2	Methods for managing miscarriage: a network meta-analysis	obstetrics and gynecology	Yes
CD006983.PUB3	Decompressive surgery for treating nerve damage in leprosy	neurosurgery	Yes
CD009590.PUB2	Endometriosis: an overview of Cochrane Reviews	obstetrics and gynecology	No
CD005320.PUB2	Operative and non-operative treatment options for dislocation of the hip following total hip arthroplasty	orthopaedic surgery	No
CD010349.PUB2	Iodine-131-meta-iodobenzylguanidine therapy for patients with newly diagnosed high-risk neuroblastoma	neurosurgery	No
CD010712	Nonoperative treatment for lumbar spinal stenosis with neurogenic claudication	orthopaedic surgery	No
CD011478.PUB2	Type II or type III radical hysterectomy compared to chemoradiotherapy as a primary intervention for stage IB2 cervical cancer	obstetrics and gynecology	No
CD002116.PUB2	Drug treatment for faecal incontinence in adults	general surgery	No
CD005029.PUB2	Treatment for ataxia in multiple sclerosis	neurosurgery	No
CD008107.PUB2	Perioperative chemo(radio)therapy versus primary surgery for resectable adenocarcinoma of the stomach, gastroesophageal junction, and lower esophagus	general surgery	No
CD008602.PUB4	Interventions for congenital talipes equinovarus (clubfoot)	orthopaedic surgery	No
CD004461.PUB3	Interventions for recurrent idiopathic epistaxis (nosebleeds) in children	otolaryngology	No
CD006476.PUB3	Management for intussusception in children	general surgery	No
CD009166.PUB2	Cervical stitch (cerclage) for preventing preterm birth in multiple pregnancy	obstetrics and gynecology	No
CD002221.PUB2	Interventions for involutional lower lid entropion	ophthalmology	No
CD009379.PUB2	Amniotic membrane transplantation for acute ocular burns	ophthalmology	Yes
CD003296.PUB3	Retinoids for preventing the progression of cervical intra-epithelial neoplasia	obstetrics and gynecology	No
CD004917.PUB3	Interventions for infantile esotropia	ophthalmology	No
CD003431.PUB3	Non surgical therapy for anal fissure	general surgery	Yes
CD007340.PUB2	Bariatric surgery for non-alcoholic steatohepatitis in obese patients	general surgery	No

Zavalis EA, *et al. BMJ Open* 2024; 14:e076675. doi: 10.1136/bmjopen-2023-076675

CDSR_ID	Title	Specialty	Comparison available
CD001122.PUB5	Laparoscopic ovarian drilling for ovulation induction in women with anovulatory polycystic ovary syndrome	obstetrics and gynecology	Yes
CD007156.PUB2	Interventions for the management of oral submucous fibrosis	otolaryngology	No
CD012802.PUB2	Ab interno supraciliary microstent surgery for open-angle glaucoma	ophthalmology	No
CD004399.PUB3	Medical versus surgical interventions for open angle glaucoma	ophthalmology	No
CD009266.PUB2	Non-steroidal antiandrogen monotherapy compared with luteinising hormone-releasing hormone agonists or surgical castration monotherapy for advanced prostate cancer	urology	No
CD010273.PUB2	Interventions for treating postpartum constipation	general surgery	No
CD009366.PUB2	Lumbar sympathectomy versus prostanoids for critical limb ischaemia due to non-reconstructable peripheral arterial disease	orthopaedic surgery	Yes
CD007060.PUB2	Liver resection versus other treatments for neuroendocrine tumours in patients with resectable liver metastases	general surgery	No
CD008088.PUB3	Anti-TNF-Œ± treatment for pelvic pain associated with endometriosis	obstetrics and gynecology	No
CD004982.PUB6	Treatment for superficial thrombophlebitis of the leg	vascular surgery	Yes
CD007939.PUB2	Single herbal medicine for diabetic retinopathy	ophthalmology	No
CD002000.PUB3	Bypass surgery for chronic lower limb ischaemia	vascular surgery	No
CD012017.PUB2	Grommets (ventilation tubes) for recurrent acute otitis media in children	otolaryngology	Yes
CD009968.PUB2	Botulinum toxin for upper oesophageal sphincter dysfunction in neurological swallowing disorders	general surgery	No
CD004272.PUB3	Surgery versus primary endocrine therapy for operable primary breast cancer in elderly women (70 years plus)	general surgery	Yes
CD007118.PUB2	Palliative cytoreductive surgery versus other palliative treatments in patients with unresectable liver metastases from gastro-entero-pancreatic neuroendocrine tumours	general surgery	No
CD006714.PUB2	Surgical versus medical methods for second trimester induced abortion	obstetrics and gynecology	Yes
CD011174.PUB2	Interventions for non-tubal ectopic pregnancy	obstetrics and gynecology	No
CD010541.PUB3	Surgery for epilepsy	neurosurgery	Yes
CD013034.PUB2	Surgery for patellar tendinopathy (jumper's knee)	orthopaedic surgery	Yes
CD007481.PUB3	Chemical pleurodesis versus surgical intervention for persistent and recurrent pneumothoraces in cystic fibrosis	thoracic surgery	No
CD003712.PUB3	Transmyocardial laser revascularization versus medical therapy for refractory angina	cardiac surgery	Yes
CD008997.PUB2	Non-resection versus resection for an asymptomatic primary tumour in patients with unresectable Stage IV colorectal cancer	general surgery	No
CD005081.PUB3	Medical and surgical treatment for ocular myasthenia	ophthalmology	No
CD013099.PUB2	Interventions for bacterial folliculitis and boils (furuncles and carbuncles)	general surgery	No
CD011837.PUB2	Medical and surgical interventions for the treatment of usual-type vulval intraepithelial neoplasia	obstetrics and gynecology	No
CD003951.PUB3	Surgical versus medical treatment with cyclooxygenase inhibitors for symptomatic patent ductus arteriosus in preterm infants	cardiac surgery	Yes
CD007261.PUB2	Interventions for managing temporomandibular joint osteoarthritis	orthopaedic surgery	No
CD003193.PUB4	Anticholinergic drugs versus non-drug active therapies for non-neurogenic overactive bladder syndrome in adults	urology	No
CD009493.PUB2	N-acetylcarnosine (NAC) drops for age-related cataract	ophthalmology	No
CD005198.PUB3	Therapeutic interventions for Burkitt lymphoma in children	otolaryngology	No
CD004981.PUB4	Treatment for femoral pseudoaneurysms	vascular surgery	No
CD003525.PUB2	Surgery for lateral elbow pain	orthopaedic surgery	No
CD013006.PUB2	Interventions for the management of obesity in people with bipolar disorder	general surgery	No
CD013404.PUB2	Surgical interventions for treating intracapsular hip fractures in older adults: a network meta-analysis	orthopaedic surgery	No
CD011725.PUB2	Indomethacin for intracranial hypertension secondary to severe traumatic brain injury in adults	neurosurgery	No

CDSR_ID	Title	Specialty	Comparison available
CD009526.PUB2	Ovarian surgery for symptom relief in women with polycystic ovary syndrome	obstetrics and gynecology	Yes
CD003855.PUB3	Surgery versus medical therapy for heavy menstrual bleeding	obstetrics and gynecology	Yes
CD009505.PUB2	Aromatase inhibitors for uterine fibroids	obstetrics and gynecology	No
CD003037.PUB2	Medical versus surgical methods for first trimester termination of pregnancy	obstetrics and gynecology	Yes
CD011169.PUB2	Selective oestrogen receptor modulators (SERMs) for endometriosis	obstetrics and gynecology	No
CD007924.PUB3	Medical interventions for high-grade vulval intraepithelial neoplasia	obstetrics and gynecology	No
CD008111.PUB2	Thymectomy for non-thymomatous myasthenia gravis	thoracic surgery	No
CD007223.PUB4	Medical treatments for incomplete miscarriage	obstetrics and gynecology	Yes
CD010308.PUB2	Interventions for melanoma in situ, including lentigo maligna	general surgery	No
CD007468.PUB4	Surgical interventions for the early management of Bell's palsy	neurosurgery	No
CD007792.PUB2	Palliative surgery versus medical management for bowel obstruction in ovarian cancer	general surgery	No
CD008455.PUB2	Interventions for treating bisphosphonate-related osteonecrosis of the jaw (BRONJ)	orthopaedic surgery	No
CD002115.PUB5	Management of faecal incontinence and constipation in adults with central neurological diseases	general surgery	No
CD006991.PUB2	Surgical versus medical interventions for chronic rhinosinusitis with nasal polyps	otolaryngology	No
CD001496.PUB2	Pharmacological and surgical interventions for the treatment of gastro-oesophageal reflux in adults and children with asthma	general surgery	No
CD008571.PUB2	Interventions for women with endometrioma prior to assisted reproductive technology	obstetrics and gynecology	No
CD006544.PUB3	Prostanoids for critical limb ischaemia	vascular surgery	No
CD003435.PUB2	Surgical decompression for cerebral oedema in acute ischaemic stroke	neurosurgery	Yes
CD013325.PUB2	Interventions for treating people with symptoms of bladder pain syndrome: a network meta-analysis	urology	No
CD001066.PUB3	Interventions for varicose veins and leg oedema in pregnancy	vascular surgery	No
CD006388.PUB2	Octreotide for the treatment of chylothorax in neonates	thoracic surgery	No
CD003658.PUB3	Needling for encapsulated trabeculectomy filtering blebs	ophthalmology	No
CD006152.PUB2	Decompressive surgery of lower limbs for symmetrical diabetic peripheral neuropathy	orthopaedic surgery	No
CD001896.PUB2	Surgical interruption of pelvic nerve pathways for primary and secondary dysmenorrhoea	obstetrics and gynecology	No
CD004699.PUB2	Surgery for local and locally advanced non-small cell lung cancer	thoracic surgery	No
CD002867	Treatments for secondary postpartum haemorrhage	obstetrics and gynecology	No
CD006373.PUB2	Interventions for treating functional dysphonia in adults	otolaryngology	No
CD001541.PUB3	Interventions for ingrowing toenails	general surgery	No
CD013469.PUB2	Surgical and medical interventions for abdominal aortic graft infections	vascular surgery	No
CD001219	Corticosteroids for the resolution of malignant bowel obstruction in advanced gynaecological and gastrointestinal cancer	general surgery	No
CD005304.PUB3	Interventions for primary (intrinsic) tracheomalacia in children	thoracic surgery	No
CD011498.PUB2	Non-surgical versus surgical treatment for oesophageal cancer	general surgery	Yes
CD002784.PUB3	Surgery versus thrombolysis for initial management of acute limb ischaemia	vascular surgery	Yes
CD006499.PUB4	Botulinum toxin for the treatment of strabismus	ophthalmology	Yes
CD005024.PUB3	Surgery for traumatic optic neuropathy	general surgery	No
CD003243.PUB3	Laparoscopic fundoplication surgery versus medical management for gastro-oesophageal reflux disease (GORD) in adults	general surgery	Yes
CD003118.PUB2	Interventions for the treatment of Morton's neuroma	orthopaedic surgery	No
CD001001.PUB3	Lung volume reduction surgery for diffuse emphysema	thoracic surgery	No
CD010784.PUB3	Medical and surgical interventions for the treatment of urinary stones in children	urology	No

Zavalis EA, *et al. BMJ Open* 2024; 14:e076675. doi: 10.1136/bmjopen-2023-076675

CDSR_ID	Title	Specialty	Comparison available
CD000324.PUB2	Interventions for tubal ectopic pregnancy	obstetrics and gynecology	No
CD000526.PUB2	Interventions for treating tuberculous pericarditis	cardiac surgery	No
CD004156.PUB4	Treatment for spasticity in amyotrophic lateral sclerosis/motor neuron disease	neurosurgery	No
CD004159.PUB3	Treatment for meralgia paraesthetica	neurosurgery	No
CD006797.PUB2	Surgical resection versus non-surgical treatment for hepatic node positive patients with colorectal liver metastases	general surgery	No
CD007510.PUB3	Botulinum toxin for masseter hypertrophy	otolaryngology	No
CD011523.PUB2	Medical versus surgical treatment for refractory or recurrent peptic ulcer	general surgery	No
CD001802.PUB3	Tonsillectomy or adenotonsillectomy versus non-surgical treatment for chronic/recurrent acute tonsillitis	otolaryngology	Yes
CD007383.PUB3	Surgical versus non-surgical management of abdominal injury	general surgery	No
CD006981.PUB2	Treatment for sialorrhea (excessive saliva) in people with motor neuron disease/amyotrophic lateral sclerosis	otolaryngology	No
CD001829.PUB4	Interventions for treating oral leukoplakia to prevent oral cancer	otolaryngology	No
CD001934.PUB2	Surgical versus non-surgical interventions for vocal cord nodules	otolaryngology	No
CD003412.PUB3	Interventions for basal cell carcinoma of the skin	dermatology	Yes
CD003425.PUB4	Splenectomy versus conservative management for acute sequestration crises in people with sickle cell disease	general surgery	No
CD003983.PUB3	Decompressive craniectomy for the treatment of high intracranial pressure in closed traumatic brain injury	neurosurgery	Yes
CD004098.PUB2	Levothyroxine or minimally invasive therapies for benign thyroid nodules	general surgery	No
CD004437.PUB6	Thrombolytic therapy for pulmonary embolism	cardiac surgery	No
CD004927.PUB4	Surgical management of functional bladder outlet obstruction in adults with neurogenic bladder dysfunction	urology	No
CD005619.PUB3	Subacromial decompression surgery for rotator cuff disease	orthopaedic surgery	No
CD006032.PUB4	Steroids for traumatic optic neuropathy	ophthalmology	No
CD006746.PUB4	Laser peripheral iridoplasty for chronic angle closure	ophthalmology	No
CD007281.PUB2	Interventions for cutaneous Bowen's disease	dermatology	No
CD007404.PUB2	Interventions for central giant cell granuloma (CGCG) of the jaws	otolaryngology	No
CD007535.PUB4	Chinese herbal medicine for subfertile women with polycystic ovarian syndrome	obstetrics and gynecology	No
CD008280.PUB2	Interventions for atrophic rhinitis	otolaryngology	No
CD009244.PUB2	Interventions for anal canal intraepithelial neoplasia	general surgery	No
CD010287.PUB3	Aromatase inhibitors (letrozole) for subfertile women with polycystic ovary syndrome	obstetrics and gynecology	Yes
CD010651.PUB2	Surgical versus non-surgical management for pleural empyema	thoracic surgery	Yes
CD011160.PUB2	Anti-vascular endothelial growth factor for choroidal neovascularisation in people with pathological myopia	ophthalmology	Yes
CD012742.PUB2	Subconjunctival draining minimally-invasive glaucoma devices for medically uncontrolled glaucoma	ophthalmology	No
CD012743.PUB2	Ab interno trabecular bypass surgery with iStent for open-angle glaucoma	ophthalmology	Yes
CD012834.PUB2	Medical and surgical abortion for women living with HIV	obstetrics and gynecology	No
CD012879.PUB2	Shoulder replacement surgery for osteoarthritis and rotator cuff tear arthropathy	orthopaedic surgery	No
CD006131.PUB3	Interventions for Mooren's ulcer	dermatology	No
CD007677.PUB4	Pentoxifylline for the treatment of endometriosis-associated pain and infertility	obstetrics and gynecology	No
CD012740.PUB2	Ab interno trabecular bypass surgery with Schlemm´s canal microstent (Hydrus) for open angle glaucoma	ophthalmology	No
CD006151.PUB3	Fundoplication versus postoperative medication for gastro-oesophageal reflux in children with neurological impairment undergoing gastrostomy	general surgery	No
CD010081.PUB2	Interventions for hidradenitis suppurativa	dermatology	No
CD007630.PUB2	Surgical orbital decompression for thyroid eye disease	otolaryngology	Yes
CD011165.PUB2	Tonsillectomy or adenotonsillectomy versus non-surgical management for obstructive sleep-disordered breathing in children	otolaryngology	No

CDSR_ID	Title	Specialty	Comparison available
CD005656.PUB3	Intravitreal steroids for macular edema in diabetes	ophthalmology	No
CD009860.PUB2	Surgery for trigger finger	orthopaedic surgery	Yes
CD013502	Surgery for rotator cuff tears	orthopaedic surgery	Yes
CD002180	Surgery versus non-surgical treatment for bronchiectasis	thoracic surgery	No
CD010868.PUB2	Interventions for dissociated vertical deviation	ophthalmology	No
CD001408.PUB2	Botulinum toxin type A in the treatment of lower limb spasticity in children with cerebral palsy	orthopaedic surgery	No
CD003919.PUB2	Laser trabeculoplasty for open angle glaucoma	ophthalmology	Yes
CD010312.PUB2	Prostaglandins for management of retained placenta	obstetrics and gynecology	No
CD011693.PUB3	Ab interno trabecular bypass surgery with Trabectome for open-angle glaucoma	ophthalmology	No
CD008669.PUB3	Tonsillectomy for periodic fever, aphthous stomatitis, pharyngitis and cervical adenitis syndrome (PFAPA)	otolaryngology	No
CD008128.PUB2	Treatment of valvular heart disease during pregnancy for improving maternal and neonatal outcome	cardiac surgery	No
CD001923.PUB2	Carotid endarterectomy for asymptomatic carotid stenosis	vascular surgery	Yes
CD010960.PUB2	Injection therapies for Achilles tendinopathy	orthopaedic surgery	No
CD003738.PUB3	Interventions for preventing posterior capsule opacification	ophthalmology	No
CD013000.PUB2	Interventions for orbital lymphangioma	otolaryngology	No
CD008282	Adenoidectomy for recurrent or chronic nasal symptoms in children	otolaryngology	No
CD003263.PUB5	Interventions for vitiligo	dermatology	No
CD008583.PUB3	Ultrasound-guided transvaginal ovarian needle drilling for clomiphene-resistant polycystic ovarian syndrome in subfertile women	obstetrics and gynecology	No
CD007810.PUB2	Adenoidectomy for otitis media in children	otolaryngology	No
CD006181.PUB2	Prophylactic surgical ligation of patent ductus arteriosus for prevention of mortality and morbidity in extremely low birth weight infants	cardiac surgery	No
CD011917.PUB2	Surgery for limited-stage small-cell lung cancer	thoracic surgery	No
CD010264.PUB2	Surgical versus non-surgical treatment for lumbar spinal stenosis	orthopaedic surgery	Yes
CD008732.PUB2	Macular grid laser photocoagulation for branch retinal vein occlusion	ophthalmology	No
CD011680.PUB2	Interventions for necrotizing soft tissue infections in adults	general surgery	No
CD001801.PUB3	Grommets (ventilation tubes) for hearing loss associated with otitis media with effusion in children	otolaryngology	No
CD006205.PUB4	Interventions for the treatment of oral and oropharyngeal cancers: surgical treatment	otolaryngology	No
CD009245.PUB3	Interventions for the treatment of Paget's disease of the vulva	obstetrics and gynecology	No
CD012798.PUB3	Interventions for treating distal intestinal obstruction syndrome (DIOS) in cystic fibrosis	general surgery	No
CD008089.PUB2	Surgery for shoulder osteoarthritis	orthopaedic surgery	No
CD008497.PUB3	Deep brain and cortical stimulation for epilepsy	neurosurgery	No
CD004325.PUB2	Surgical versus non-surgical treatment for acute anterior shoulder dislocation	orthopaedic surgery	No
CD005048.PUB4	Interventions for dysphagia in oesophageal cancer	general surgery	No
CD000200.PUB2	Surgery for primary supratentorial intracerebral haemorrhage	neurosurgery	Yes
CD011031.PUB3	Laparoscopic surgery for endometriosis	obstetrics and gynecology	No
CD010796.PUB2	Surgery for treating hip impingement (femoroacetabular impingement)	orthopaedic surgery	No
CD006769.PUB2	Interventions for late trabeculectomy bleb leak	ophthalmology	No
CD001532.PUB5	Interventions for primary vesicoureteric reflux	urology	Yes
CD008104.PUB2	Interventions for treating osteochondral defects of the talus in adults	orthopaedic surgery	No
CD001552.PUB2	Surgical versus non-surgical treatment for carpal tunnel syndrome	orthopaedic surgery	Yes

Supplementary Figures and Tables

Supplementary table 1. Reviews per specialty

Specialty	Total reviews	Reviews with at least one comparison (%)
Cardiac surgery	6	2 (33)
Dermatology	5	1 (20)
General surgery	35	5 (14)
Neurosurgery	12	5 (42)
Obstetrics and gynecology	31	8 (26)
Ophthalmology	25	5 (20)
Orthopaedic surgery	23	6 (26)
Otolaryngology	23	3 (13)
Thoracic surgery	9	1 (11)
Urology	7	1 (14)
Vascular surgery	12	4 (33)

Zavalis EA, *et al. BMJ Open* 2024; 14:e076675. doi: 10.1136/bmjopen-2023-076675

Supplementary Table 2. Inconclusive comparisons between surgery and drugs

**Surgical arm**	**Drug arm**	**Disease**	**Outcome**	**Treatment effect (95% CI)**	**GRADE assessment**
Cardiac surgery					
Transmyocardial lazer revascularization	Continued medication	Refractory angina	Overall mortality	OR=1.12 (0.77-1.63)	High
			Postoperative mortality (30 d)	OR=1.19 (0.63-2.24)	High
Surgical closure	IV indomethacin	Patent ductus arteriosus	Death before discharge	RR=0.67 (0.34-1.31)	
Dermatology					
Surgical excision	Imiquimod	BCC	Patient-rated good/excellent cosmetic outcome	RR=1 (0.94-1.06)	Low
General surgery					
Surgery	Tamoxifen	Primary breast cancer	Overall survival	HR=0.98 (0.81-1.2)	Low
Laparoscopic fundoplication	Protein pump inhibitors	GERD	Health-related quality of life (<1 y)	SMD=0.14 (-0.02-0.3)+	Very Low
			Health-related QOL (1-5 y)	SMD=0.03 (-0.19-0.24)+	Very Low
			GORD-specific quality of life (1-5 y)	SMD=0.28 (-0.27-0.84)+	Very Low
Oesophagectomy	Chemoradiotherapy and/or radiotherapy	Oesophageal cancer	Short-term mortality	RR=0.39 (0.11-1.35)	Very Low
			Long-term mortality	RR=1.03 (0.92-1.14)	Low
			Medium-term health-related QOL	MD=-0.95 (-2.1-0.2)	Very Low
Neurosurgery					
Decompressive surgery	Prednisolone	Leprosy	Change in sensory score after one year	MD=0.08 (-2.45-2.61)	Very Low
			Proportion of ulnar nerves with sensory improvement after one year	RR=1.13 (0.71-1.77)	Very Low
			Change in motor score after one year	MD=0.82 (-1.34-2.98)	Very Low
			Proportion of ulnar nerves with motor improvement after one year	RR=0.91 (0.64-1.28)	Very Low
Decompressive craniectomy	Medical treatment (including barbiturates)	High ICP in closed TBI	Neurological unfavourable outcome 6 mo	RR=1 (0.71-1.4)	Low
			Mortality 6 mo	RR=0.66 (0.43-1.01)	Moderate
Obstetrics and gynaecology					
Suction aspiration	Vaginal or oral misoprostol	Abortion	Death or serious complication	RR=1 (0.04-25)	

Surgical arm	Drug arm	Disease	Outcome	Treatment effect (95% CI)	GRADE assessme nt
Suction aspiration	Misoprostol	Abortion	Composite outcome of death or serious complication	RR=1.53 (0.45-5.16)	Very Low
Suction aspiration	Misoprostol and mifepristone	Abortion	Complete miscarriage	RR=1.29 (0.96-1.73)	Very Low
			Composite outcome of death or serious complication	RR=0.14 (0.01-2.74)	Very Low
Suction aspiration	Vaginal suppositories or im inj. of 9-methylene-PGE2	Abortion	Abortion not completeted with intended method	OR=0.62 (0.02-16.6)	
			Ongoing pregnancy	OR=1.82 (0.54-6.25)	
			Pelvic infection	OR=0.46 (0.14-1.56)	
Dilatation and curettage	Misoprostol	Abortion	Composite outcome of death or serious complication	RR=0.79 (0.34-1.85)	Very Low
Laparoscopic ovarian drilling	Metformin, Clomiphene	PCOS	Menstrual regularity at 6 mo.	OR=1.02 (0.64-1.64)	Very Low
Laparoscopic ovarian drilling	Letrozele	PCOS	Menstrual regularity at 6 mo.	OR=1.08 (0.64-1.84)	Very Low
Laparoscopic ovarian drilling	Metformin, Letrozol	PCOS	Menstrual regularity at 6 mo.	OR=0.95 (0.49-1.81)	Very Low
Laparoscopic ovarian drilling	Metformin	PCOS	Menstrual regularity at 6 mo.	OR=1.51 (0.62-3.71)	Moderate
Laparoscopic ovarian drilling	Gonadotropins	PCOS	Improvement in androgenic symptoms 6 mo.	OR=3.02 (0.56-16.33)	Low
Laparoscopic ovarian drilling	Metformin	PCOS	Improvement in androgenic symptoms 6 mo.	OR=1 (0.42-2.37)	Low
Laparoscopic ovarian drilling	Letrozele	Infertility due to PCOS	Live birth	RR=0.72 (0.5-1.05)	Moderate
			Rate of ovarian hyperstimulation syndrome	RD=0 (-0.01-0.01)	High
Transcervical resection of endometrium using rollerball coagulation	Hormone therapy or antifibrinolytic	Heavy menstrual bleeding	Control of bleeding (cure or improvement to acceptable level) 5 y	RR=1.14 (0.97-1.34)	Very Low
			Overall satisfaction with treatment 5 y	RR=1.13 (0.94-1.37)	Very Low
Ophthalmology					
Amniotic membrane transplantation and medication	Lubrication, Antibiotics and Pressure lowering medication	Acute ocular burns	Epithelial defect 21 d post-injury	RR=0.71 (0.27-1.85)	Low
Argon laser trabeculoplasty	IOP reducing medication	Open angle glaucoma	Visual field progression	RR=0.7 (0.42-1.16)	
			Optic neuropathy progression	RR=0.71 (0.38-1.34)	
Laser surgery	intravitreal anti-VEGF	Pathological myopia	Proportion of participants with a	RR=0.32 (0.08-1.33)	Low

Zavalis EA, *et al. BMJ Open* 2024; 14:e076675. doi: 10.1136/bmjopen-2023-076675

Surgical arm	Drug arm	Disease	Outcome	Treatment effect (95% CI)	GRADE assessment
			gain of 3+ lines in BCVA at 1 y		
Surgical correction	Botulinum toxin	Strabismus	Improved ocular alignment > 10 dioptres, children	RR=1.1 (0.86-1.41)	Low
		Orthopaedic surgery			
Arthroscopic surgery	Sclerosing injection	Jumper's knee	Withdrawal rate	OR=1 (0.06-16.89)	Very Low
Open surgery	Corticosteroid injection	Trigger finger	Resolution of triggering	RR=1.48 (0.79-2.76)	Very low
Open section of the carpal ligament	NSAID and splinting or corticosteroid injections	Carpal tunnel syndrome	Improvement in clinical symptoms at three months of follow-up	RR=1.09 (0.91-1.32)	
Surgical rotator cuff repair	Non-operative treatment including corticosteroid injection and exercise	Rotator cuff tear	Pain (VAS) 12 mo	MD=-0.49 (-1.02-0.05)	Moderate
		Otolaryngology			
Surgical orbital decompression	IV Methylprednisolone 1x3 followed by oral prednisolone	Thyroid eye disease	Proportion of successes compared to the proportion of treatment failures as defined by the study authors based on the use of composite outcome scores	RR=0.16 (0.01-1.98)	
		Thoracic surgery			
Open thoracotomy	Thoracostomy drainage (with fibrinolytics)	Pleural empyema	Mortality	RR=NA (NA-NA)	Moderate
VATS	Thoracostomy drainage (with fibrinolytics)	Pleural empyema	Mortality	RR=0.8 (0.04-14.89)	Low
		Urology			
Surgical reimplantation of ureters	Antibiotics	Primary vesicoureteric reflux	Rate of patients with symptomatic UTI	RR=0.95 (0.67-1.35)	
		Vascular surgery			
Surgery including primary amputation	Thrombolysis (w/ rt-Pa or urokinase)	Acute limb ischaemia	Limb salvage (30 d)	OR=0.89 (0.27-2.91)	Low
Saphenofemoral disconnection	Therapeutic LMWH	Superficial thrombophlebitis	Symptomatic VTE	RR=5 (0.25-100)	
			Major bleeding	RR=NA	
Aspirin and carotid surgery	Aspirin	Carotid stenosis	Ipsilateral ischaemic stroke, and any operative stroke or death near occlusion	RR=0.89 (0.6-1.32)	Moderate

Abbreviations
RR: risk ratio

OR: odds ratio
HR: hazard ratio
MD: mean difference
SMD: standardized mean difference

BCC: basal cell carcinoma of the skin
GERD: Gastro-oesophageal reflux disease
GTN: glyceryl tri-nitrate
IOP: intra-ocular pressure
PCOS: polycystic ovarian syndrome
QOL: Quality of life

Zavalis EA, *et al. BMJ Open* 2024; 14:e076675. doi: 10.1136/bmjopen-2023-076675
