# Supplementary material for: Availability of evidence and comparative effectiveness for surgical versus drug interventions: an overview of systematic reviews and meta-analyses
Source: BMJ Open. 2024 Jan 9;14(1):e076675. doi: 10.1136/bmjopen-2023-076675 (PMC10810041; doi:10.1136/bmjopen-2023-076675)
Supplement: Author's manuscript [file bmjopen-2023-076675.draft_revisions.pdf]

BMJ Open

BMJ Open is committed to open peer review. As part of this commitment we make the peer review history of every article we publish publicly available.

When an article is published we post the peer reviewers' comments and the authors' responses online. We also post the versions of the paper that were used during peer review. These are the versions that the peer review comments apply to.

The versions of the paper that follow are the versions that were submitted during the peer review process. They are not the versions of record or the final published versions. They should not be cited or distributed as the published version of this manuscript.

BMJ Open is an open access journal and the full, final, typeset and author-corrected version of record of the manuscript is available on our site with no access controls, subscription charges or pay-per-view fees (http://bmjopen.bmj.com).

If you have any questions on BMJ Open's open peer review process please email info.bmjopen@bmj.com

BMJ Open

Availability of evidence and comparative effectiveness for surgical versus drug interventions: an overview of systematic reviews

Journal:	*BMJ Open*
Manuscript ID	bmjopen-2023-076675
Article Type:	Original research
Date Submitted by the Author:	15-Jun-2023
Complete List of Authors:	Zavalis, Emmanuel A.; Karolinska Institutet, Department of Learning Informatics Management and Ethics; Stanford University, Meta-Research Innovation Center at Stanford (METRICS)<br>Rameau, Anaïs; Weill Cornell Medical College, Sean Parker Institute for the Voice, Department of Otolaryngology–Head and Neck Surgery<br>Saraswathula, Anirudh; The Johns Hopkins University School of Medicine, Department of Otolaryngology–Head and Neck Surgery<br>Vist, Joachim; Karolinska Institutet, Department of Learning Informatics Management and Ethics<br>Schuit, Ewoud; Utrecht University, Julius Center; Utrecht University, Cochrane Denmark<br>Ioannidis, John; Stanford University; Stanford University, Meta-Research Innovation Center at Stanford (METRICS)
Keywords:	Decision Making, Systematic Review, SURGERY

SCHOLARONE™
Manuscripts

I, the Submitting Author has the right to grant and does grant on behalf of all authors of the Work (as defined in the below author licence), an exclusive licence and/or a non-exclusive licence for contributions from authors who are: i) UK Crown employees; ii) where BMJ has agreed a CC-BY licence shall apply, and/or iii) in accordance with the terms applicable for US Federal Government officers or employees acting as part of their official duties; on a worldwide, perpetual, irrevocable, royalty-free basis to BMJ Publishing Group Ltd ("BMJ") its licensees and where the relevant Journal is co-owned by BMJ to the co-owners of the Journal, to publish the Work in this journal and any other BMJ products and to exploit all rights, as set out in our licence.

The Submitting Author accepts and understands that any supply made under these terms is made by BMJ to the Submitting Author unless you are acting as an employee on behalf of your employer or a postgraduate student of an affiliated institution which is paying any applicable article publishing charge ("APC") for Open Access articles. Where the Submitting Author wishes to make the Work available on an Open Access basis (and intends to pay the relevant APC), the terms of reuse of such Open Access shall be governed by a Creative Commons licence – details of these licences and which Creative Commons licence will apply to this Work are set out in our licence referred to above.

Other than as permitted in any relevant BMJ Author's Self Archiving Policies, I confirm this Work has not been accepted for publication elsewhere, is not being considered for publication elsewhere and does not duplicate material already published. I confirm all authors consent to publication of this Work and authorise the granting of this licence.

For peer review only

Availability of evidence and comparative effectiveness for surgical versus drug interventions: an overview of systematic reviews

Emmanuel A. Zavalis[1,2*], Anaïs Rameau[3*], Anirudh Saraswathula[4*], Joachim Vist[1], Ewoud Schuit[5,6], John P. A. Ioannidis[2,7]

*co-first authors

1 Department of Learning Informatics Management and Ethics, Karolinska Institutet, Stockholm, Sweden

2 Meta-Research Innovation Center at Stanford (METRICS), Stanford University, Stanford, CA, USA

3 Sean Parker Institute for the Voice, Department of Otolaryngology–Head and Neck Surgery, Weill Cornell Medical College, New York, NY, USA

4 Department of Otolaryngology–Head and Neck Surgery, Johns Hopkins University School of Medicine, Baltimore, MD, USA

5 Julius Center, University Medical Center Utrecht, Utrecht University, Utrecht, the Netherlands

6 Cochrane Netherland, University Medical Center Utrecht, Utrecht University, Utrecht, the Netherlands

7 Stanford Prevention Research Center, Department of Medicine, and Department of Epidemiology and Population Health, Stanford University School of Medicine, Stanford, CA, USA

Corresponding author:
Emmanuel A. Zavalis
E-mail: emmanuel.zavalis@ki.se

Word count: 2883
Publication history: A preprint of the manuscript has been deposited in medRxiv: doi: *https://doi.org/10.1101/2023.01.30.23285207*

For peer review only

Declarations

Ethics approval and consent to participate

Not applicable

Consent for publication

Not applicable

Availability of data and materials

The dataset supporting the conclusions of this article, and the used code is available in the Open Science Framework repository *https://www.doi.org/10.17605/OSF.IO/RK7HU* .

Conflict of interest

Anaïs Rameau is a medical advisor for Perceptron Health, Inc.

Funding

The work of John Ioannidis has been funded by an unrestricted gift from Sue and Bob O'Donnell. Anaïs Rameau is supported by a Paul B. Beeson Emerging Leaders Career Development Award in Aging (K76 AG079040) from the National Institute on Aging and by the Bridge2AI award (OT2 OD032720) from the NIH Common Fund. Anirudh Saraswathula is supported by the National Institute on Deafness and Other Communication Disorders training grant 2T32DC000027. Ewoud Schuit gratefully acknowledges financial contribution for his research by the Netherlands Organisation for Scientific Research (project 825.14.001).

Authors' contributions

AR, AS, EAZ and JPAI developed the idea, EAZ and JPAI interpreted the review data. ES aided in the statistical analysis. All authors reviewed the manuscript and have edited and approved the submission.

Acknowledgements

Not applicable

For peer review only - http://bmjopen.bmj.com/site/about/guidelines.xhtml

Abstract

Objectives. To examine the prevalence of comparisons of surgery to drug regimens, the strength of evidence of such comparisons, and whether surgery or the drug intervention was favored.

Design. Systematic review of systematic reviews (umbrella review)

Data sources. Cochrane Database of Systematic Reviews (CDSR)

Methods and analysis. Using the search term "surg*" in CDSR, we retrieved systematic reviews of surgical interventions. Abstracts were subsequently screened to find systematic reviews that aimed to compare surgical to drug interventions; and then, among them, those that included any randomized controlled trials (RCTs) for such comparisons. Trial results data were extracted manually and synthesized into random-effects meta-analyses.

Results. Overall, 188 systematic reviews intended to compare surgery versus drugs. Only 41 included data from at least one RCT (total, 165 RCTs with data) and covered a total of 103 different outcomes of various comparisons of surgery versus drugs. A GRADE assessment was performed by the Cochrane reviewers for 87 (83%) outcomes in the reviews, indicating the strength of evidence was high in 4 outcomes (4%), moderate in 22 (21%), low in 27 (26%) and very low in 33 (32%). Based on 95% confidence intervals, the surgical intervention was favored in 38/103 (37%), and the drugs were favored in 13/103 (13%) outcomes. Of the outcomes with high GRADE rating, only one showed conclusive superiority (sphincterotomy was better than medical therapy for anal fissure). Of the 22 outcomes with moderate GRADE rating, 6 (27%) were inconclusive, 14 (64%) were in favor of surgery, and 2 (9%) were in favor of drugs.

Conclusions. Though the relative merits of surgical versus drug interventions are important to know for many diseases, high strength randomized evidence is rare. More randomized trials comparing surgery to drug interventions are needed.

Protocol registration. *www.doi.org/10.17605/OSF.IO/RK7HU*

For peer review only - http://bmjopen.bmj.com/site/about/guidelines.xhtml

Strengths and limitations of this study

- This study is an umbrella review that examines Cochrane reviews comparing surgical to medical interventions systematically and is a start of exploring the sequestration of medical evidence.
- The full depth of the surgical Cochrane literature may not have been covered due to ongoing updates, or them not being included with our search strategy and inclusion criteria.
- The data collected and analysed in this study can be built upon further to expand our understanding of the comparative effectiveness literature, thereby mapping gaps in evidence which may need to be addressed.

For peer review only

Introduction

Many diseases are treated or managed with surgery. Some may also be addressed by pharmaceutical interventions and studying the effectiveness of these different interventions is important in optimizing shared decision-making for patients and physicians. However, the amount and certainty of the evidence we hold in healthcare is limited[1], and this situation is likely worse for surgical interventions due to serious challenges in running placebo-controlled or comparative effectiveness trials[2]. Challenges to controlled trials include unique patient anatomy, operator dependent variables such as the skill or experience of the surgeon[3–5], and the difficulty of successful blinding[6]. Due to these challenges, randomized controlled trials (RCTs) in surgery are less common than in non-surgical medical specialties. Although there have been calls to strengthen the quality of the evidence in surgery[2, 7, 8], these challenges have resulted in relatively few RCTs assessing surgical interventions, particularly in comparison to medical treatments.

A summary of the existing body of evidence on surgical versus medical interventions across diseases does not exist in the literature. To synthesize this existing body of evidence is of paramount importance to evidence-based care and informed decisions in the clinic where surgery or drugs are available interventions. To find RCTs comparing surgical vs. pharmaceutical interventions, we conducted an umbrella review (an overview of systematic reviews) [9, 10] by searching the Cochrane Database of Systematic Reviews for reviews considering comparisons of surgery to drugs, analyze the strength of the evidence and evaluate results of these comparisons. Finally, we explored whether results favoring surgery were more likely to be published in the surgical literature.

For peer review only - http://bmjopen.bmj.com/site/about/guidelines.xhtml

Materials and Methods

The protocol for the data collection, and analysis was pre-registered on the Open Science Framework website (doi: 10.17605/OSF.IO/RK7HU).

Search strategy and selection criteria

We queried the Cochrane Database of Systematic Reviews using the term "surg*" in "Title/Abstract/Keywords" on April 25, 2022. Inclusion criteria for reviews were consideration of RCTs and comparing a surgical to a drug intervention.

A surgical intervention was defined as a procedural technique aiming to change anatomy to treat or alleviate a pathology or symptom (including dermatological excisions). We excluded endoscopic and endovascular procedures since many of them are performed by medical rather than surgical specialists. A drug intervention was defined as a treatment that utilized a non-supplement and non-vitamin, pharmaceutical agent. Dental procedures, radiation treatment, as well as comparisons of surgery vs. no treatment or only placebo were excluded from our study. Cochrane reviews that intended to compare surgical and pharmaceutical interventions were considered even in cases where the review was unsuccessful in finding any such comparisons.

As many surgical procedures also require drug regimens (e.g., pre-operatively or as background treatment), we allowed comparisons where the surgical arm including a drug intervention was compared to a drug intervention as well. Comparisons of surgery to surgery plus drugs were not

For peer review only - http://bmjopen.bmj.com/site/about/guidelines.xhtml

eligible, as both arms used surgery.

The articles' abstracts were reviewed by EAZ, and JV who coded the reviews independently for eligibility and then sought to reach a consensus. Remaining differences were mediated by JPAI.

Main outcomes

The main outcomes assessed in this umbrella review were the number of Cochrane systematic reviews that considered comparisons of surgical and drug interventions, and the number of systematic reviews that found any eligible RCTs comparing a surgical and a drug arm. The strength of evidence of the existing comparison was also treated as a main outcome, as were the direction of effects in the review assessments, both in the original Cochrane analysis and our standardized re-analysis).

Data extraction

EAZ extracted data for the included systematic reviews. The included systematic reviews were further classified into their corresponding surgical specialty field: cardiac surgery, dermatology, general surgery, neurosurgery, obstetrics and gynaecology, ophthalmology, orthopaedic surgery, otolaryngology, plastic surgery, thoracic surgery, urology and vascular surgery.

Whenever data were available from at least one RCT comparing a surgical to a drug arm, we identified the primary outcome(s) of the systematic review for the eligible comparison(s) by examining the methods section of the systematic review, and classified it as either mortality, composite or non-mortality. Data, in the form of contingency tables or means, standard

For peer review only - http://bmjopen.bmj.com/site/about/guidelines.xhtml

deviations and number of participants in each arms, from individual RCTs were then collected from Cochrane eligible reviews. We also collected information on GRADE assessments for the eligible comparisons and outcomes and the summary effect size as well as the 95% confidence interval of the effect for the eligible comparison outcomes. Reviews that found no RCT of drugs to surgery were tabulated as having no data.

Meta-analysis

As Cochrane reviewers may have used different statistical models in each topic to combine the results of RCTs in meta-analyses we aimed for standardization. To achieve it, we recalculated the summary effect size and heterogeneity for each topic using a random effects model following the Hartung-Knapp-Sidik-Jonkman approach[11, 12] so that all outcomes/topics would be analyzed with the same statistical methods. The modified Haldane-Anscombe continuity correction was used, i.e. when studies had no event in either the surgical or the drug arm we added 0.5 to the entire contingency table of the specific study[13].

The analysis of the data was performed using R version 4.1.3 (2022-03-10), with assessment of statistical significance using a threshold for α of 0.005, as previously proposed[14]. The Wilson approach was used for confidence intervals (99.5%) created for the primary outcomes.

Additions to the protocol

The original pre-registered protocol can be found in *https://osf.io/p9x3j*. Some additions were made during the process of conducting this umbrella review. For each systematic review, we noted the search date to understand how old they might be. We also extracted the year of

For peer review only - http://bmjopen.bmj.com/site/about/guidelines.xhtml

publication of each RCT to capture how recent the evidence was. Finally, we extracted the specialty orientation of the journal, in which the RCT was published, using the categories “mostly surgical”, “general”, and “mostly non-surgical”. The category “mostly surgical" includes those journals that have "surgery" in their title, those that have the name of a surgical specialty in their title, and those affiliated with a surgical society. The category "general" pertains to journals that cover all of medicine and its specialties, surgical and non-surgical. The category "mostly non-surgical" includes all the remaining journals. We assessed whether the direction of effects (favoring surgery or favoring drug) was associated with the type of journal, hypothesizing that RCTs published in mostly surgical journals may be more likely than other journals to favor surgery.

Patient and Public Involvement

No patients were involved in the design and conduct of this umbrella review

For peer review only - http://bmjopen.bmj.com/site/about/guidelines.xhtml

Results

Search results

The selection flowchart for Cochrane systematic reviews is represented in Figure 1. The search strategy retrieved 2495 articles from the Cochrane Database of Systematic Reviews. Among them, 440 were excluded by an automated search for withdrawn reviews and of studies with no mention of the word surgery and any of its variations in the abstract. Further manual inspection of titles and abstracts resulted in 223 Cochrane reviews being potentially eligible. Upon full-text evaluation, 35 were excluded: in 5 reviews, the surgical and drug treatments were not in separate arms and hence they were not an eligible head-to-head comparison[15–19]; in 7 reviews, there was no surgical intervention arm[20–26]; in 17 reviews, there was no drug intervention [27–32, 32–42]); 2 reviews were excluded for evaluating an endoscopic intervention [43, 44]; 3 reviews were excluded for evaluating an endovascular intervention [45–47]; and finally 1 review was excluded for being an umbrella review[48].

Therefore, 188 Cochrane reviews were found to meet the inclusion criteria (Supplemental Digital Content data file 1). Of those, 147 Cochrane reviews aimed to investigate surgical versus drug interventions but were unable to find any RCTs meeting their selection criteria. The remaining 41 reviews contained data for at least one RCT in at least one head-to-head comparison of a surgical versus a drug intervention arm (22% (99.5% CI 14 to 31%)).

The 188 reviews covered all major surgical specialties (Supplementary Table 1), with the most commonly represented specialties being general surgery (n=35), obstetrics and gynecology (n=31), ophthalmology (n=25), orthopedic surgery (n=23) and otolaryngology (n=23). No

For peer review only - http://bmjopen.bmj.com/site/about/guidelines.xhtml

significant difference was found across specialties in the proportion of reviews that contained data from at least one RCT for a surgery versus drug comparison (Fisher's exact p=0.62).

Comparative treatment effect for surgery versus drug comparisons

The 41 eligible reviews with data included 103 comparisons of surgery versus drug treatments with data on various primary outcomes (Table 1), and they included data from a total of 165 RCTs with a total of 295 primary outcome assessments. For the 165 trials, the median publication year was 2005 and the interquartile range was 1994 to 2016. The median search date year of the eligible reviews was 2016 (interquartile range, 2010 to 2022).

Based on the 95% confidence interval of the summary estimate obtained by the Cochrane review authors, surgery was more effective in 36 of the 103 outcomes of various comparisons (35% (99.5% CI 23 to 49%)), and drugs were more effective in 15 (15% (99.5% CI 6 to 26%)). Fifty two (50% (99.5 CI% 37 to 64%)) outcomes were inconclusive. The respective numbers were 1/12 (8%), 1/12 (8%), and 10/12 (83%) for mortality outcomes; 3/11 (27%), 3/11 (27%) and 5/11 (46%) for composite outcomes; and 32/80 (40%), 11/80 (14%), and 37/80 (46%) for non-mortality outcomes.

When we standardized the meta-analyses to use the same random effects method for all analyses, surgery was favored in 28/103 outcomes (32%), drugs were favored in 9/103 (10%) outcomes and 66/103 (58%) outcomes were inconclusive. The respective numbers were 1/12 (8%), 0/12 (0%), and 11/12 (92%) for mortality outcomes; 3/11 (18%), 2/11 (27%) and 6/11 (55%) for composite outcomes; and 24/80 (30%) 7/80 (9%), and 49/80 (61%) for non-mortality outcomes.

For peer review only - http://bmjopen.bmj.com/site/about/guidelines.xhtml

Table 2 shows the topics for which the surgical intervention was found to be more effective and Table 3 shows those where the drug arm was found to be more effective, all according to the Cochrane authors' analysis. Supplementary Table 2 does the same for the topics for which the comparisons were inconclusive.

Strength of evidence according to GRADE

GRADE assessment of the strength of the evidence showed high rating for 4 outcomes (4%), moderate for 22 (21%), low for 27 (26%), and very low for 33 (32%). No GRADE assessment was performed for 17 (17%) outcomes.

According to GRADE assessments, only cardiac surgery, obstetrics and gynecology and general surgery interventions had high GRADE ratings. Otolaryngology and dermatology had many moderate ratings. Almost all other GRADE ratings were low or very low (Table 4).

Of the four outcomes with high GRADE rating, sphincterotomy for anal fissure showed superiority over medical treatment while the other three comparisons were inconclusive. Of the 22 outcomes with moderate GRADE rating, 6 (27%) were inconclusive, 14 (64%) were in favor of surgery, and 2 (9%) were in favor of the drug regimen according to the calculations of the Cochrane authors (14 (64%), were inconclusive, 7 (32%) favored the surgical arm and 1 (5%) were in favor of the drug regimen according to our standard random-effects calculations).

Results of RCTs according to journal of publication

For peer review only - http://bmjopen.bmj.com/site/about/guidelines.xhtml

Of the 165 eligible RCTs (295 outcome assessments), 73 RCTs (133 assessments) were published in mostly surgical journals, 38 RCTs (69 assessments) in general journals, and 54 RCTs (93 assessments) in mostly non-surgical journals. Based on 95% confidence intervals for the assessments of RCTs published in mostly surgical journals, 40/133 (30%) were in favor of surgery, 14/133 (11%) were in favor of drugs, and 79/133 (59%) were inconclusive. The respective numbers for the assessments of RCTs published in general journals were 27/69 (39%), 5/69 (7%), and 37/69 (53%); and for the assessments of RCTs published in mostly non-surgical journals they were 22/93 (24%), 15/93 (16%), and 56 (60%), respectively. The proportion of RCTs favoring surgery was not significantly higher in mostly surgical journals (30%) compared to other journals (39% and 24% for general and non-surgical journals respectively) (p=0.18 by Fisher's exact test).

For peer review only - http://bmjopen.bmj.com/site/about/guidelines.xhtml

Discussion

Main findings

In a subset of Cochrane reviews that aimed to compare surgery to drugs we found that only 1 in 5 systematic reviews that had shown interest in such comparisons eventually found data from any RCTs for comparisons of the two modes of interventions. Furthermore, the majority of the comparisons where RCTs of surgery versus drugs had inconclusive results, and also had low or very low strength of the evidence on GRADE assessments. Anal fissure was the only disease in our sample that had high GRADE evidence and a direction of effect indicating that one intervention (sphincterotomy) was more effective. Consequently, in the vast majority of cases where surgical and pharmaceutical interventions are available for treatment, an evidence-based decision in the clinic is difficult. Our secondary post hoc analysis of the type of journal where the eligible RCTs were published showed that results published in surgical journals were not necessarily more prone to favor the surgical arm of an RCT over the pharmaceutical arm.

Strengths

This study covers the entire Cochrane database which is considered a high-quality comprehensive collection of systematic reviews. Cochrane reviews tend to address questions typically asked in routine clinical practice and underpin many clinical guideline recommendations, making this sample all the more relevant to everyday practice [49]. Another strength of this study is that all surgical specialties were included. This is, therefore, to our knowledge the first project aiming to assess the extent of comparative evidence for surgery versus pharmacotherapy for a diverse spectrum of diseases.

For peer review only - http://bmjopen.bmj.com/site/about/guidelines.xhtml

Limitations

Our analysis has several limitations. First, our pre-defined inclusion criteria excluded non-pharmacological medical interventions. Several comparisons may be found in the literature where surgery is compared against non-surgical non-pharmacological medical interventions, such as CPAP or radiotherapy. We also excluded endovascular and endoscopic procedures since they may be performed by surgical and medical specialists. These eligibility choices aimed to achieve some homogeneity in a project that is by definition already very heterogeneous. The use of an algorithm to filter out papers with no mention of the word surgery as well as the search strategy itself may have led to us missing reviews that discuss a particular surgical procedure but never explicitly mention the word surgery but merely the name of the intervention.

Second, we focused exclusively on RCTs, but other types of evidence, e.g., non-randomized controlled trials, or uncontrolled clinical trials may also exist and sometimes their results may be compelling enough to deem a randomized study unnecessary. Such unquestionable superiority in the absence of randomized evidence is however unlikely [50]. Efforts such as IDEAL [8] have laid out much of the groundwork for performing RCTs in surgical research, yet a dearth of RCTs in the surgical realm of research persists to this day.

Third, only one database (Cochrane Database of Systematic Reviews) was used for this study and we did not examine non-Cochrane meta-analyses published as journal articles. While the database aims to be all-inclusive, there are still some topics in medical and surgical care that have not been covered by Cochrane reviews.

For peer review only - http://bmjopen.bmj.com/site/about/guidelines.xhtml

Fourth, it is possible that within the same disease, subgroups of patients may be eligible only for medical or only for surgical treatment, or that one or the other approach is much better only for specific subgroups. With the dearth of evidence we found for the overall analysis, identification of such subgroup effects would be unlikely and error-prone.

Context of these findings

Sequestration between different disciplines and specialties[51] may lead to isolation of specialists which use different tools, and this may lead to a lack of comparisons of the treatments that each specialty uses. Each specialty may have its own community, journals, meetings, and research agenda, limiting communication between different specialists even though they may be dealing with the same disease from different angles and with different therapeutic sets. This lack of communication may also be due to differences in mentorship and the trend of sub-specialization in medical training separating clinicians and their practices even further [52], or to differing incentive structures.

Prior literature comparing surgical and medical interventions has assessed specific treatments, such as that for basal cell carcinoma[51], and demonstrated that sequestration was prominent. Despite a large number of trials, almost all of them compared medical interventions among themselves, or surgical interventions among themselves, rather than comparing between these two groups of treatment even though both groups of treatment could have been used. Our work shows that this issue of sequestration is widespread in surgical vs. pharmaceutical interventions.

Conclusion

For peer review only - http://bmjopen.bmj.com/site/about/guidelines.xhtml

This study suggests that comparisons of pharmaceutical and surgical interventions are infrequent. Even accepting the difficulties in performing RCTs involving surgical interventions, our results still indicate a need for more comparative effectiveness research and for improved communication between surgical and medical specialties to bridge this gap in evidence. There are, of course, barriers to this. Head-to-head comparisons of treatments are often disfavored by manufacturers leery of jeopardizing their product against that of a competitor [53, 54], and incentives unfortunately exist for both surgical and medical practitioners to promote treatments they are able to offer. Moving forward, both medical and surgical professional societies should collaborate to design fair and unbiased trials, and funders should also keep such research on their radars to try and overcome these structural obstacles.

Future research

Future clinical research should try to expand the scope, volume, and methodological rigor of comparative evidence on surgical versus medical interventions. This work should involve both surgical and medical specialists and should also incorporate patient preferences. Long-term patient-centered outcomes, including both benefits and harms should become available to put surgical and medical practices into proper perspective.

For peer review only - http://bmjopen.bmj.com/site/about/guidelines.xhtml

Table 1. Eligible comparisons of surgical versus medical interventions

Surgical arm	Drug arm	Disease	Outcomes (studies/N)
		Cardiac surgery	
Transmyocardial lazer revascularization	Continued medication	Refractory angina	Angina reduction (7/1053)<br>Overall mortality (7/1053)<br>Postoperative mortality (30 d) (6/967)
Surgical closure	IV indomethacin	Patent ductus arteriosus	Death before discharge (1/154)
		Dermatology	
Surgical excision	Imiquimod	BCC	Recurrence (3 y) (1/501)<br>Recurrence (5 y) (1/501)<br>Observer-rated good/excellent cosmetic outcome (1/501)<br>Patient-rated good/excellent cosmetic outcome (1/501)
Surgical excision	MAL-PDT	BCC	Recurrence (3 y) (1/68)<br>Observer-rated good/excellent cosmetic outcome (2/351)<br>Patient-rated good/excellent cosmetic outcome (2/351)
Surgical excision	ALA-PDT	BCC	Recurrence (3 y) (1/173)<br>Recurrence (5 y) (1/173)
		General surgery	
Lateral internal sphincterotomy	Medical therapy (mainly GTN Isosorbide dinitrate and Botox)	Anal fissure	NON-Healing (persistence or recurrence) 2 mo. (15/979)
Pancreatic resection	Chemoradiotherapy	Pancreatic cancer	Overall mortality (5 y) (2/98)
Oesophagectomy	Chemoradiotherapy and/or radiotherapy	Oesophageal cancer	Short-term mortality (5/689)<br>Long-term mortality (3/511)
Laparoscopic fundoplication	Protein pump inhibitors	GERD	Serious adverse event (3 months) (1/80)<br>Short-term health-related QOL (1/165)<br>Medium-term health-related QOL (1/62)<br>Health-related QOL:<br><1 y (3/605)<br>1-5 y (3/323)<br>GORD-specific QOL:<br><1 y (4/1160)<br>1-5 y (3/994)<br>Serious adverse events (2/637)
Surgery	Tamoxifen	Primary breast cancer	Overall survival (3/495)
		Neurosurgery	
Decompressive surgery	Prednisolone	Leprosy	Change in motor or sensory score after one year (1/57)<br>Proportion of ulnar nerves with:<br>sensory improvement after one year (1/62), and<br>motor improvement after one year (1/62)
Epilepsy surgery	Continued antiepileptic drugs	Epilepsy	Proportion free from seizures (1 y) (2/196)<br>Proportion free from all seizures including auras (1 y) (1/80)
Decompressive craniectomy	Medical treatment (including barbiturates)	High ICP in closed TBI	Neurological unfavourable outcome 6 mo (3/571)<br>Mortality 6 mo (3/571)

For peer review only - http://bmjopen.bmj.com/site/about/guidelines.xhtml

Surgical arm	Drug arm	Disease	Outcomes (studies/N)
Surgical decompression	Osmotic agents, blood pressure control, and glucose control	Cerebral oedema in acute ischaemic stroke	Death at the end of follow-up (3/134)
Surgical decompression	Dexamethasone, antihypertensives and intermittent diuresis	Primary supratentorial intracerebral haemorrhage	Death or dependence at end of follow up (9/1994)
		Obstetrics and gynaecology	
Suction aspiration	Vaginal suppositories or im inj. of 9-methylene-PGE2	Abortion	Abortion not completeted with intended method (2/472)
			Ongoing pregnancy (2/472)
			Pelvic infection (1/419)
Suction aspiration	Misoprostol	Abortion	Complete miscarriage (22/5285)
			Composite outcome of death or serious complication (9/2146)
Suction aspiration	Vaginal or oral misoprostol	Abortion	Complete miscarriage (15/3862)
			Surgical evacuation (13/3070)
			Death or serious complication (5/1248)
Suction aspiration	Misoprostol and mifepristone	Abortion	Complete miscarriage (2/716)
			Composite outcome of death or serious complication (1/618)
Dilatation and curretage	Misoprostol	Abortion	Complete miscarriage (1/107)
			Composite outcome of death or serious
Dilation and evacuation	Misoprostol	Abortion	Combined major and minor complications (1/94)
Laparoscopic ovarian drilling	Medical ovulation induction	Infertility due to PCOS	Live birth (9/1015)
			Multiple pregnancy (14/1161)
Laparoscopic ovarian drilling	Letrozele	Infertility due to PCOS	Live birth (3/548)
			Rate of ovarian hyperstimulation syndrome (1/250)
Laparoscopic ovarian drilling	Gonadotropins	PCOS	Menstrual regularity at 6 mo. (1/35)
			Improvement in androgenic symptoms 6 mo. (1/126)
Laparoscopic ovarian drilling	Metformin, clomiphene	PCOS	Menstrual regularity at 6 mo. (2/332)
Laparoscopic ovarian drilling	Letrozele	PCOS	Menstrual regularity at 6 mo. (1/260)
Laparoscopic ovarian drilling	Metformin, letrozele	PCOS	Menstrual regularity at 6 mo. (1/156)
Laparoscopic ovarian drilling	Metformin	PCOS	Menstrual regularity at 6 mo. (2/236)
			Improvement in androgenic symptoms 6 mo. (1/50)
Transcervical resection of endometrium using rollerball coagulation	Hormone therapy or antifibrinolytic	Heavy menstrual bleeding	Control of bleeding (cure or improvement to acceptable level) 4 mo. (1/186)
			Control of bleeding (cure or improvement to acceptable level) 2 y (1/173)
			Control of bleeding (cure or improvement to acceptable level) 5 y (1/140)
			Overall satisfaction with treatment 4 mo. (1/186)
			Overall satisfaction with treatment 2 y (1/173)
			Overall satisfaction with treatment 5 y (1/141)
			Adverse events at 4 months (1/186)
		Ophthalmology	
Amniotic membrane transplantation and medication	Lubrication, antibiotics and pressure lowering medication	Acute ocular burns	Epithelial defect 21 d post-injury, and Visual acuity at final follow-up (1/68)
Laser surgery	intravitreal anti-VEGF	Pathological myopia	Change in best corrected visual acuity (1/36)
			Proportion of participants with a gain of 3+ lines in BCVA at 1 y (1/36)

For peer review only - http://bmjopen.bmj.com/site/about/guidelines.xhtml

Surgical arm	Drug arm	Disease	Outcomes (studies/N)
iStent	Latanoprost/timolol	Open angle glaucoma	Proportion of participants who were drop-free 6-18 mo (2/285)
Argon laser trabeculoplasty	IOP reducing medication	Open angle glaucoma	Failure to control IOP (3/735)<br>Visual field progression (2/624)<br>Optic neuropathy progression (2/264)
Surgical correction	Botulinum toxin	Strabismus	Improved ocular alignment > 10 dioptres, children (2/102), adults (1/30)
		Orthopaedic surgery	
Open section of the carpal ligament	NSAID and splinting or corticosteroid injections	Carpal tunnel syndrome	Improvement in clinical symptoms at three months of follow-up (2/245)
Open surgery	Corticosteroid injection	Trigger finger	Resolution of triggering (2/270)
Decompressive surgery with or without fusion	Epidural steroid injection	Lumbar spinal stenosis	Oswestry Disability index 6 w (1/38)<br>Pain intensity (VAS) 6 w (1/38)<br>Zurich claudication questionnaire (symptom evaluation) 6 w (1/38)
Open unilateral sympathectomy (L2-4)	IV prostanoid iloprost	Critical limb ischaemia	Complete ulcer healing w/o rest pain or major amputation (24 w) (1/162)
Surgical rotator cuff repair	Non-operative treatment including corticosteroid injection and exercise	Rotator cuff tear	Pain (VAS) 12 mo (1/56)
Arthroscopic surgery	Sclerosing injection	Jumper's knee	Knee pain (0-100, 12 mo.) (1/50)<br>Participant global assessment of success (1-100, 12 mo.) (1/50)<br>Withdrawal rate (1/52)
		Otolaryngology	
Surgical orbital decompression	IV Methylprednisolone 1x3 followed by oral prednisolone	Thyroid eye disease	Proportion of successes compared to the proportion of treatment failures as defined by the study authors based on the use of composite outcome scores (1/15)
Grommets (ventilation tubes)	Antibiotic prophylaxis	Recurrent acute otitis media	Proportion of patients who have no AOM recurrences (6 mo.) (2/96)
Tonsillectomy or adrenotonsillectomy	Watchful waiting with or without analgesics and antibiotics	Tonsillitis	Episodes of sore throat of any severity (children) (5/795)<br>Episodes of moderately or severely sore throat (children) (4/564)<br>Sore throat days (children) (5/776)<br>Episodes of sore throat of any severity (adults) (2/156)<br>Sore throat days (adults) (2/156)
		Thoracic surgery	
Open thoracotomy	Thoracostomy drainage (with fibrinolytics)	Pleural empyema	Mortality (1/30)
VATS	Thoracostomy drainage (with fibrinolytics)	Pleural empyema	Mortality (7/367)
		Urology	
Surgical reimplantation of ureters	Antibiotics	Primary vesicoureteric reflux	Rate of patients with symptomatic UTI (1/297)
		Vascular surgery	
Carotid endarterectomy and Aspirin 325 mg daily	Aspirin 325 mg daily	Asymptomatic carotid stenosis	Perioperative stroke or death, or stroke of any territory or type during follow up (2/2103)
Aspirin and carotid surgery	Aspirin	Carotid stenosis	Any stroke or operative death (3/6090)<br>Ipsilateral ischaemic stroke, and any operative stroke or death near occlusion (3/6090)
Saphenofemoral disconnection	Therapeutic LMWH	Superficial thrombophlebitis	Symptomatic VTE (1/60)<br>Major bleeding (1/60)

For peer review only

For peer review only - http://bmjopen.bmj.com/site/about/guidelines.xhtml

Surgical arm	Drug arm	Disease	Outcomes (studies/N)
Surgery including primary amputation	Thrombolysis (w/ rt-Pa or urokinase)	Acute limb ischaemia	Limb salvage (30 d) (3/841)

Abbreviations

BCC: basal cell carcinoma of the skin
GERD: Gastro-oesophageal reflux disease
GTN: glyceryl tri-nitrate
IOP: intra-ocular pressure
PCOS: polycystic ovarian syndrome
QOL: Quality of life

For peer review only

For peer review only - http://bmjopen.bmj.com/site/about/guidelines.xhtml

Table 2. Comparisons where the surgical treatment was superior to the drug treatment

Surgical arm	Drug arm	Disease	Outcome	Treatment effect (95% CI)	GRADE assessment
			Cardiac surgery		
Transmyocardial lazer revascularization	Continued medication	Refractory angina	Angina reduction	OR=4.63 (3.43-6.25)	Low
			Dermatology		
Surgical excision	Imiquimod	BCC	Recurrence (3 y)	RR=0.1 (0.03-0.31)	Moderate
			Recurrence (5 y)	RR=0.13 (0.05-0.36)	Moderate
Surgical excision	MAL-PDT	BCC	Recurrence (3 y)	RR=0.04 (0-0.61)	Low
Surgical excision	ALA-PDT	BCC	Recurrence (3 y)	RR=0.09 (0.02-0.38)	Moderate
			Recurrence (5 y)	RR=0.08 (0.02-0.34)	Moderate
			General surgery		
Laparoscopic fundoplication	Protein pump inhibitors	GERD	GORD-specific QOL (<1 y)	SMD=0.58 (0.46-0.7)	Low
Lateral internal sphincterotomy	Medical therapy (mainly GTN and Botox)	Anal fissure	Non-Healing (persistence or recurrence) 2 mo.	OR=0.11 (0.06-0.23)	High
			Neurosurgery		
Epilepsy surgery	Continued antiepileptic drugs	Epilepsy	Proportion (%) free from seizures (1 y)	RR=9.78 (4.73-20.2)*	Low
			Proportion free from all seizures incl. auras (1 y)	RR=15 (2.08-108.23)	Very Low
Surgical decompression	Osmotic agents, blood pressure control, and glucose control	Cerebral oedema in acute ischaemic stroke	Death at the end of follow-up	OR=0.19 (0.09-0.37)	
Surgical decompression	Dexamethasone, antihypertensives and intermittent diuresis	Primary supratentorial intracerebral haemorrhage	Death or dependence at end of follow up	OR=0.71 (0.58-0.88)	
			Obstetrics and gynaecology		
Suction aspiration	Misoprostol	Abortion	Complete miscarriage	RR=1.11 (1.06-1.17)	Very Low
			Complete miscarriage	RR=1.04 (1.02-1.06)	Very Low
Dilatation and curettage	Misoprostol	Abortion	Complete miscarriage	RR=1.18 (1.1-1.27)*	Very Low
Dilatation and evacuation	Misoprostol	Abortion	Combined major and minor complications	OR=0.12 (0.03-0.46)	
Laparoscopic ovarian drilling	Medical ovulation induction	Infertility due to PCOS	Multiple pregnancy	OR=0.34 (0.18-0.66)	Moderate

For peer review only - http://bmjopen.bmj.com/site/about/guidelines.xhtml

Surgical arm	Drug arm	Disease	Outcome	Treatment effect (95% CI)	GRADE assessment
Laparoscopic ovarian drilling	Gonadotropins	PCOS	Menstrual regularity at 6 mo.	OR=19.2 (3.17-116)	Very Low
Transcervical resection of endometrium using rollerball coagulation	Hormone therapy or antifibrinolytic	Heavy menstrual bleeding	Control of bleeding (cure or improvement to acceptable level) 4 mo.	RR=2.66 (1.94-3.64)	Moderate
			Control of bleeding (cure or improvement to acceptable level) 2 y	RR=1.29 (1.06-1.57)	Low
			Overall satisfaction with treatment 4 mo.	RR=2.8 (1.96-3.99)	Moderate
			Overall satisfaction with treatment 2 y	RR=1.4 (1.13-1.74)	Moderate
			Adverse events at 4 months	RR=0.26 (0.15-0.46)	Moderate
Ophthalmology					
Surgical correction	Botulinum toxin	Strabismus	Improved ocular alignment > 10 dioptres, adults	RR=2.63 (1.18-5.9)	Low
iStent	Latanoprost/timolol	Open angle glaucoma	Proportion of participants who were drop-free 6-18 mo	RR=125 (17.8-884)	Very low
Argon laser trabeculoplasty	IOP reducing medication	Open angle glaucoma	Failure to control IOP	RR=0.8 (0.71-0.91)	
Orthopaedic surgery					
Arthroscopic surgery	Sclerosing injection	Jumper's knee	Knee pain (0-100, 12 mo.)	MD=-28.3 (-41.79- -14.81)	Low
			Participant global assessment of success (1-100, 12 mo.)	MD=33.9 (18.74-49.06)	Low
Decompressive surgery with or without fusion	Epidural steroid injection	Lumbar spinal stenosis	Zurich claudication questionnaire (symptom evaluation) 6 w	MD=-0.6 (-0.77- -0.43)	Low
Open unilateral sympathectomy (L2-4)	IV prostanoid iloprost		Complete ulcer healing w/o rest pain or major amputation (24 w)	RR=1.76 (1.35-2.29)	Low
Otolaryngology					
Grommets (ventilation tubes)	Antibiotic prophylaxis	Recurrent acute otitis media	Proportion of patients who have no recurrences (6 mo.)	RR=1.68 (1.07-2.65)*	Very Low
Tonsillectomy or adrenotonsillectomy	Watchful waiting with or without analgesics and antibiotics	Tonsillitis	Episodes of sore throat of any severity (children)	MD=-0.56 (-1.04- -0.07)*	Moderate
			Sore throat days (children)	MD=-5.13 (-8.03- -2.2)*	Moderate
			Episodes of sore throat of any severity (adults)	-MD=3.61 (-7.92- -0.7)*	Moderate
			Sore throat days (adults)	MD=-10.64 (-15.52- -5.76)*	Moderate

For peer review only - http://bmjopen.bmj.com/site/about/guidelines.xhtml

Surgical arm	Drug arm	Disease	Outcome	Treatment effect (95% CI)	GRADE assessment
			Vascular surgery		
Aspirin and carotid surgery	Aspirin	Carotid stenosis	Any stroke or operative death	RR=0.85 (0.77-0.95)*	Moderate

*our re-analysis using a random effects meta-analysis model shows that the 95% confidence interval includes the null (results are inconclusive)

RR: risk ratio
OR: odds ratio
HR: hazard ratio
MD: mean difference
SMD: standardized mean difference
BCC: basal cell carcinoma of the skin
GERD: Gastro-oesophageal reflux disease
GNT: glyceryl trinitrate
IOP: intra-ocular pressure
PCOS: polycystic ovarian syndrome
QOL: Quality of life

For peer review only

Table 3. Comparisons where the drug treatment was superior to the surgical treatment

Surgical arm	Drug arm	Disease	Outcome	Treatment effect (95% CI)	GRADE assessment
		Dermatology			
Surgical excision	Imiquimod	BCC	Observer-rated good/excellent cosmetic outcome	RR=0.59 (0.47-0.74)	Low
Surgical excision	MAL-PDT	BCC	Observer-rated good/excellent cosmetic outcome	RR=0.85 (0.79-0.92)*	Moderate
Surgical excision	MAL-PDT	BCC	Patient-rated good/excellent cosmetic outcome	RR=0.53 (0.44-0.65)*	Moderate
		General surgery			
Oesophagectomy	Chemoradiotherapy and/or radiotherapy	Oesophageal cancer	Serious adverse event (3 months)	RR=1.73 (1.11-2.67)*	Very Low
			Short-term health-related QOL	MD=0.93 (0.24-1.62)	Very Low
Laparoscopic fundoplication	Protein pump inhibitors	GERD	Serious adverse events	RR=1.46 (1.01-2.11)	Very Low
Pancreatic resection	Chemoradiotherapy	Pancreatic cancer	Overall mortality (5 y)	HR=2.63 (1.72-4)*	Very Low
		Obstetrics and gynaecology			
Laparoscopic ovarian drilling	Medical ovulation induction	Infertility due to PCOS	Live birth	OR=0.71 (0.54-0.92)	Low
Suction aspiration	Vaginal or oral misoprostol	Abortion	Surgical evacuation	RR=20 (9.1-50)	Very Low
		Ophthalmology			
Laser surgery	intravitreal anti-VEGF	Pathological myopia	Change in best corrected visual acuity	MD=0.22 (0.01-0.43)*	Low
Amniotic membrane transplantation and medication	Lubrication, Antibiotics and Pressure lowering medication	Acute ocular burns	Visual acuity at final follow-up	MD=-0.83 (-1.32- -0.34)	Very Low
		Orthopaedic surgery			
Decompressive surgery with or without fusion	Epidural steroid injection	Lumbar spinal stenosis	Oswestry Disability index 6 w	MD=5.7 (0.57-10.83)	Low
			Pain intensity (VAS) 6 w	MD=2.4 (1.92-2.88)	Low
		Otolaryngology			
Tonsillectomy or adrenotonsillectomy	Watchful waiting with or without analgesics and antibiotics	Tonsillitis	Episodes of moderately or severely sore throat (children)	MD=0.62 (0.22-1.03)*	Low
		Vascular surgery			
Carotid endarterectomy and Aspirin 325 mg daily	Aspirin 325 mg daily	Asymptomatic carotid stenosis	Perioperative stroke or death, or stroke of any territory or type during follow up	RR=6.49 (2.53-16.61)	

For peer review only - http://bmjopen.bmj.com/site/about/guidelines.xhtml

*our re-analysis using a random effects meta-analysis model shows that the 95% confidence interval includes the null (results are inconclusive)

RR: risk ratio
OR: odds ratio
HR: hazard ratio
MD: mean difference
BCC: basal cell carcinoma of the skin
GERD: Gastro-oesophageal reflux disease
PCOS: polycystic ovarian syndrome

For peer review only

For peer review only - http://bmjopen.bmj.com/site/about/guidelines.xhtml

Table 4. GRADE assessment across specialties

Specialty	Very Low	Low	Moderate	High	None available
Cardiac surgery	0 (0)	1 (25)	0 (0)	2 (50)	1 (25)
Dermatology	0 (0)	3 (33)	6 (67)	0 (0)	0 (0)
General surgery	9 (69)	3 (23)	0 (0)	1 (8)	0 (0)
Neurosurgery	5 (50)	2 (20)	1 (10)	0 (0)	2 (20)
Obstetrics and gynecology	14 (45)	4 (13)	7 (23)	1 (3)	5 (16)
Ophthalmology	2 (20)	5 (50)	0 (0)	0 (0)	3 (30)
Orthopaedic surgery	2 (20)	6 (60)	1 (10)	0 (0)	1 (10)
Otolaryngology	1 (14)	1 (14)	4 (57)	0 (0)	1 (14)
Thoracic surgery	0 (0)	1 (50)	1 (50)	0 (0)	0 (0)
Urology	0 (0)	0 (0)	0 (0)	0 (0)	1 (100)
Vascular surgery	0 (0)	1 (17)	2 (33)	0 (0)	3 (50)

For peer review only

For peer review only - http://bmjopen.bmj.com/site/about/guidelines.xhtml

References

1. Howick J, Koletsi D, Ioannidis JPA, Madigan C, Pandis N, Loef M, et al. Most healthcare interventions tested in Cochrane Reviews are not effective according to high quality evidence: a systematic review and meta-analysis. J Clin Epidemiol. 2022;:S0895-4356(22)00100-7.

2. Ergina PL, Cook JA, Blazeby JM, Boutron I, Clavien P-A, Reeves BC, et al. Challenges in evaluating surgical innovation. The Lancet. 2009;374:1097–104.

3. Maruthappu M, Gilbert BJ, El-Harasis MA, Nagendran M, McCulloch P, Duclos A, et al. The influence of volume and experience on individual surgical performance: a systematic review. Ann Surg. 2015;261:642–7.

4. Martling A, Cedermark B, Johansson H, Rutqvist LE, Holm T. The surgeon as a prognostic factor after the introduction of total mesorectal excision in the treatment of rectal cancer. British Journal of Surgery. 2002;89:1008–13.

5. McGrath PD. Relation Between Operator and Hospital Volume and Outcomes Following Percutaneous Coronary Interventions in the Era of the Coronary Stent. JAMA. 2000;284:3139.

6. Gelijns AC, Ascheim DD, Parides MK, Kent KC, Moskowitz AJ. Randomized trials in surgery. Surgery. 2009;145:581–7.

7. Barkun JS, Aronson JK, Feldman LS, Maddern GJ, Strasberg SM, Balliol Collaboration, et al. Evaluation and stages of surgical innovations. Lancet. 2009;374:1089–96.

8. McCulloch P, Altman DG, Campbell WB, Flum DR, Glasziou P, Marshall JC, et al. No surgical innovation without evaluation: the IDEAL recommendations. Lancet. 2009;374:1105–12.

9. Janiaud P, Agarwal A, Tzoulaki I, Theodoratou E, Tsilidis KK, Evangelou E, et al. Validity of observational evidence on putative risk and protective factors: appraisal of 3744 meta-analyses on 57 topics. BMC Med. 2021;19:157.

10. Belbasis L, Bellou V, Ioannidis JPA. Conducting umbrella reviews. bmjmed. 2022;1:e000071.

11. Hartung J, Knapp G. A refined method for the meta-analysis of controlled clinical trials with binary outcome. Stat Med. 2001;20:3875–89.

12. IntHout J, Ioannidis JPA, Borm GF. The Hartung-Knapp-Sidik-Jonkman method for random effects meta-analysis is straightforward and considerably outperforms the standard DerSimonian-Laird method. BMC Med Res Methodol. 2014;14:25.

13. Weber F, Knapp G, Ickstadt K, Kundt G, Glass Ä. Zero-cell corrections in random-effects meta-analyses. Res Syn Meth. 2020;11:913–9.

14. Ioannidis JPA. The Proposal to Lower *P* Value Thresholds to .005. JAMA. 2018;319:1429.

For peer review only - http://bmjopen.bmj.com/site/about/guidelines.xhtml

15. Phelps PO, Abariga SA, Cowling BJ, Selva D, Marcet MM. Antimetabolites as an adjunct to dacryocystorhinostomy for nasolacrimal duct obstruction. Cochrane Database Syst Rev. 2020;4:CD012309.

16. Rees K, Beranek-Stanley M, Burke M, Ebrahim S. Hypothermia to reduce neurological damage following coronary artery bypass surgery. Cochrane Database Syst Rev. 2001;:CD002138.

17. Hoare BJ, Wallen MA, Imms C, Villanueva E, Rawicki HB, Carey L. Botulinum toxin A as an adjunct to treatment in the management of the upper limb in children with spastic cerebral palsy (UPDATE). Cochrane Database Syst Rev. 2010;2010:CD003469.

18. Shylasree TS, Bryant A, Howells RE. Chemoradiation for advanced primary vulval cancer. Cochrane Database Syst Rev. 2011;2011:CD003752.

19. Lansbury L, Leonardi-Bee J, Perkins W, Goodacre T, Tweed JA, Bath-Hextall FJ. Interventions for non-metastatic squamous cell carcinoma of the skin. Cochrane Database Syst Rev. 2010;:CD007869.

20. Moreno G, Corbalán J, Peñaloza B, Pantoja T. Topical corticosteroids for treating phimosis in boys. Cochrane Database Syst Rev. 2014;:CD008973.

21. Mitra S, Scrivens A, von Kursell AM, Disher T. Early treatment versus expectant management of hemodynamically significant patent ductus arteriosus for preterm infants. Cochrane Database Syst Rev. 2020;12:CD013278.

22. Flower A, Liu JP, Lewith G, Little P, Li Q. Chinese herbal medicine for endometriosis. Cochrane Database Syst Rev. 2012;:CD006568.

23. Görk AS, Ehrenkranz RA, Bracken MB. Continuous infusion versus intermittent bolus doses of indomethacin for patent ductus arteriosus closure in symptomatic preterm infants. Cochrane Database Syst Rev. 2008;2008:CD006071.

24. Zhao J-G, Wang J, Huang W-J, Zhang P, Ding N, Shang J. Interventions for treating simple bone cysts in the long bones of children. Cochrane Database Syst Rev. 2017;2:CD010847.

25. Bettany-Saltikov J, Weiss H-R, Chockalingam N, Taranu R, Srinivas S, Hogg J, et al. Surgical versus non-surgical interventions in people with adolescent idiopathic scoliosis. Cochrane Database Syst Rev. 2015;:CD010663.

26. Kang D, Han J, Neuberger MM, Moy ML, Wallace SA, Alonso-Coello P, et al. Transurethral radiofrequency collagen denaturation for the treatment of women with urinary incontinence. Cochrane Database Syst Rev. 2015;:CD010217.

27. Colquitt JL, Pickett K, Loveman E, Frampton GK. Surgery for weight loss in adults. Cochrane Database Syst Rev. 2014;:CD003641.

For peer review only - http://bmjopen.bmj.com/site/about/guidelines.xhtml

28. Palmer JS, Monk AP, Hopewell S, Bayliss LE, Jackson W, Beard DJ, et al. Surgical interventions for symptomatic mild to moderate knee osteoarthritis. Cochrane Database Syst Rev. 2019;7:CD012128.

29. Laupattarakasem W, Laopaiboon M, Laupattarakasem P, Sumananont C. Arthroscopic debridement for knee osteoarthritis. Cochrane Database Syst Rev. 2008;:CD005118.

30. Liu JP, Yang H, Xia Y, Cardini F. Herbal preparations for uterine fibroids. Cochrane Database Syst Rev. 2013;:CD005292.

31. Cirocchi R, Trastulli S, Pressi E, Farinella E, Avenia S, Morales Uribe CH, et al. Non-operative management versus operative management in high-grade blunt hepatic injury. Cochrane Database Syst Rev. 2015;2015:CD010989.

32. Page MJ, Massy-Westropp N, O'Connor D, Pitt V. Splinting for carpal tunnel syndrome. Cochrane Database Syst Rev. 2012;2012:CD010003.

33. Nikolaidis I, Fouyas IP, Sandercock PA, Statham PF. Surgery for cervical radiculopathy or myelopathy. Cochrane Database Syst Rev. 2010;2010:CD001466.

34. Cheuk DKL, Wong V, Wraige E, Baxter P, Cole A. Surgery for scoliosis in Duchenne muscular dystrophy. Cochrane Database Syst Rev. 2015;2015:CD005375.

35. Weitz M, Portz S, Laube GF, Meerpohl JJ, Bassler D. Surgery versus non-surgical management for unilateral ureteric-pelvic junction obstruction in newborns and infants less than two years of age. Cochrane Database Syst Rev. 2016;7:CD010716.

36. Abraham A, Kumar S, Chaudhry S, Ibrahim T. Surgical interventions for diaphyseal fractures of the radius and ulna in children. Cochrane Database Syst Rev. 2011;:CD007907.

37. Chang MY, Coleman AL, Tseng VL, Demer JL. Surgical interventions for vertical strabismus in superior oblique palsy. Cochrane Database Syst Rev. 2017;2017:CD012447.

38. Monk AP, Davies LJ, Hopewell S, Harris K, Beard DJ, Price AJ. Surgical versus conservative interventions for treating anterior cruciate ligament injuries. Cochrane Database Syst Rev. 2016;4:CD011166.

39. Gosler MW, Testroote M, Morrenhof JW, Janzing HMJ. Surgical versus non-surgical interventions for treating humeral shaft fractures in adults. Cochrane Database Syst Rev. 2012;1:CD008832.

40. Smith TO, Donell S, Song F, Hing CB. Surgical versus non-surgical interventions for treating patellar dislocation. Cochrane Database Syst Rev. 2015;:CD008106.

41. Abudou M, Chen X, Kong X, Wu T. Surgical versus non-surgical treatment for thoracolumbar burst fractures without neurological deficit. Cochrane Database Syst Rev. 2013;:CD005079.

For peer review only - http://bmjopen.bmj.com/site/about/guidelines.xhtml

42. Cataneo AJM, Cataneo DC, de Oliveira FHS, Arruda KA, El Dib R, de Oliveira Carvalho PE. Surgical versus nonsurgical interventions for flail chest. Cochrane Database Syst Rev. 2015;2015:CD009919.

43. Khalil HS, Nunez DA. Functional endoscopic sinus surgery for chronic rhinosinusitis. Cochrane Database Syst Rev. 2006;:CD004458.

44. Tóth M, Shah A, Hu K, Bunce C, Gazzard G. Endoscopic cyclophotocoagulation (ECP) for open angle glaucoma and primary angle closure. Cochrane Database Syst Rev. 2019;2:CD012741.

45. Fakhry F, Fokkenrood HJ, Spronk S, Teijink JA, Rouwet EV, Hunink MGM. Endovascular revascularisation versus conservative management for intermittent claudication. Cochrane Database Syst Rev. 2018;3:CD010512.

46. Araujo ST, Moreno DH, Cacione DG. Percutaneous thrombectomy or ultrasound-accelerated thrombolysis for initial management of acute limb ischaemia. Cochrane Database Syst Rev. 2022;1:CD013486.

47. Jenks S, Yeoh SE, Conway BR. Balloon angioplasty, with and without stenting, versus medical therapy for hypertensive patients with renal artery stenosis. Cochrane Database Syst Rev. 2014;2014:CD002944.

48. O'Connell NE, Wand BM, McAuley J, Marston L, Moseley GL. Interventions for treating pain and disability in adults with complex regional pain syndrome. Cochrane Database Syst Rev. 2013;2013:CD009416.

49. Korfitsen CB, Mikkelsen M-LK, Ussing A, Walker KC, Rohde JF, Andersen HK, et al. Usefulness of Cochrane Reviews in Clinical Guideline Development-A Survey of 585 Recommendations. Int J Environ Res Public Health. 2022;19:685.

50. Hozo I, Djulbegovic B, Parish AJ, Ioannidis JPA. Identification of threshold for large (dramatic) effects that would obviate randomized trials is not possible. Journal of Clinical Epidemiology. 2022;145:101–11.

51. Kim DD, Tang JY, Ioannidis JPA. Network geometry shows evidence sequestration for medical vs. surgical practices: treatments for basal cell carcinoma. Journal of Clinical Epidemiology. 2014;67:391–400.

52. Hirshman BR, Tang JA, Jones LA, Proudfoot JA, Carley KM, Marshall L, et al. Impact of medical academic genealogy on publication patterns: An analysis of the literature for surgical resection in brain tumor patients: Medical Academic Genealogy. Ann Neurol. 2016;79:169–77.

53. Flacco ME, Manzoli L, Boccia S, Capasso L, Aleksovska K, Rosso A, et al. Head-to-head randomized trials are mostly industry sponsored and almost always favor the industry sponsor. Journal of Clinical Epidemiology. 2015;68:811–20.

For peer review only

54. Lathyris DN, Patsopoulos NA, Salanti G, Ioannidis JPA. Industry sponsorship and selection of comparators in randomized clinical trials. European Journal of Clinical Investigation. 2010;40:172–82.

For peer review only

For peer review only - http://bmjopen.bmj.com/site/about/guidelines.xhtml

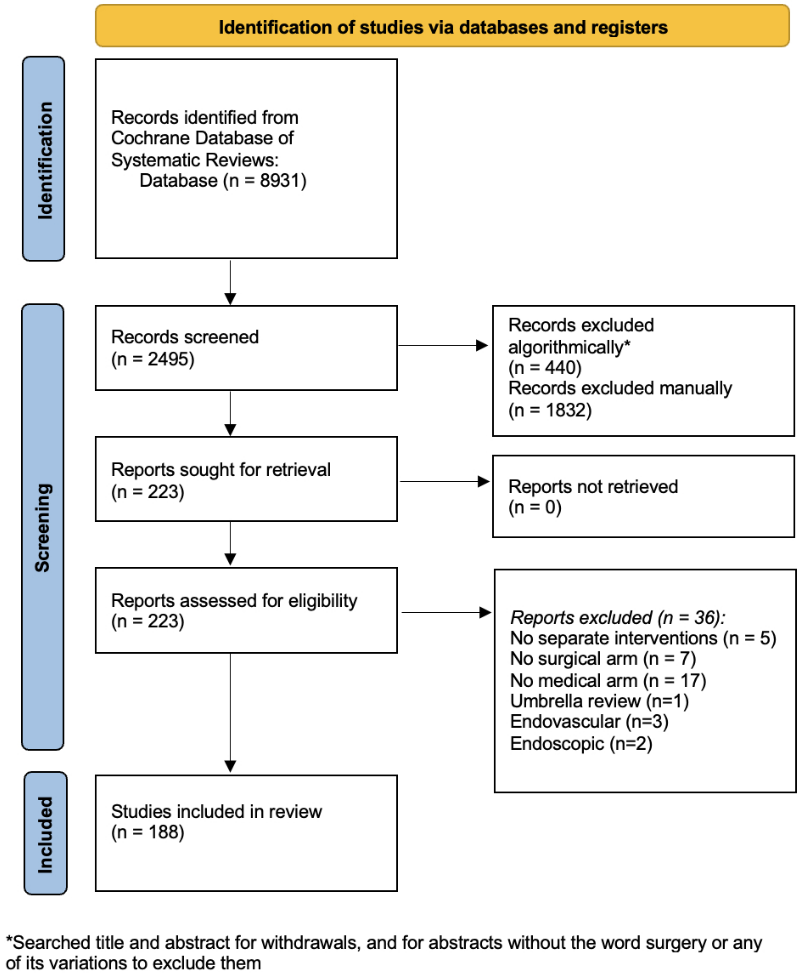

Figure 1

253x255mm (118 x 118 DPI)

Availability of evidence and comparative effectiveness for surgical versus drug interventions: an overview of systematic reviews

Emmanuel A. Zavalis[1,2*], Anaïs Rameau[3*], Anirudh Saraswathula[4*], Joachim Vist[1], Ewoud Schuit[5,6], John P. A. Ioannidis[2,7]

*co-first authors

1 Department of Learning Informatics Management and Ethics, Karolinska Institutet, Stockholm, Sweden

2 Meta-Research Innovation Center at Stanford (METRICS), Stanford University, Stanford, CA, USA

3 Sean Parker Institute for the Voice, Department of Otolaryngology–Head and Neck Surgery, Weill Cornell Medical College, New York, NY, USA

4 Department of Otolaryngology–Head and Neck Surgery, Johns Hopkins University School of Medicine, Baltimore, MD, USA

5 Julius Center, University Medical Center Utrecht, Utrecht University, Utrecht, the Netherlands

6 Cochrane Netherland, University Medical Center Utrecht, Utrecht University, Utrecht, the Netherlands

7 Stanford Prevention Research Center, Department of Medicine, and Department of Epidemiology and Population Health, Stanford University School of Medicine, Stanford, CA, USA

Supplementary Materials - Index

Supplementary Data

Supplement 1 – List of included studies *pag. 2*

Supplementary Figures and Tables

Supplementary Table 1 *pag. 7*

Supplementary Table 2 *pag. 8*

For peer review only

Supplementary Data

Supplement 1 – List of included studies

CDSR_ID	Title	Specialty	Comparison available
CD005624.PUB4	Interventions for great saphenous vein incompetence	vascular surgery	No
CD006931.PUB2	Submacular surgery for choroidal neovascularisation secondary to age-related macular degeneration	ophthalmology	No
CD002764.PUB2	Surgery for the resolution of symptoms in malignant bowel obstruction in advanced gynaecological and gastrointestinal cancer	general surgery	No
CD007119.PUB2	Interventions for restoring patency of occluded central venous catheter lumens	vascular surgery	No
CD008509.PUB3	Alpha-blockers as medical expulsive therapy for ureteral stones	urology	No
CD013085.PUB2	Balneotherapy for chronic venous insufficiency	vascular surgery	No
CD009959.PUB2	Interventions for the treatment of Frey's syndrome	otolaryngology	No
CD004008.PUB3	Interventions for trachoma trichiasis	ophthalmology	No
CD006134.PUB5	Oral contraceptives for functional ovarian cysts	obstetrics and gynecology	No
CD011650.PUB2	Management of people with early- or very early-stage hepatocellular carcinoma	general surgery	No
CD001081.PUB4	Carotid endarterectomy for symptomatic carotid stenosis	vascular surgery	Yes
CD010244.PUB2	Resection versus other treatments for locally advanced pancreatic cancer	general surgery	Yes
CD012432.PUB2	Interventions for managing medication-related osteonecrosis of the jaw	otolaryngology	No
CD010260.PUB2	Hysterectomy with radiotherapy or chemotherapy or both for women with locally advanced cervical cancer	obstetrics and gynecology	No
CD012602.PUB2	Methods for managing miscarriage: a network meta-analysis	obstetrics and gynecology	Yes
CD006983.PUB3	Decompressive surgery for treating nerve damage in leprosy	neurosurgery	Yes
CD009590.PUB2	Endometriosis: an overview of Cochrane Reviews	obstetrics and gynecology	No
CD005320.PUB2	Operative and non-operative treatment options for dislocation of the hip following total hip arthroplasty	orthopaedic surgery	No
CD010349.PUB2	Iodine-131-meta-iodobenzylguanidine therapy for patients with newly diagnosed high-risk neuroblastoma	neurosurgery	No
CD010712	Nonoperative treatment for lumbar spinal stenosis with neurogenic claudication	orthopaedic surgery	No
CD011478.PUB2	Type II or type III radical hysterectomy compared to chemoradiotherapy as a primary intervention for stage IB2 cervical cancer	obstetrics and gynecology	No
CD002116.PUB2	Drug treatment for faecal incontinence in adults	general surgery	No
CD005029.PUB2	Treatment for ataxia in multiple sclerosis	neurosurgery	No
CD008107.PUB2	Perioperative chemo(radio)therapy versus primary surgery for resectable adenocarcinoma of the stomach, gastroesophageal junction, and lower esophagus	general surgery	No
CD008602.PUB4	Interventions for congenital talipes equinovarus (clubfoot)	orthopaedic surgery	No
CD004461.PUB3	Interventions for recurrent idiopathic epistaxis (nosebleeds) in children	otolaryngology	No
CD006476.PUB3	Management for intussusception in children	general surgery	No
CD009166.PUB2	Cervical stitch (cerclage) for preventing preterm birth in multiple pregnancy	obstetrics and gynecology	No
CD002221.PUB2	Interventions for involutional lower lid entropion	ophthalmology	No
CD009379.PUB2	Amniotic membrane transplantation for acute ocular burns	ophthalmology	Yes
CD003296.PUB3	Retinoids for preventing the progression of cervical intra-epithelial neoplasia	obstetrics and gynecology	No
CD004917.PUB3	Interventions for infantile esotropia	ophthalmology	No
CD003431.PUB3	Non surgical therapy for anal fissure	general surgery	Yes
CD007340.PUB2	Bariatric surgery for non-alcoholic steatohepatitis in obese patients	general surgery	No

For peer review only - http://bmjopen.bmj.com/site/about/guidelines.xhtml

For peer review only

CDSR_ID	Title	Specialty	Comparison available
CD001122.PUB5	Laparoscopic ovarian drilling for ovulation induction in women with anovulatory polycystic ovary syndrome	obstetrics and gynecology	Yes
CD007156.PUB2	Interventions for the management of oral submucous fibrosis	otolaryngology	No
CD012802.PUB2	Ab interno supraciliary microstent surgery for open-angle glaucoma	ophthalmology	No
CD004399.PUB3	Medical versus surgical interventions for open angle glaucoma	ophthalmology	No
CD009266.PUB2	Non-steroidal antiandrogen monotherapy compared with luteinising hormone-releasing hormone agonists or surgical castration monotherapy for advanced prostate cancer	urology	No
CD010273.PUB2	Interventions for treating postpartum constipation	general surgery	No
CD009366.PUB2	Lumbar sympathectomy versus prostanoids for critical limb ischaemia due to non-reconstructable peripheral arterial disease	orthopaedic surgery	Yes
CD007060.PUB2	Liver resection versus other treatments for neuroendocrine tumours in patients with resectable liver metastases	general surgery	No
CD008088.PUB3	Anti-TNF-Œ± treatment for pelvic pain associated with endometriosis	obstetrics and gynecology	No
CD004982.PUB6	Treatment for superficial thrombophlebitis of the leg	vascular surgery	Yes
CD007939.PUB2	Single herbal medicine for diabetic retinopathy	ophthalmology	No
CD002000.PUB3	Bypass surgery for chronic lower limb ischaemia	vascular surgery	No
CD012017.PUB2	Grommets (ventilation tubes) for recurrent acute otitis media in children	otolaryngology	Yes
CD009968.PUB2	Botulinum toxin for upper oesophageal sphincter dysfunction in neurological swallowing disorders	general surgery	No
CD004272.PUB3	Surgery versus primary endocrine therapy for operable primary breast cancer in elderly women (70 years plus)	general surgery	Yes
CD007118.PUB2	Palliative cytoreductive surgery versus other palliative treatments in patients with unresectable liver metastases from gastro-entero-pancreatic neuroendocrine tumours	general surgery	No
CD006714.PUB2	Surgical versus medical methods for second trimester induced abortion	obstetrics and gynecology	Yes
CD011174.PUB2	Interventions for non-tubal ectopic pregnancy	obstetrics and gynecology	No
CD010541.PUB3	Surgery for epilepsy	neurosurgery	Yes
CD013034.PUB2	Surgery for patellar tendinopathy (jumper's knee)	orthopaedic surgery	Yes
CD007481.PUB3	Chemical pleurodesis versus surgical intervention for persistent and recurrent pneumothoraces in cystic fibrosis	thoracic surgery	No
CD003712.PUB3	Transmyocardial laser revascularization versus medical therapy for refractory angina	cardiac surgery	Yes
CD008997.PUB2	Non-resection versus resection for an asymptomatic primary tumour in patients with unresectable Stage IV colorectal cancer	general surgery	No
CD005081.PUB3	Medical and surgical treatment for ocular myasthenia	ophthalmology	No
CD013099.PUB2	Interventions for bacterial folliculitis and boils (furuncles and carbuncles)	general surgery	No
CD011837.PUB2	Medical and surgical interventions for the treatment of usual-type vulval intraepithelial neoplasia	obstetrics and gynecology	No
CD003951.PUB3	Surgical versus medical treatment with cyclooxygenase inhibitors for symptomatic patent ductus arteriosus in preterm infants	cardiac surgery	Yes
CD007261.PUB2	Interventions for managing temporomandibular joint osteoarthritis	orthopaedic surgery	No
CD003193.PUB4	Anticholinergic drugs versus non-drug active therapies for non-neurogenic overactive bladder syndrome in adults	urology	No
CD009493.PUB2	N-acetylcarnosine (NAC) drops for age-related cataract	ophthalmology	No
CD005198.PUB3	Therapeutic interventions for Burkitt lymphoma in children	otolaryngology	No
CD004981.PUB4	Treatment for femoral pseudoaneurysms	vascular surgery	No
CD003525.PUB2	Surgery for lateral elbow pain	orthopaedic surgery	No
CD013006.PUB2	Interventions for the management of obesity in people with bipolar disorder	general surgery	No
CD013404.PUB2	Surgical interventions for treating intracapsular hip fractures in older adults: a network meta-analysis	orthopaedic surgery	No
CD011725.PUB2	Indomethacin for intracranial hypertension secondary to severe traumatic brain injury in adults	neurosurgery	No

For peer review only - http://bmjopen.bmj.com/site/about/guidelines.xhtml

CDSR_ID	Title	Specialty	Comparison available
CD009526.PUB2	Ovarian surgery for symptom relief in women with polycystic ovary syndrome	obstetrics and gynecology	Yes
CD003855.PUB3	Surgery versus medical therapy for heavy menstrual bleeding	obstetrics and gynecology	Yes
CD009505.PUB2	Aromatase inhibitors for uterine fibroids	obstetrics and gynecology	No
CD003037.PUB2	Medical versus surgical methods for first trimester termination of pregnancy	obstetrics and gynecology	Yes
CD011169.PUB2	Selective oestrogen receptor modulators (SERMs) for endometriosis	obstetrics and gynecology	No
CD007924.PUB3	Medical interventions for high-grade vulval intraepithelial neoplasia	obstetrics and gynecology	No
CD008111.PUB2	Thymectomy for non-thymomatous myasthenia gravis	thoracic surgery	No
CD007223.PUB4	Medical treatments for incomplete miscarriage	obstetrics and gynecology	Yes
CD010308.PUB2	Interventions for melanoma in situ, including lentigo maligna	general surgery	No
CD007468.PUB4	Surgical interventions for the early management of Bell's palsy	neurosurgery	No
CD007792.PUB2	Palliative surgery versus medical management for bowel obstruction in ovarian cancer	general surgery	No
CD008455.PUB2	Interventions for treating bisphosphonate-related osteonecrosis of the jaw (BRONJ)	orthopaedic surgery	No
CD002115.PUB5	Management of faecal incontinence and constipation in adults with central neurological diseases	general surgery	No
CD006991.PUB2	Surgical versus medical interventions for chronic rhinosinusitis with nasal polyps	otolaryngology	No
CD001496.PUB2	Pharmacological and surgical interventions for the treatment of gastro-oesophageal reflux in adults and children with asthma	general surgery	No
CD008571.PUB2	Interventions for women with endometrioma prior to assisted reproductive technology	obstetrics and gynecology	No
CD006544.PUB3	Prostanoids for critical limb ischaemia	vascular surgery	No
CD003435.PUB2	Surgical decompression for cerebral oedema in acute ischaemic stroke	neurosurgery	Yes
CD013325.PUB2	Interventions for treating people with symptoms of bladder pain syndrome: a network meta-analysis	urology	No
CD001066.PUB3	Interventions for varicose veins and leg oedema in pregnancy	vascular surgery	No
CD006388.PUB2	Octreotide for the treatment of chylothorax in neonates	thoracic surgery	No
CD003658.PUB3	Needling for encapsulated trabeculectomy filtering blebs	ophthalmology	No
CD006152.PUB2	Decompressive surgery of lower limbs for symmetrical diabetic peripheral neuropathy	orthopaedic surgery	No
CD001896.PUB2	Surgical interruption of pelvic nerve pathways for primary and secondary dysmenorrhoea	obstetrics and gynecology	No
CD004699.PUB2	Surgery for local and locally advanced non-small cell lung cancer	thoracic surgery	No
CD002867	Treatments for secondary postpartum haemorrhage	obstetrics and gynecology	No
CD006373.PUB2	Interventions for treating functional dysphonia in adults	otolaryngology	No
CD001541.PUB3	Interventions for ingrowing toenails	general surgery	No
CD013469.PUB2	Surgical and medical interventions for abdominal aortic graft infections	vascular surgery	No
CD001219	Corticosteroids for the resolution of malignant bowel obstruction in advanced gynaecological and gastrointestinal cancer	general surgery	No
CD005304.PUB3	Interventions for primary (intrinsic) tracheomalacia in children	thoracic surgery	No
CD011498.PUB2	Non-surgical versus surgical treatment for oesophageal cancer	general surgery	Yes
CD002784.PUB3	Surgery versus thrombolysis for initial management of acute limb ischaemia	vascular surgery	Yes
CD006499.PUB4	Botulinum toxin for the treatment of strabismus	ophthalmology	Yes
CD005024.PUB3	Surgery for traumatic optic neuropathy	general surgery	No
CD003243.PUB3	Laparoscopic fundoplication surgery versus medical management for gastro-oesophageal reflux disease (GORD) in adults	general surgery	Yes
CD003118.PUB2	Interventions for the treatment of Morton's neuroma	orthopaedic surgery	No
CD001001.PUB3	Lung volume reduction surgery for diffuse emphysema	thoracic surgery	No
CD010784.PUB3	Medical and surgical interventions for the treatment of urinary stones in children	urology	No

For peer review only - http://bmjopen.bmj.com/site/about/guidelines.xhtml

CDSR_ID	Title	Specialty	Comparison available
CD000324.PUB2	Interventions for tubal ectopic pregnancy	obstetrics and gynecology	No
CD000526.PUB2	Interventions for treating tuberculous pericarditis	cardiac surgery	No
CD004156.PUB4	Treatment for spasticity in amyotrophic lateral sclerosis/motor neuron disease	neurosurgery	No
CD004159.PUB3	Treatment for meralgia paraesthetica	neurosurgery	No
CD006797.PUB2	Surgical resection versus non-surgical treatment for hepatic node positive patients with colorectal liver metastases	general surgery	No
CD007510.PUB3	Botulinum toxin for masseter hypertrophy	otolaryngology	No
CD011523.PUB2	Medical versus surgical treatment for refractory or recurrent peptic ulcer	general surgery	No
CD001802.PUB3	Tonsillectomy or adenotonsillectomy versus non-surgical treatment for chronic/recurrent acute tonsillitis	otolaryngology	Yes
CD007383.PUB3	Surgical versus non-surgical management of abdominal injury	general surgery	No
CD006981.PUB2	Treatment for sialorrhea (excessive saliva) in people with motor neuron disease/amyotrophic lateral sclerosis	otolaryngology	No
CD001829.PUB4	Interventions for treating oral leukoplakia to prevent oral cancer	otolaryngology	No
CD001934.PUB2	Surgical versus non-surgical interventions for vocal cord nodules	otolaryngology	No
CD003412.PUB3	Interventions for basal cell carcinoma of the skin	dermatology	Yes
CD003425.PUB4	Splenectomy versus conservative management for acute sequestration crises in people with sickle cell disease	general surgery	No
CD003983.PUB3	Decompressive craniectomy for the treatment of high intracranial pressure in closed traumatic brain injury	neurosurgery	Yes
CD004098.PUB2	Levothyroxine or minimally invasive therapies for benign thyroid nodules	general surgery	No
CD004437.PUB6	Thrombolytic therapy for pulmonary embolism	cardiac surgery	No
CD004927.PUB4	Surgical management of functional bladder outlet obstruction in adults with neurogenic bladder dysfunction	urology	No
CD005619.PUB3	Subacromial decompression surgery for rotator cuff disease	orthopaedic surgery	No
CD006032.PUB4	Steroids for traumatic optic neuropathy	ophthalmology	No
CD006746.PUB4	Laser peripheral iridoplasty for chronic angle closure	ophthalmology	No
CD007281.PUB2	Interventions for cutaneous Bowen's disease	dermatology	No
CD007404.PUB2	Interventions for central giant cell granuloma (CGCG) of the jaws	otolaryngology	No
CD007535.PUB4	Chinese herbal medicine for subfertile women with polycystic ovarian syndrome	obstetrics and gynecology	No
CD008280.PUB2	Interventions for atrophic rhinitis	otolaryngology	No
CD009244.PUB2	Interventions for anal canal intraepithelial neoplasia	general surgery	No
CD010287.PUB3	Aromatase inhibitors (letrozole) for subfertile women with polycystic ovary syndrome	obstetrics and gynecology	Yes
CD010651.PUB2	Surgical versus non-surgical management for pleural empyema	thoracic surgery	Yes
CD011160.PUB2	Anti-vascular endothelial growth factor for choroidal neovascularisation in people with pathological myopia	ophthalmology	Yes
CD012742.PUB2	Subconjunctival draining minimally-invasive glaucoma devices for medically uncontrolled glaucoma	ophthalmology	No
CD012743.PUB2	Ab interno trabecular bypass surgery with iStent for open-angle glaucoma	ophthalmology	Yes
CD012834.PUB2	Medical and surgical abortion for women living with HIV	obstetrics and gynecology	No
CD012879.PUB2	Shoulder replacement surgery for osteoarthritis and rotator cuff tear arthropathy	orthopaedic surgery	No
CD006131.PUB3	Interventions for Mooren's ulcer	dermatology	No
CD007677.PUB4	Pentoxifylline for the treatment of endometriosis-associated pain and infertility	obstetrics and gynecology	No
CD012740.PUB2	Ab interno trabecular bypass surgery with Schlemm´s canal microstent (Hydrus) for open angle glaucoma	ophthalmology	No
CD006151.PUB3	Fundoplication versus postoperative medication for gastro-oesophageal reflux in children with neurological impairment undergoing gastrostomy	general surgery	No
CD010081.PUB2	Interventions for hidradenitis suppurativa	dermatology	No
CD007630.PUB2	Surgical orbital decompression for thyroid eye disease	otolaryngology	Yes
CD011165.PUB2	Tonsillectomy or adenotonsillectomy versus non-surgical management for obstructive sleep-disordered breathing in children	otolaryngology	No
CD005656.PUB3	Intravitreal steroids for macular edema in diabetes	ophthalmology	No

For peer review only - http://bmjopen.bmj.com/site/about/guidelines.xhtml

CDSR_ID	Title	Specialty	Comparison available
CD009860.PUB2	Surgery for trigger finger	orthopaedic surgery	Yes
CD013502	Surgery for rotator cuff tears	orthopaedic surgery	Yes
CD002180	Surgery versus non-surgical treatment for bronchiectasis	thoracic surgery	No
CD010868.PUB2	Interventions for dissociated vertical deviation	ophthalmology	No
CD001408.PUB2	Botulinum toxin type A in the treatment of lower limb spasticity in children with cerebral palsy	orthopaedic surgery	No
CD003919.PUB2	Laser trabeculoplasty for open angle glaucoma	ophthalmology	Yes
CD010312.PUB2	Prostaglandins for management of retained placenta	obstetrics and gynecology	No
CD011693.PUB3	Ab interno trabecular bypass surgery with Trabectome for open-angle glaucoma	ophthalmology	No
CD008669.PUB3	Tonsillectomy for periodic fever, aphthous stomatitis, pharyngitis and cervical adenitis syndrome (PFAPA)	otolaryngology	No
CD008128.PUB2	Treatment of valvular heart disease during pregnancy for improving maternal and neonatal outcome	cardiac surgery	No
CD001923.PUB2	Carotid endarterectomy for asymptomatic carotid stenosis	vascular surgery	Yes
CD010960.PUB2	Injection therapies for Achilles tendinopathy	orthopaedic surgery	No
CD003738.PUB3	Interventions for preventing posterior capsule opacification	ophthalmology	No
CD013000.PUB2	Interventions for orbital lymphangioma	otolaryngology	No
CD008282	Adenoidectomy for recurrent or chronic nasal symptoms in children	otolaryngology	No
CD003263.PUB5	Interventions for vitiligo	dermatology	No
CD008583.PUB3	Ultrasound-guided transvaginal ovarian needle drilling for clomiphene-resistant polycystic ovarian syndrome in subfertile women	obstetrics and gynecology	No
CD007810.PUB2	Adenoidectomy for otitis media in children	otolaryngology	No
CD006181.PUB2	Prophylactic surgical ligation of patent ductus arteriosus for prevention of mortality and morbidity in extremely low birth weight infants	cardiac surgery	No
CD011917.PUB2	Surgery for limited-stage small-cell lung cancer	thoracic surgery	No
CD010264.PUB2	Surgical versus non-surgical treatment for lumbar spinal stenosis	orthopaedic surgery	Yes
CD008732.PUB2	Macular grid laser photocoagulation for branch retinal vein occlusion	ophthalmology	No
CD011680.PUB2	Interventions for necrotizing soft tissue infections in adults	general surgery	No
CD001801.PUB3	Grommets (ventilation tubes) for hearing loss associated with otitis media with effusion in children	otolaryngology	No
CD006205.PUB4	Interventions for the treatment of oral and oropharyngeal cancers: surgical treatment	otolaryngology	No
CD009245.PUB3	Interventions for the treatment of Paget's disease of the vulva	obstetrics and gynecology	No
CD012798.PUB3	Interventions for treating distal intestinal obstruction syndrome (DIOS) in cystic fibrosis	general surgery	No
CD008089.PUB2	Surgery for shoulder osteoarthritis	orthopaedic surgery	No
CD008497.PUB3	Deep brain and cortical stimulation for epilepsy	neurosurgery	No
CD004325.PUB2	Surgical versus non-surgical treatment for acute anterior shoulder dislocation	orthopaedic surgery	No
CD005048.PUB4	Interventions for dysphagia in oesophageal cancer	general surgery	No
CD000200.PUB2	Surgery for primary supratentorial intracerebral haemorrhage	neurosurgery	Yes
CD011031.PUB3	Laparoscopic surgery for endometriosis	obstetrics and gynecology	No
CD010796.PUB2	Surgery for treating hip impingement (femoroacetabular impingement)	orthopaedic surgery	No
CD006769.PUB2	Interventions for late trabeculectomy bleb leak	ophthalmology	No
CD001532.PUB5	Interventions for primary vesicoureteric reflux	urology	Yes
CD008104.PUB2	Interventions for treating osteochondral defects of the talus in adults	orthopaedic surgery	No
CD001552.PUB2	Surgical versus non-surgical treatment for carpal tunnel syndrome	orthopaedic surgery	Yes

For peer review only - http://bmjopen.bmj.com/site/about/guidelines.xhtml

Supplementary Figures and Tables

Supplementary table 1. Reviews per specialty

Specialty	Total reviews	Reviews with at least one comparison (%)
Cardiac surgery	6	2 (33)
Dermatology	5	1 (20)
General surgery	35	5 (14)
Neurosurgery	12	5 (42)
Obstetrics and gynecology	31	8 (26)
Ophthalmology	25	5 (20)
Orthopaedic surgery	23	6 (26)
Otolaryngology	23	3 (13)
Thoracic surgery	9	1 (11)
Urology	7	1 (14)
Vascular surgery	12	4 (33)

For peer review only

Supplementary Table 2. Inconclusive comparisons between surgery and drugs

Surgical arm	Drug arm	Disease	Outcome	Treatment effect (95% CI)	GRADE assessment
			Cardiac surgery		
Transmyocardial lazer revascularization	Continued medication	Refractory angina	Overall mortality	OR=1.12 (0.77-1.63)	High
			Postoperative mortality (30 d)	OR=1.19 (0.63-2.24)	High
Surgical closure	IV indomethacin	Patent ductus arteriosus	Death before discharge	RR=0.67 (0.34-1.31)	
			Dermatology		
Surgical excision	Imiquimod	BCC	Patient-rated good/excellent cosmetic outcome	RR=1 (0.94-1.06)	Low
			General surgery		
Surgery	Tamoxifen	Primary breast cancer	Overall survival	HR=0.98 (0.81-1.2)	Low
Laparoscopic fundoplication	Protein pump inhibitors	GERD	Health-related quality of life (<1 y)	SMD=0.14 (-0.02-0.3)+	Very Low
			Health-related QOL (1-5 y)	SMD=0.03 (-0.19-0.24)+	Very Low
			GORD-specific quality of life (1-5 y)	SMD=0.28 (-0.27-0.84)+	Very Low
Oesophagectomy	Chemoradiotherapy and/or radiotherapy	Oesophageal cancer	Short-term mortality	RR=0.39 (0.11-1.35)	Very Low
			Long-term mortality	RR=1.03 (0.92-1.14)	Low
			Medium-term health-related QOL	MD=-0.95 (-2.1-0.2)	Very Low
			Neurosurgery		
Decompressive surgery	Prednisolone	Leprosy	Change in sensory score after one year	MD=0.08 (-2.45-2.61)	Very Low
			Proportion of ulnar nerves with sensory improvement after one year	RR=1.13 (0.71-1.77)	Very Low
			Change in motor score after one year	MD=0.82 (-1.34-2.98)	Very Low
			Proportion of ulnar nerves with motor improvement after one year	RR=0.91 (0.64-1.28)	Very Low
Decompressive craniectomy	Medical treatment (including barbiturates)	High ICP in closed TBI	Neurological unfavourable outcome 6 mo	RR=1 (0.71-1.4)	Low
			Mortality 6 mo	RR=0.66 (0.43-1.01)	Moderate
			Obstetrics and gynaecology		
Suction aspiration	Vaginal or oral misoprostol	Abortion	Death or serious complication	RR=1 (0.04-25)	

For peer review only

Surgical arm	Drug arm	Disease	Outcome	Treatment effect (95% CI)	GRADE assessment
Suction aspiration	Misoprostol	Abortion	Composite outcome of death or serious complication	RR=1.53 (0.45-5.16)	Very Low
Suction aspiration	Misoprostol and mifepristone	Abortion	Complete miscarriage	RR=1.29 (0.96-1.73)	Very Low
			Composite outcome of death or serious complication	RR=0.14 (0.01-2.74)	Very Low
Suction aspiration	Vaginal suppositories or im inj. of 9-methylene-PGE2	Abortion	Abortion not completeted with intended method	OR=0.62 (0.02-16.6)	
			Ongoing pregnancy	OR=1.82 (0.54-6.25)	
			Pelvic infection	OR=0.46 (0.14-1.56)	
Dilatation and curettage	Misoprostol	Abortion	Composite outcome of death or serious complication	RR=0.79 (0.34-1.85)	Very Low
Laparoscopic ovarian drilling	Metformin, Clomiphene	PCOS	Menstrual regularity at 6 mo.	OR=1.02 (0.64-1.64)	Very Low
Laparoscopic ovarian drilling	Letrozele	PCOS	Menstrual regularity at 6 mo.	OR=1.08 (0.64-1.84)	Very Low
Laparoscopic ovarian drilling	Metformin, Letrozol	PCOS	Menstrual regularity at 6 mo.	OR=0.95 (0.49-1.81)	Very Low
Laparoscopic ovarian drilling	Metformin	PCOS	Menstrual regularity at 6 mo.	OR=1.51 (0.62-3.71)	Moderate
Laparoscopic ovarian drilling	Gonadotropins	PCOS	Improvement in androgenic symptoms 6 mo.	OR=3.02 (0.56-16.33)	Low
Laparoscopic ovarian drilling	Metformin	PCOS	Improvement in androgenic symptoms 6 mo.	OR=1 (0.42-2.37)	Low
Laparoscopic ovarian drilling	Letrozele	Infertility due to PCOS	Live birth	RR=0.72 (0.5-1.05)	Moderate
			Rate of ovarian hyperstimulation syndrome	RD=0 (-0.01-0.01)	High
Transcervical resection of endometrium using rollerball coagulation	Hormone therapy or antifibrinolytic	Heavy menstrual bleeding	Control of bleeding (cure or improvement to acceptable level) 5 y	RR=1.14 (0.97-1.34)	Very Low
			Overall satisfaction with treatment 5 y	RR=1.13 (0.94-1.37)	Very Low
			Ophthalmology		
Amniotic membrane transplantation and medication	Lubrication, Antibiotics and Pressure lowering medication	Acute ocular burns	Epithelial defect 21 d post-injury	RR=0.71 (0.27-1.85)	Low
Argon laser trabeculoplasty	IOP reducing medication	Open angle glaucoma	Visual field progression	RR=0.7 (0.42-1.16)	
			Optic neuropathy progression	RR=0.71 (0.38-1.34)	

For peer review only

Surgical arm	Drug arm	Disease	Outcome	Treatment effect (95% CI)	GRADE assessment
Laser surgery	intravitreal anti-VEGF	Pathological myopia	Proportion of participants with a gain of 3+ lines in BCVA at 1 y	RR=0.32 (0.08-1.33)	Low
Surgical correction	Botulinum toxin	Strabismus	Improved ocular alignment > 10 dioptres, children	RR=1.1 (0.86-1.41)	Low
		Orthopaedic surgery			
Arthroscopic surgery	Sclerosing injection	Jumper's knee	Withdrawal rate	OR=1 (0.06-16.89)	Very Low
Open surgery	Corticosteroid injection	Trigger finger	Resolution of triggering	RR=1.48 (0.79-2.76)	Very low
Open section of the carpal ligament	NSAID and splinting or corticosteroid injections	Carpal tunnel syndrome	Improvement in clinical symptoms at three months of follow-up	RR=1.09 (0.91-1.32)	
Surgical rotator cuff repair	Non-operative treatment including corticosteroid injection and exercise	Rotator cuff tear	Pain (VAS) 12 mo	MD=-0.49 (-1.02-0.05)	Moderate
		Otolaryngology			
Surgical orbital decompression	IV Methylprednisolone 1x3 followed by oral prednisolone	Thyroid eye disease	Proportion of successes compared to the proportion of treatment failures as defined by the study authors based on the use of composite outcome scores	RR=0.16 (0.01-1.98)	
		Thoracic surgery			
Open thoracotomy	Thoracostomy drainage (with fibrinolytics)	Pleural empyema	Mortality	RR=NA (NA-NA)	Moderate
VATS	Thoracostomy drainage (with fibrinolytics)	Pleural empyema	Mortality	RR=0.8 (0.04-14.89)	Low
		Urology			
Surgical reimplantation of ureters	Antibiotics	Primary vesicoureteric reflux	Rate of patients with symptomatic UTI	RR=0.95 (0.67-1.35)	
		Vascular surgery			
Surgery including primary amputation	Thrombolysis (w/ rt-Pa or urokinase)	Acute limb ischaemia	Limb salvage (30 d)	OR=0.89 (0.27-2.91)	Low
Saphenofemoral disconnection	Therapeutic LMWH	Superficial thrombophlebitis	Symptomatic VTE	RR=5 (0.25-100)	
			Major bleeding	RR=NA	
Aspirin and carotid surgery	Aspirin	Carotid stenosis	Ipsilateral ischaemic stroke, and any operative stroke or death near occlusion	RR=0.89 (0.6-1.32)	Moderate

Abbreviations
RR: risk ratio
OR: odds ratio

For peer review only - http://bmjopen.bmj.com/site/about/guidelines.xhtml

HR: hazard ratio
MD: mean difference
SMD: standardized mean difference

BCC: basal cell carcinoma of the skin
GERD: Gastro-oesophageal reflux disease
GTN: glyceryl tri-nitrate
IOP: intra-ocular pressure
PCOS: polycystic ovarian syndrome
QOL: Quality of life

For peer review only

For peer review only - http://bmjopen.bmj.com/site/about/guidelines.xhtml

PRISMA 2020 Checklist

Section and Topic	Item #	Checklist item	Location where item is reported
**TITLE**			
Title	1	Identify the report as a systematic review.	1
**ABSTRACT**			
Abstract	2	See the PRISMA 2020 for Abstracts checklist.	4
**INTRODUCTION**			
Rationale	3	Describe the rationale for the review in the context of existing knowledge.	6
Objectives	4	Provide an explicit statement of the objective(s) or question(s) the review addresses.	4
**METHODS**			
Eligibility criteria	5	Specify the inclusion and exclusion criteria for the review and how studies were grouped for the syntheses.	7
Information sources	6	Specify all databases, registers, websites, organisations, reference lists and other sources searched or consulted to identify studies. Specify the date when each source was last searched or consulted.	7
Search strategy	7	Present the full search strategies for all databases, registers and websites, including any filters and limits used.	7
Selection process	8	Specify the methods used to decide whether a study met the inclusion criteria of the review, including how many reviewers screened each record and each report retrieved, whether they worked independently, and if applicable, details of automation tools used in the process.	7
Data collection process	9	Specify the methods used to collect data from reports, including how many reviewers collected data from each report, whether they worked independently, any processes for obtaining or confirming data from study investigators, and if applicable, details of automation tools used in the process.	8
Data items	10a	List and define all outcomes for which data were sought. Specify whether all results that were compatible with each outcome domain in each study were sought (e.g. for all measures, time points, analyses), and if not, the methods used to decide which results to collect.	8
	10b	List and define all other variables for which data were sought (e.g. participant and intervention characteristics, funding sources). Describe any assumptions made about any missing or unclear information.	8
Study risk of bias assessment	11	Specify the methods used to assess risk of bias in the included studies, including details of the tool(s) used, how many reviewers assessed each study and whether they worked independently, and if applicable, details of automation tools used in the process.	8
Effect measures	12	Specify for each outcome the effect measure(s) (e.g. risk ratio, mean difference) used in the synthesis or presentation of results.	8
Synthesis methods	13a	Describe the processes used to decide which studies were eligible for each synthesis (e.g. tabulating the study intervention characteristics and comparing against the planned groups for each synthesis (item #5)).	NA
	13b	Describe any methods required to prepare the data for presentation or synthesis, such as handling of missing summary statistics, or data conversions.	7-8
	13c	Describe any methods used to tabulate or visually display results of individual studies and syntheses.	7
	13d	Describe any methods used to synthesize results and provide a rationale for the choice(s). If meta-analysis was performed, describe the model(s), method(s) to identify the presence and extent of statistical heterogeneity, and software package(s) used.	8
	13e	Describe any methods used to explore possible causes of heterogeneity among study results (e.g. subgroup analysis, meta-regression).	Not relevant
	13f	Describe any sensitivity analyses conducted to assess robustness of the synthesized results.	Not relevant
Reporting bias assessment	14	Describe any methods used to assess risk of bias due to missing results in a synthesis (arising from reporting biases).	
Certainty	15	Describe any methods used to assess certainty (or confidence) in the body of evidence for an outcome.	7-8

For peer review only

PRISMA 2020 Checklist

Section and Topic	Item #	Checklist item	Location where item is reported
assessment			
**RESULTS**			
Study selection	16a	Describe the results of the search and selection process, from the number of records identified in the search to the number of studies included in the review, ideally using a flow diagram.	11
	16b	Cite studies that might appear to meet the inclusion criteria, but which were excluded, and explain why they were excluded.	11
Study characteristics	17	Cite each included study and present its characteristics.	Supplement 1
Risk of bias in studies	18	Present assessments of risk of bias for each included study.	11 (GRADE)
Results of individual studies	19	For all outcomes, present, for each study: (a) summary statistics for each group (where appropriate) and (b) an effect estimate and its precision (e.g. confidence/credible interval), ideally using structured tables or plots.	12-13
Results of syntheses	20a	For each synthesis, briefly summarise the characteristics and risk of bias among contributing studies.	11
	20b	Present results of all statistical syntheses conducted. If meta-analysis was done, present for each the summary estimate and its precision (e.g. confidence/credible interval) and measures of statistical heterogeneity. If comparing groups, describe the direction of the effect.	p. 13 Table 2 & 3
	20c	Present results of all investigations of possible causes of heterogeneity among study results.	Not relevant
	20d	Present results of all sensitivity analyses conducted to assess the robustness of the synthesized results.	Not relevant
Reporting biases	21	Present assessments of risk of bias due to missing results (arising from reporting biases) for each synthesis assessed.	Not relevant
Certainty of evidence	22	Present assessments of certainty (or confidence) in the body of evidence for each outcome assessed.	13
**DISCUSSION**			
Discussion	23a	Provide a general interpretation of the results in the context of other evidence.	15
	23b	Discuss any limitations of the evidence included in the review.	16
	23c	Discuss any limitations of the review processes used.	16
	23d	Discuss implications of the results for practice, policy, and future research.	17
**OTHER INFORMATION**			
Registration and protocol	24a	Provide registration information for the review, including register name and registration number, or state that the review was not registered.	3
	24b	Indicate where the review protocol can be accessed, or state that a protocol was not prepared.	3
	24c	Describe and explain any amendments to information provided at registration or in the protocol.	9
Support	25	Describe sources of financial or non-financial support for the review, and the role of the funders or sponsors in the review.	2
Competing interests	26	Declare any competing interests of review authors.	2
Availability of data, code and other materials	27	Report which of the following are publicly available and where they can be found: template data collection forms; data extracted from included studies; data used for all analyses; analytic code; any other materials used in the review.	2

For peer review only

For peer review only - http://bmjopen.bmj.com/site/about/guidelines.xhtml

From: Page MJ, McKenzie JE, Bossuyt PM, Boutron I, Hoffmann TC, Mulrow CD, et al. The PRISMA 2020 statement: an updated guideline for reporting systematic reviews. BMJ 2021;372:n71. doi:

PRISMA 2020 Checklist

10.1136/bmj.n71

For more information, visit: http://www.prisma-statement.org/

For peer review only

PRISMA 2020 for Abstracts Checklist

Section and Topic	Item #	Checklist item	Reported (Yes/No)
**TITLE**			
Title	1	Identify the report as a systematic review.	Yes
**BACKGROUND**			
Objectives	2	Provide an explicit statement of the main objective(s) or question(s) the review addresses.	Yes
**METHODS**			
Eligibility criteria	3	Specify the inclusion and exclusion criteria for the review.	Yes
Information sources	4	Specify the information sources (e.g. databases, registers) used to identify studies and the date when each was last searched.	Yes
Risk of bias	5	Specify the methods used to assess risk of bias in the included studies.	Yes
Synthesis of results	6	Specify the methods used to present and synthesise results.	Yes
**RESULTS**			
Included studies	7	Give the total number of included studies and participants and summarise relevant characteristics of studies.	Yes
Synthesis of results	8	Present results for main outcomes, preferably indicating the number of included studies and participants for each. If meta-analysis was done, report the summary estimate and confidence/credible interval. If comparing groups, indicate the direction of the effect (i.e. which group is favoured).	Yes
**DISCUSSION**			
Limitations of evidence	9	Provide a brief summary of the limitations of the evidence included in the review (e.g. study risk of bias, inconsistency and imprecision).	No
Interpretation	10	Provide a general interpretation of the results and important implications.	Yes
**OTHER**			

Funding	11	Specify the primary source of funding for the review.	Yes
Registration	12	Provide the register name and registration number.	Yes

From: Page MJ, McKenzie JE, Bossuyt PM, Boutron I, Hoffmann TC, Mulrow CD, et al. The PRISMA 2020 statement: an updated guideline for reporting systematic reviews. BMJ 2021;372:n71. doi: 10.1136/bmj.n71

For more information, visit: http://www.prisma-statement.org/

For peer review only

BMJ Open

Availability of evidence and comparative effectiveness for surgical versus drug interventions: an overview of systematic reviews and meta-analyses

Journal:	*BMJ Open*
Manuscript ID	bmjopen-2023-076675.R1
Article Type:	Original research
Date Submitted by the Author:	19-Nov-2023
Complete List of Authors:	Zavalis, Emmanuel A.; Karolinska Institutet, Department of Learning Informatics Management and Ethics; Stanford University, Meta-Research Innovation Center at Stanford (METRICS)<br>Rameau, Anaïs; Weill Cornell Medical College, Sean Parker Institute for the Voice, Department of Otolaryngology–Head and Neck Surgery<br>Saraswathula, Anirudh; The Johns Hopkins University School of Medicine, Department of Otolaryngology–Head and Neck Surgery<br>Vist, Joachim; Karolinska Institutet, Department of Learning Informatics Management and Ethics<br>Schuit, Ewoud; Utrecht University, Julius Center for Health Sciences and Primary Care, University Medical Center Utrecht; Utrecht University, Cochrane Denmark, University Medical Center Utrecht<br>Ioannidis, John; Stanford University, Stanford Prevention Research Center, Department of Medicine, and Department of Epidemiology and Population Health; Stanford University, Meta-Research Innovation Center at Stanford (METRICS)
**Primary Subject Heading**:	Evidence based practice
Secondary Subject Heading:	Surgery, Medical management
Keywords:	Decision Making, Systematic Review, SURGERY

SCHOLARONE™
Manuscripts

I, the Submitting Author has the right to grant and does grant on behalf of all authors of the Work (as defined in the below author licence), an exclusive licence and/or a non-exclusive licence for contributions from authors who are: i) UK Crown employees; ii) where BMJ has agreed a CC-BY licence shall apply, and/or iii) in accordance with the terms applicable for US Federal Government officers or employees acting as part of their official duties; on a worldwide, perpetual, irrevocable, royalty-free basis to BMJ Publishing Group Ltd ("BMJ") its licensees and where the relevant Journal is co-owned by BMJ to the co-owners of the Journal, to publish the Work in this journal and any other BMJ products and to exploit all rights, as set out in our licence.

The Submitting Author accepts and understands that any supply made under these terms is made by BMJ to the Submitting Author unless you are acting as an employee on behalf of your employer or a postgraduate student of an affiliated institution which is paying any applicable article publishing charge ("APC") for Open Access articles. Where the Submitting Author wishes to make the Work available on an Open Access basis (and intends to pay the relevant APC), the terms of reuse of such Open Access shall be governed by a Creative Commons licence – details of these licences and which Creative Commons licence will apply to this Work are set out in our licence referred to above.

Other than as permitted in any relevant BMJ Author's Self Archiving Policies, I confirm this Work has not been accepted for publication elsewhere, is not being considered for publication elsewhere and does not duplicate material already published. I confirm all authors consent to publication of this Work and authorise the granting of this licence.

For peer review only

Availability of evidence and comparative effectiveness for surgical versus drug interventions: an overview of systematic reviews and meta-analyses

Emmanuel A. Zavalis[1,2*], Anaïs Rameau[3*], Anirudh Saraswathula[4*], Joachim Vist[1], Ewoud Schuit[5,6], John P. A. Ioannidis[2,7]

*co-first authors

1 Department of Learning Informatics Management and Ethics, Karolinska Institutet, Stockholm, Sweden

2 Meta-Research Innovation Center at Stanford (METRICS), Stanford University, Stanford, CA, USA

3 Sean Parker Institute for the Voice, Department of Otolaryngology–Head and Neck Surgery, Weill Cornell Medical College, New York, NY, USA

4 Department of Otolaryngology–Head and Neck Surgery, Johns Hopkins University School of Medicine, Baltimore, MD, USA

5 Julius Center for Health Sciences and Primary Care, University Medical Center Utrecht, Utrecht University, Utrecht, the Netherlands

6 Cochrane Netherland, University Medical Center Utrecht, Utrecht University, Utrecht, the Netherlands

7 Stanford Prevention Research Center, Department of Medicine, and Department of Epidemiology and Population Health, Stanford University School of Medicine, Stanford, CA, USA

Corresponding author:
Emmanuel A. Zavalis
E-mail: emmanuel.zavalis@ki.se

Word count: 3688

Publication history: A preprint of the manuscript has been deposited in medRxiv: doi: *https://doi.org/10.1101/2023.01.30.23285207*

For peer review only

Declarations

Ethics approval and consent to participate

Not applicable

Consent for publication

Not applicable

Availability of data and materials

The dataset supporting the conclusions of this article, and the used code is available in the Open Science Framework repository.

Conflict of interest

Anaïs Rameau is a medical advisor for Perceptron Health, Inc.

Funding

The work of John Ioannidis has been funded by an unrestricted gift from Sue and Bob O'Donnell. Anaïs Rameau is supported by a Paul B. Beeson Emerging Leaders Career Development Award in Aging (K76 AG079040) from the National Institute on Aging and by the Bridge2AI award (OT2 OD032720) from the NIH Common Fund. Anirudh Saraswathula was supported by the National Institute on Deafness and Other Communication Disorders training grant 2T32DC000027. Ewoud Schuit gratefully acknowledges financial contribution for his research by the Netherlands Organisation for Scientific Research (project 825.14.001).

Authors' contributions

AR, AS, EAZ and JPAI developed the idea, EAZ and JPAI interpreted the review data, JV and EAZ extracted the data. ES aided in the statistical analysis. All authors reviewed the manuscript and have edited and approved the submission.

Acknowledgements

Not applicable

For peer review only

Abstract

Objectives. To examine the prevalence of comparisons of surgery to drug regimens, the strength of evidence of such comparisons, and whether surgery or the drug intervention was favored.

Design. Systematic review of systematic reviews (umbrella review)

Data sources. Cochrane Database of Systematic Reviews (CDSR).

Eligibility criteria. Systematic reviews attempting to compare surgical to drug interventions.

Data extraction. We extracted whether the review found any randomized controlled trials for eligible comparisons. Individual trial results were extracted directly from the systematic review.

Synthesis. The outcomes of each meta-analysis was re-synthesized into random-effects meta-analyses. Egger's test and excess significance were assessed.

Results. Overall, 188 systematic reviews intended to compare surgery versus drugs. Only 41 included data from at least one RCT (total, 165 RCTs) and covered a total of 103 different outcomes of various comparisons of surgery versus drugs. A GRADE assessment was performed by the Cochrane reviewers for 87 (83%) outcomes in the reviews, indicating the strength of evidence was high in 4 outcomes (4%), moderate in 22 (21%), low in 27 (26%) and very low in 33 (32%). Based on 95% confidence intervals, the surgical intervention was favored in 38/103 (37%), and the drugs were favored in 13/103 (13%) outcomes. Of the outcomes with high GRADE rating, only one showed conclusive superiority in our re-analysis (sphincterotomy was better than medical therapy for anal fissure). Of the 22 outcomes with moderate GRADE rating, 6 (27%) were inconclusive, 14 (64%) were in favor of surgery, and 2 (9%) were in favor of drugs. There was no evidence of excess significance.

For peer review only - http://bmjopen.bmj.com/site/about/guidelines.xhtml

Conclusions. Though the relative merits of surgical versus drug interventions are important to know for many diseases, high strength randomized evidence is rare. More randomized trials comparing surgery to drug interventions are needed.

Protocol registration. *www.doi.org/10.17605/OSF.IO/RK7HU*

For peer review only

For peer review only - http://bmjopen.bmj.com/site/about/guidelines.xhtml

Strengths and limitations of this study

- The Cochrane database offers comprehensive coverage of health interventions with detailed methods sections that are likely to convey the intention to study surgical versus drug interventions even if no such randomized trials are found.
- Journal-published systematic reviews outside of Cochrane were not considered, but these are unlikely to include topics where no eligible randomized trials are found.
- We did not consider endovascular and endoscopic interventions in the surgery group and we did not consider non-pharmaceutical interventions in the control group.
- We did not consider non-randomized observational studies, but these may have additional biases in estimating the outcomes of surgical versus drug interventions.

For peer review only

For peer review only - http://bmjopen.bmj.com/site/about/guidelines.xhtml

Introduction

Many diseases are treated or managed with surgery. Some of them may also be addressed by pharmaceutical interventions and studying the effectiveness of these different interventions is important in optimizing shared decision-making for patients and physicians. However, the amount and certainty of the evidence we hold in healthcare is limited[1], and this situation is likely worse for surgical interventions due to serious challenges in running placebo-controlled or comparative effectiveness trials[2]. Challenges to controlled trials include unique patient anatomy, operator dependent variables such as the skill or experience of the surgeon[3–5], and the difficulty of successful blinding[6]. Due to these challenges, randomized controlled trials (RCTs) in surgery are less common than in non-surgical medical specialties. Although there have been calls to strengthen the quality of the evidence in surgery[2,7,8], these have resulted in relatively few RCTs assessing surgical interventions, particularly in comparison to medical treatments.

A summary of the existing body, mapping the gaps of evidence on surgical versus medical interventions across diseases does not exist in the literature. A synthesis of this existing body of evidence is important to guide evidence-based care and inform decisions in the clinic where surgery and medical management are both reasonable options. We hypothesized that there may be a dearth of randomized evidence comparing surgery versus drugs and that even in topics where such RCTs exist the evidence provided by them might be weak. To find RCTs comparing surgical vs. pharmaceutical interventions, we conducted an umbrella review (an overview of systematic reviews) [9,10] by searching the Cochrane Database of Systematic Reviews for reviews considering comparisons of surgery to drugs. We aimed to examine the prevalence of

For peer review only - http://bmjopen.bmj.com/site/about/guidelines.xhtml

intended comparisons of surgery to drug regimens, how often such comparisons had any RCTs, and, whenever RCTs were available, what was the strength of evidence of such comparisons, and whether surgery or the drug intervention was favored.

Materials and Methods

This systematic review of systematic reviews (umbrella review) was structured based on the guidance provided by Belbasis et al. [10] (for more information on reviews of reviews, see also Cochrane Handbook Chapter V: Overviews of Reviews [11]). For reporting, we adapted the Preferred Reporting Items for Systematic Review and Meta-Analysis (PRISMA) guidelines[12] and the checklists are found as supplements. The protocol for the data collection, and analysis was pre-registered on the Open Science Framework website [13], together with the raw data and code.

Search strategy and selection criteria

We queried the Cochrane Database of Systematic Reviews using the term "surg*" in "Title/Abstract/Keywords" ("surg*(ti;ab;kw)") on April 25, 2022. Inclusion criteria for reviews were search of RCTs comparing a surgical to a drug intervention.

A surgical intervention was defined as a procedural technique aiming to change anatomy to treat or alleviate a pathology or symptom (including dermatological excisions). We excluded endoscopic and endovascular procedures since many of them are performed by medical rather than surgical specialists. A drug intervention was defined as a treatment that utilized a non-

For peer review only - http://bmjopen.bmj.com/site/about/guidelines.xhtml

supplement and non-vitamin, pharmaceutical agent. Dental procedures, radiation treatment, as well as comparisons of surgery vs. no treatment or only placebo were excluded from our study. Cochrane reviews that intended to compare surgical and pharmaceutical interventions were considered even in cases where the review was unsuccessful in finding any such comparisons.

As many surgical procedures also require drug regimens (e.g., pre-operatively or as background treatment), we allowed comparisons where the surgical arm including a drug intervention was compared to a drug intervention as well. Comparisons of surgery to surgery plus drugs were not eligible, as both arms used surgery.

The articles' abstracts were reviewed by EAZ, and JV who coded the reviews independently for eligibility (include, exclude, unsure) first and then sought to reach a consensus among the reviews coded as unsure by either reviewer. If either reviewer included the review, it was included directly. Remaining differences were mediated by JPAI, and a final check of all included studies was performed by JPAI, EAZ and JV.

Main outcomes

The main outcome assessed was the percentage of Cochrane systematic reviews that found eligible RCTs comparing head-to-head surgical and pharmacological interventions among all the reviews aiming to look for such studies. The strength of evidence of the existing comparison was also treated as a main outcome, as were the direction of effects in the review assessments, both in the original Cochrane analysis and our standardized re-analysis.

For peer review only - http://bmjopen.bmj.com/site/about/guidelines.xhtml

Data extraction

EAZ extracted data for the included systematic reviews. The included systematic reviews were further classified into their corresponding surgical specialty field: cardiac surgery, dermatology, general surgery, neurosurgery, obstetrics and gynaecology, ophthalmology, orthopaedic surgery, otolaryngology, plastic surgery, thoracic surgery, urology and vascular surgery.

Whenever data were available from at least one RCT comparing a surgical to a drug arm, we identified the primary outcome(s) of the systematic review for the eligible comparison(s) by examining the methods section of the systematic review, and classified it as either mortality, composite or non-mortality. Data, in the form of contingency tables or means, standard deviations and number of participants in each arms, from individual RCTs were then collected from Cochrane eligible reviews. We also collected available GRADE assessments[14] for the eligible comparisons and outcomes and the summary effect size as well as the 95% confidence interval of the effect for the eligible comparison outcomes. Reviews that found no RCT of drugs to surgery were tabulated as having no data.

Meta-analysis

As Cochrane reviewers may have used different statistical models in each topic to combine the results of RCTs in meta-analyses we aimed for standardization. To achieve it, we recalculated the summary effect size and heterogeneity for each topic using a random effects model following the Hartung-Knapp-Sidik-Jonkman approach[15,16] so that all outcomes/topics would be analyzed with the same statistical methods. The modified Haldane-Anscombe continuity

For peer review only - http://bmjopen.bmj.com/site/about/guidelines.xhtml

correction was used, i.e. when studies had no event in either the surgical or the drug arm we added 0.5 to the entire contingency table of the specific study[17].

The analysis of the data was performed using R version 4.1.3 (2022-03-10)[18], with assessment of statistical significance using a threshold for α of 0.005, as previously proposed[19]. The Wilson approach was used for confidence intervals (99.5%) created for the primary outcomes.

Additions to the protocol

The original pre-registered protocol can be found in *www.doi.org/10.17605/OSF.IO/3QVW9*.

Some additions were made during the process of conducting this umbrella review. For each review, we noted the search date of the reviews to understand how old they may be. We assessed inter-rater reliability using Cohen's κ. We also probed for hints of bias by using the test of excess significance for each topic with 2 or more RCTs (and for the composite of observed and expected statistical significant results across all topics) [20], and small-study effects Egger's regression for meta-analyses with 3 or more RCTs [21].

For each RCT in the included reviews we extracted their year of publication to capture how recent the evidence was. Then, we extracted the specialty orientation of the journal, in which the RCT was published, using the categories "mostly surgical", "general", and "mostly non-surgical". The category "mostly surgical" includes those journals that have "surgery" in their title, those that have the name of a surgical specialty in their title, and those affiliated with a surgical society. The category "general" pertains to journals that cover all of medicine and its

For peer review only - http://bmjopen.bmj.com/site/about/guidelines.xhtml

specialties, surgical and non-surgical. The category “mostly non-surgical” includes all the remaining journals. We assessed whether the direction of effects (favoring surgery or favoring drug) was associated with the type of journal, hypothesizing that RCTs published in mostly surgical journals may be more likely than other journals to favor surgery. We also examined whether the eligible RCTs that were included in the systematic reviews might have any overlap between different reviews. Finally we extracted information on risk of bias assessments of the eligible RCTs, as these assessments had been performed in the Cochrane systematic reviews that had included the RCTs.

Patient and Public Involvement

No patients were involved in the design and conduct of this umbrella review

For peer review only

For peer review only - http://bmjopen.bmj.com/site/about/guidelines.xhtml

Results

Search results

The selection flowchart for Cochrane systematic reviews is represented in Figure 1. The search strategy retrieved 2495 articles from the Cochrane Database of Systematic Reviews. Among them, 440 were excluded by an automated search for withdrawn reviews and of studies with no mention of the word surgery and any of its variations in the abstract. Further manual assessment of titles and abstracts in duplicate resulted in 223 Cochrane reviews being potentially eligible The inter-rater reliability was fair with a κ of 0.36 and 90% agreement on exclusion. All reviewer differences were in the articles classified as "unsure" by either reviewer.

Upon full-text evaluation, 35 were excluded: in 5 reviews, the surgical and drug treatments were not in separate arms and hence they were not an eligible head-to-head comparison[22–26]; in 7 reviews, there was no surgical intervention arm[27–33]; in 17 reviews, there was no drug intervention [34–39,39–49]); 2 reviews were excluded for evaluating an endoscopic intervention [50,51]; 3 reviews were excluded for evaluating an endovascular intervention [52–54]; and finally 1 review was excluded for being an umbrella review[55].

Therefore, 188 Cochrane reviews were found to meet the inclusion criteria (Supplemental Digital Content data file 1). Of those, 147 Cochrane reviews aimed to investigate surgical versus drug interventions but were unable to find any RCTs meeting their selection criteria. The remaining 41 reviews contained data for at least one RCT in at least one head-to-head comparison of a surgical versus a drug intervention arm (22% (99.5% CI 14 to 31%)).

For peer review only - http://bmjopen.bmj.com/site/about/guidelines.xhtml

The 188 reviews covered all major surgical specialties (Supplementary Table 1), with the most commonly represented specialties being general surgery (n=35), obstetrics and gynecology (n=31), ophthalmology (n=25), orthopedic surgery (n=23) and otolaryngology (n=23). When examining whether any specialty had compared surgery to drugs more than others, no significant difference was found (Fisher's exact p=0.62).

Eligible RCTs for surgery versus drug comparisons

The 41 eligible reviews with data included 103 comparisons of surgery versus drug treatments with data on various primary outcomes (Table 1), and they included data from a total of 165 RCTs with a total of 295 primary outcome assessments. For the 165 trials, the median publication year was 2005 and the interquartile range was 1994 to 2016. The median search date year of the eligible reviews was 2016 (interquartile range, 2010 to 2022). 19 of the 165 trials were part of two different Cochrane reviews. 14 of these 19 trials also overlapped in terms of addressing the same outcome and treatment arms. The overlapping studies comprised >50% of the included RCTs in 2 of 103 meta-analyses.

Risk of bias in eligible RCTs

Risk of bias assessments of the 165 eligible RCTs by the authors of the original Cochrane systematic reviews did not always include the same elements. Specifically, for generation of the randomization sequence, information had been extracted in 141 trials and of those 6 (4%) were deemed to be at high risk of bias, 42 (30%) were unclear and 93 (66%) were at low risk of bias. The respective numbers were 9 (6%) high risk, 63 (39%) unclear, and 89 (55%) low risk among 161 RCTs extracted for risk of allocation bias; 101 (73%) high risk, 29 (21%) unclear, and 9

For peer review only - http://bmjopen.bmj.com/site/about/guidelines.xhtml

(6%) low risk among 139 RCTs extracted for performance bias; 47 (34%) high risk, 71 (51%) unclear, and 21 (15%) low risk among 139 RCTs extracted for detection bias; 20 (16%) high risk, 15 (12%) unclear, and 90 (72%) low risk among 125 RCTs extracted for attrition bias; 17 (12%) high risk, 56 (41%) unclear, and 64 (47%) low risk among 137 RCTs extracted for reporting bias. and 17 (13%) high risk, 29 (23%) unclear, and 80 (64%) low risk among 126 extracted for other risk of bias.

Comparative effectiveness of surgery versus drugs

Based on the 95% confidence interval of the summary estimate obtained by the Cochrane review authors, surgery was more effective in 36 of the 103 outcomes of various comparisons (35% (99.5% CI 23 to 49%)), and drugs were more effective in 15 (15% (99.5% CI 6 to 26%)). Fifty-two (50% (99.5 CI% 37 to 64%)) outcomes were inconclusive. The respective numbers were 1/12 (8%), 1/12 (8%), and 10/12 (83%) for mortality outcomes; 3/11 (27%), 3/11 (27%) and 5/11 (46%) for composite outcomes; and 32/80 (40%), 11/80 (14%), and 37/80 (46%) for non-mortality outcomes.

When we standardized the meta-analyses to use the same random effects method for all analyses, surgery was favored in 28/103 outcomes (32%), drugs were favored in 9/103 (10%) outcomes and 66/103 (58%) outcomes were inconclusive. The respective numbers were 1/12 (8%), 0/12 (0%), and 11/12 (92%) for mortality outcomes; 3/11 (18%), 2/11 (27%) and 6/11 (55%) for composite outcomes; and 24/80 (30%) 7/80 (9%), and 49/80 (61%) for non-mortality outcomes.

For peer review only - http://bmjopen.bmj.com/site/about/guidelines.xhtml

Table 2 shows the topics for which the surgical intervention was found to be more effective and Table 3 shows those where the drug arm was found to be more effective, all according to the Cochrane authors' analysis. Supplementary Table 2 does the same for the topics for which the comparisons were inconclusive.

Tests of bias and heterogeneity

Of the 103 comparisons, only 31 had >=3 studies to be able to run an Egger regression for small study effects and only 5 had at least 10 studies to allow a meaningful application of this regression test. 3/5 with 10 or more studies had a small study effects signal suggestive of potential publication bias ($p<0.05$); all 3 compared surgical to pharmacological methods of abortion. The test of excess significance applied to all outcomes with >=2 studies gave signals of potential bias in 16/53 outcomes (245 individual study outcomes) and across all outcomes the expected number of statistically significant results was 74 versus an observed 84 across 245 study outcomes ($p=0.27$). Among the 50 topics with 2 or more studies, the median of I^2 was 43% (IQR, 0%-80%).

Strength of evidence according to GRADE

GRADE assessment of the strength of the evidence showed high rating for 4 outcomes (4%), moderate for 22 (21%), low for 27 (26%), and very low for 33 (32%). No GRADE assessment was performed for 17 (17%) outcomes.

For peer review only - http://bmjopen.bmj.com/site/about/guidelines.xhtml

According to GRADE assessments, only cardiac surgery, obstetrics and gynecology and general surgery interventions had high GRADE ratings. Otolaryngology and dermatology had many moderate ratings. Almost all other GRADE ratings were low or very low (Table 4).

Of the four outcomes with high GRADE rating, sphincterotomy for anal fissure showed superiority over medical treatment while the other three comparisons were inconclusive. Of the 22 outcomes with moderate GRADE rating, 6 (27%) were inconclusive, 14 (64%) were in favor of surgery, and 2 (9%) were in favor of the drug regimen according to the calculations of the Cochrane authors (14 (64%), were inconclusive, 7 (32%) favored the surgical arm and 1 (5%) were in favor of the drug regimen according to our standard random-effects calculations).

Results of RCTs according to journal of publication

Of the 165 eligible RCTs (295 outcome assessments), 73 RCTs (133 assessments) were published in mostly surgical journals, 38 RCTs (69 assessments) in general journals, and 54 RCTs (93 assessments) in mostly non-surgical journals. Based on 95% confidence intervals for the assessments of RCTs published in mostly surgical journals, 40/133 (30%) were in favor of surgery, 14/133 (11%) were in favor of drugs, and 79/133 (59%) were inconclusive. The respective numbers for the assessments of RCTs published in general journals were 27/69 (39%), 5/69 (7%), and 37/69 (53%); and for the assessments of RCTs published in mostly non-surgical journals they were 22/93 (24%), 15/93 (16%), and 56 (60%), respectively. The proportion of RCTs favoring surgery was not significantly higher in mostly surgical journals (30%) compared to other journals (39% and 24% for general and non-surgical journals respectively) (p=0.18 by Fisher's exact test).

For peer review only - http://bmjopen.bmj.com/site/about/guidelines.xhtml

Discussion

Main findings

In a subset of Cochrane reviews that aimed to compare surgery to drugs we found that only 1 in 5 systematic reviews that had shown interest in such comparisons eventually found data from any RCTs for comparisons of the two modes of interventions. Furthermore, the majority of the comparisons where RCTs of surgery versus drugs had inconclusive results, few studies per meta-analytical outcome (30% with 3 or more studies), and also had low or very low strength of the evidence on GRADE assessments, and many trials had high risk of performance and detection bias.

Anal fissure was the only disease in our sample that had high GRADE evidence and a direction of effect indicating that one intervention (sphincterotomy) was more effective. Consequently, in the vast majority of cases where surgical and pharmaceutical interventions are available for treatment, an evidence-based decision in the clinic is difficult. Our secondary *post hoc* analysis of the type of journal where the eligible RCTs were published showed that results published in surgical journals were not necessarily more prone to favor the surgical arm of an RCT over the pharmaceutical arm.

Strengths

This study covers the entire Cochrane database which is considered a high-quality comprehensive collection of systematic reviews. Cochrane reviews tend to address questions typically asked in routine clinical practice and underpin many clinical guideline recommendations, making this sample all the more relevant to everyday practice [56]. Another

For peer review only - http://bmjopen.bmj.com/site/about/guidelines.xhtml

strength of this study is that all surgical specialties were included. This is, therefore, to our knowledge the first project aiming to assess the extent of comparative evidence for surgery versus pharmacotherapy for a diverse spectrum of diseases.

Limitations

Our analysis has several limitations. First, our pre-defined inclusion criteria excluded non-pharmacological medical interventions. Several comparisons may be found in the literature where surgery is compared against non-surgical non-pharmacological medical interventions, such as CPAP or radiotherapy. We also excluded endovascular and endoscopic procedures since they may be performed by surgical and medical specialists. These eligibility choices aimed to achieve some homogeneity in a project that is by definition already very heterogeneous. The use of an algorithm to filter out papers with no mention of the word surgery as well as the search strategy itself may have led to us missing reviews that discuss a particular surgical procedure but never explicitly mention the word surgery but merely the name of the intervention.

Second, we focused exclusively on RCTs, but other types of evidence, e.g., non-randomized controlled trials, or uncontrolled clinical trials may also exist and sometimes their results may be compelling enough to deem a randomized study unnecessary. Such unquestionable superiority in the absence of randomized evidence is however unlikely [57]. Efforts such as IDEAL [8] have laid out much of the groundwork for performing RCTs in surgical research, yet a dearth of RCTs in the surgical realm of research persists to this day.

For peer review only - http://bmjopen.bmj.com/site/about/guidelines.xhtml

Third, only one database (Cochrane Database of Systematic Reviews) was used for this study, and we did not examine non-Cochrane meta-analyses published as journal articles. While the database aims to be all-inclusive, there are still some topics in medical and surgical care that have not been covered by Cochrane reviews.

However, the Cochrane database is more meticulous in describing its methods and it will routinely publish systematic reviews that have found no eligible articles, while this is unlikely in systematic reviews published in traditional journals. Therefore, including systematic reviews from journals may have distorted the picture and also caused a problem of overlapping systematic reviews. Moreover, we did not assess the methodological rigor or reporting quality of the Cochrane systematic reviews[58], as this was not the focus of our study. Cochrane systematic reviews score very highly in standard tools like AMSTAR[59], both because they are very meticulous and also because AMSTAR and AMSTAR-2 were developed with inspiration from the Cochrane Handbook.

Fourth, it is possible that within the same disease, subgroups of patients may be eligible only for medical or only for surgical treatment, or that one or the other approach is much better only for specific subgroups. With the dearth of evidence we found for the overall analysis, identification of such subgroup effects would be unlikely and error-prone.

Context of these findings

Sequestration between different disciplines and specialties[60] may lead to isolation of specialists which use different tools, and this may lead to a lack of comparisons of the treatments

For peer review only - http://bmjopen.bmj.com/site/about/guidelines.xhtml

that each specialty uses. Each specialty may have its own community, journals, meetings, and research agenda, limiting communication between different specialists even though they may be dealing with the same disease from different angles and with different therapeutic sets. This lack of communication may also be due to differences in mentorship and the trend of sub-specialization in medical training separating clinicians and their practices even further [61], or to differing incentive structures.

Prior literature comparing surgical and medical interventions has assessed specific treatments, such as that for basal cell carcinoma[60], and demonstrated that sequestration was prominent. Despite a large number of trials, almost all of them compared medical interventions among themselves, or surgical interventions among themselves, rather than comparing between these two groups of treatment even though both groups of treatment could have been used. Our work shows that this issue of sequestration is widespread in surgical vs. pharmaceutical interventions, and that even where comparisons exist, there are too few, as well as often biased trials.

Conclusion

This study suggests that comparisons of pharmaceutical and surgical interventions are infrequent. The available comparisons have very few included studies which makes heterogeneity, and bias hard to quantify and may yield spurious results with the normality assumptions underpinning common frequentist meta-analytical approaches[62]. That is, even for the comparisons that have been retrieved the evidence is not sufficient.

For peer review only

For peer review only - http://bmjopen.bmj.com/site/about/guidelines.xhtml

Even accepting the difficulties in performing RCTs involving surgical interventions, our results still indicate a need for more comparative effectiveness research and for improved communication between surgical and medical specialties to bridge this gap in evidence. There are, of course, barriers to this. Head-to-head comparisons of treatments are often disfavored by manufacturers leery of jeopardizing their product against that of a competitor [63,64], and incentives unfortunately exist for both surgical and medical practitioners to promote treatments they are able to offer. Moving forward, both medical and surgical professional societies should collaborate to design fair and unbiased trials, and funders should also keep such research on their radars to try and overcome these structural obstacles.

Future research

Future clinical research should try to expand the scope, volume, and methodological rigor of comparative evidence on surgical versus medical interventions. This work should involve both surgical and medical specialists and should also incorporate patient preferences. Long-term patient-centered outcomes, including both benefits and harms should become available to put surgical and medical practices into proper perspective.

For peer review only

Figure 1. PRISMA study selection flowchart

Table 1. Eligible comparisons of surgical versus medical interventions

**Surgical arm**	**Drug arm**	**Disease**	**No. of outcomes (studies)**
Cardiac surgery			
Transmyocardial lazer revascularization	Continued medication	Refractory angina	3 (7,7,6)
Surgical closure	IV indomethacin	Patent ductus arteriosus	1 (1)
Dermatology			
Surgical excision	Imiquimod	BCC	4 (1,1,1,1)
Surgical excision	MAL-PDT	BCC	3 (1,2,2)
Surgical excision	ALA-PDT	BCC	2 (1,1)
General surgery			
Lateral internal sphincterotomy	Medical therapy (mainly GTN Isosorbide dinitrate and Botox)	Anal fissure	1 (15)
Pancreatic resection	Chemoradiotherapy	Pancreatic cancer	1 (2)
Oesophagectomy	Chemoradiotherapy and/or radiotherapy	Oesophageal cancer	5 (5,3,1,1,1)
Laparoscopic fundoplication	Protein pump inhibitors	GERD	5 (3,3,4,3,2)
Surgery	Tamoxifen	Primary breast cancer	1 (3)
Neurosurgery			
Decompressive surgery	Prednisolone	Leprosy	4 (1,1,1,1)
Epilepsy surgery	Continued antiepileptic drugs	Epilepsy	2 (2,1)
Decompressive craniectomy	Medical treatment (including barbiturates)	High ICP in closed TBI	2 (3,3)
Surgical decompression	Osmotic agents, blood pressure control, and glucose control	Cerebral oedema in acute ischaemic stroke	1 (3)
Surgical decompression	Dexamethasone, antihypertensives and intermittent diuresis	Primary supratentorial intracerebral haemorrhage	1 (9)
Obstetrics and gynaecology			
Suction aspiration	Vaginal suppositories or im inj. of 9-methylene-PGE2	Abortion	3 (2,2,1)
Suction aspiration	Misoprostol	Abortion	2 (22,9)
Suction aspiration	Vaginal or oral misoprostol	Abortion	3 (15,13,5)
Suction aspiration	Misoprostol and mifepristone	Abortion	2 (2,1)
Dilatation and curretage	Misoprostol	Abortion	2 (1,2)
Dilation and evacuation	Misoprostol	Abortion	1 (1,1)
Laparoscopic ovarian drilling	Medical ovulation induction	Infertility due to PCOS	2 (9,14)
Laparoscopic ovarian drilling	Letrozele	Infertility due to PCOS	2 (3,1)
Laparoscopic ovarian drilling	Gonadotropins	PCOS	2 (1,1)
Laparoscopic ovarian drilling	Metformin, clomiphene	PCOS	1 (2)
Laparoscopic ovarian drilling	Letrozele	PCOS	1 (1)
Laparoscopic ovarian drilling	Metformin, letrozele	PCOS	1 (1)
Laparoscopic ovarian drilling	Metformin	PCOS	2 (2,1)
Transcervical resection of endometrium using rollerball coagulation	Hormone therapy or antifibrinolytic	Heavy menstrual bleeding	7 (1,1,1,1,1,1,1)
Ophthalmology			

For peer review only - http://bmjopen.bmj.com/site/about/guidelines.xhtml

Amniotic membrane transplantation and medication	Lubrication, antibiotics, and pressure lowering medication	Acute ocular burns	1 (1)
Laser surgery	intravitreal anti-VEGF	Pathological myopia	2 (1,1)
iStent	Latanoprost/timolol	Open angle glaucoma	1 (2)
Argon laser trabeculoplasty	IOP reducing medication	Open angle glaucoma	3 (3,2,2)
Surgical correction	Botulinum toxin	Strabismus	2 (2,1)
	Orthopaedic surgery		
Open section of the carpal ligament	NSAID and splinting or corticosteroid injections	Carpal tunnel syndrome	1 (2)
Open surgery	Corticosteroid injection	Trigger finger	1 (2)
Decompressive surgery with or without fusion	Epidural steroid injection	Lumbar spinal stenosis	3 (1,1,1)
Open unilateral sympathectomy (L2-4)	IV prostanoid iloprost	Critical limb ischaemia	1 (1)
Surgical rotator cuff repair	Non-operative treatment including corticosteroid injection and exercise	Rotator cuff tear	1 (1)
Arthroscopic surgery	Sclerosing injection	Jumper's knee	3 (1,1,1)
	Otolaryngology		
Surgical orbital decompression	IV Methylprednisolone 1x3 followed by oral prednisolone	Thyroid eye disease	1 (1)
Grommets (ventilation tubes)	Antibiotic prophylaxis	Recurrent acute otitis media	1 (2)
Tonsillectomy or adrenotonsillectomy	Watchful waiting with or without analgesics and antibiotics	Tonsillitis	5 (5,4,5,2,2)
	Thoracic surgery		
Open thoracotomy	Thoracostomy drainage (with fibrinolytics)	Pleural empyema	1 (1)
VATS	Thoracostomy drainage (with fibrinolytics)	Pleural empyema	1 (7)
	Urology		
Surgical reimplantation of ureters	Antibiotics	Primary vesicoureteric reflux	1 (1)
	Vascular surgery		
Carotid endarterectomy and Aspirin 325 mg daily	Aspirin 325 mg daily	Asymptomatic carotid stenosis	1 (2)
Aspirin and carotid surgery	Aspirin	Carotid stenosis	2 (3,3)
Saphenofemoral disconnection	Therapeutic LMWH	Superficial thrombophlebitis	2 (1,1)
Surgery including primary amputation	Thrombolysis (w/ rt-Pa or urokinase)	Acute limb ischaemia	1 (3)

Abbreviations

BCC: basal cell carcinoma of the skin; GERD: Gastro-oesophageal reflux disease; GTN: glyceryl tri-nitrate; IOP: intra-ocular pressure; PCOS: polycystic ovarian syndrome; QOL: Quality of life

For peer review only

Table 2. Comparisons where the surgical treatment was superior to the drug treatment

Surgical arm	Drug arm	Disease	Outcome	Treatment effect (95% CI)	GRADE assessment
Transmyocardial lazer revascularization	Continued medication	Refractory angina	Angina reduction	OR=4.63 (3.43-6.25)	Low
Surgical excision	Imiquimod	BCC	Recurrence (3 y)	RR=0.1 (0.03-0.31)	Moderate
			Recurrence (5 y)	RR=0.13 (0.05-0.36)	Moderate
Surgical excision	MAL-PDT	BCC	Recurrence (3 y)	RR=0.04 (0-0.61)	Low
Surgical excision	ALA-PDT	BCC	Recurrence (3 y)	RR=0.09 (0.02-0.38)	Moderate
			Recurrence (5 y)	RR=0.08 (0.02-0.34)	Moderate
Laparoscopic fundoplication	Protein pump inhibitors	GERD	GORD-specific QOL (<1 y)	SMD=0.58 (0.46-0.7)	Low
Lateral internal sphincterotomy	Medical therapy (mainly GTN and Botox)	Anal fissure	Non-Healing (persistence or recurrence) 2 mo.	OR=0.11 (0.06-0.23)	High
Epilepsy surgery	Continued antiepileptic drugs	Epilepsy	Proportion (%) free from seizures (1 y)	RR=9.78 (4.73-20.2)*	Low
			Proportion free from all seizures incl. auras (1 y)	RR=15 (2.08-108.23)	Very Low
Surgical decompression	Osmotic agents, blood pressure control, and glucose control	Cerebral oedema in acute ischaemic stroke	Death at the end of follow-up	OR=0.19 (0.09-0.37)	
Surgical decompression	Dexamethasone, antihypertensives and intermittent diuresis	Primary supratentorial intracerebral haemorrhage	Death or dependence at end of follow up	OR=0.71 (0.58-0.88)	
Suction aspiration	Misoprostol	Abortion	Complete miscarriage	RR=1.11 (1.06-1.17)	Very Low
			Complete miscarriage	RR=1.04 (1.02-1.06)	Very Low
Dilatation and curettage	Misoprostol	Abortion	Complete miscarriage	RR=1.18 (1.1-1.27)*	Very Low
Dilatation and evacuation	Misoprostol	Abortion	Combined major and minor complications	OR=0.12 (0.03-0.46)	
Laparoscopic ovarian drilling	Medical ovulation induction	Infertility due to PCOS	Multiple pregnancy	OR=0.34 (0.18-0.66)	Moderate
Laparoscopic ovarian drilling	Gonadotropins	PCOS	Menstrual regularity at 6 mo.	OR=19.2 (3.17-116)	Very Low
Transcervical resection of endometrium using rollerball	Hormone therapy or antifibrinolytic	Heavy menstrual bleeding	Control of bleeding (cure or improvement to acceptable level) 4 mo.	RR=2.66 (1.94-3.64)	Moderate

For peer review only

coagulation			Control of bleeding (cure or improvement to acceptable level) 2 y	RR=1.29 (1.06-1.57)	Low
			Overall satisfaction with treatment 4 mo.	RR=2.8 (1.96-3.99)	Moderate
			Overall satisfaction with treatment 2 y	RR=1.4 (1.13-1.74)	Moderate
			Adverse events at 4 months	RR=0.26 (0.15-0.46)	Moderate
Surgical correction	Botulinum toxin	Strabismus	Improved ocular alignment > 10 dioptres, adults	RR=2.63 (1.18-5.9)	Low
iStent	Latanoprost/timolo l	Open angle glaucoma	Proportion of participants who were drop-free 6-18 mo	RR=125 (17.8-884)	Very low
Argon laser trabeculoplasty	IOP reducing medication	Open angle glaucoma	Failure to control IOP	RR=0.8 (0.71-0.91)	
Arthroscopic surgery	Sclerosing injection	Jumper's knee	Knee pain (0-100, 12 mo.)	MD=-28.3 (-41.79- -14.81)	Low
			Participant global assessment of success (1-100, 12 mo.)	MD=33.9 (18.74-49.06)	Low
Decompressive surgery with or without fusion	Epidural steroid injection	Lumbar spinal stenosis	Zurich claudication questionnaire (symptom evaluation) 6 w	MD=-0.6 (-0.77- -0.43)	Low
Open unilateral sympathectomy (L2-4)	IV prostanoid iloprost		Complete ulcer healing w/o rest pain or major amputation (24 w)	RR=1.76 (1.35-2.29)	Low
Grommets (ventilation tubes)	Antibiotic prophylaxis	Recurrent acute otitis media	Proportion of patients who have no recurrences (6 mo.)	RR=1.68 (1.07-2.65)*	Very Low
Tonsillectomy or adrenotonsillectom y	Watchful waiting with or without analgesics and antibiotics	Tonsillitis	Episodes of sore throat of any severity (children)	MD=-0.56 (-1.04- -0.07)*	Moderate
			Sore throat days (children)	MD=-5.13 (-8.03- -2.2)*	Moderate
			Episodes of sore throat of any severity (adults)	-MD=3.61 (-7.92- -0.7)*	Moderate
			Sore throat days (adults)	MD=-10.64 (-15.52- -5.76)*	Moderate
Aspirin and carotid surgery	Aspirin	Carotid stenosis	Any stroke or operative death	RR=0.85 (0.77-0.95)*	Moderate

*our re-analysis using a random effects meta-analysis model shows that the 95% confidence interval includes the null (results are inconclusive)

RR: risk ratio; OR: odds ratio; HR: hazard ratio; MD: mean difference; SMD: standardized mean difference; BCC: basal cell carcinoma of the skin; GERD: Gastro-oesophageal reflux disease; GNT: glyceryl trinitrate; IOP: intra-ocular pressure; PCOS: polycystic ovarian syndrome; QOL: Quality of life

For peer review only - http://bmjopen.bmj.com/site/about/guidelines.xhtml

Table 3. Comparisons where the drug treatment was superior to the surgical treatment

Surgical arm	Drug arm	Disease	Outcome	Treatment effect (95% CI)	GRADE assessment
Surgical excision	Imiquimod	BCC	Observer-rated good/excellent cosmetic outcome	RR=0.59 (0.47-0.74)	Low
Surgical excision	MAL-PDT	BCC	Observer-rated good/excellent cosmetic outcome	RR=0.85 (0.79-0.92)*	Moderate
Surgical excision	MAL-PDT	BCC	Patient-rated good/excellent cosmetic outcome	RR=0.53 (0.44-0.65)*	Moderate
Oesophagectomy	Chemoradiotherapy and/or radiotherapy	Oesophageal cancer	Serious adverse event (3 months)	RR=1.73 (1.11-2.67)*	Very Low
			Short-term health-related QOL	MD=0.93 (0.24-1.62)	Very Low
Laparoscopic fundoplication	Protein pump inhibitors	GERD	Serious adverse events	RR=1.46 (1.01-2.11)	Very Low
Pancreatic resection	Chemoradiotherapy	Pancreatic cancer	Overall mortality (5 y)	HR=2.63 (1.72-4)*	Very Low
Laparoscopic ovarian drilling	Medical ovulation induction	Infertility due to PCOS	Live birth	OR=0.71 (0.54-0.92)	Low
Suction aspiration	Vaginal or oral misoprostol	Abortion	Surgical evacuation	RR=20 (9.1-50)	Very Low
Laser surgery	intravitreal anti-VEGF	Pathological myopia	Change in best corrected visual acuity	MD=0.22 (0.01-0.43)*	Low
Amniotic membrane transplantation and medication	Lubrication, Antibiotics and Pressure lowering medication	Acute ocular burns	Visual acuity at final follow-up	MD=-0.83 (-1.32- -0.34)	Very Low
Decompressive surgery with or without fusion	Epidural steroid injection	Lumbar spinal stenosis	Oswestry Disability index 6 w	MD=5.7 (0.57-10.83)	Low
			Pain intensity (VAS) 6 w	MD=2.4 (1.92-2.88)	Low
Tonsillectomy or adrenotonsillectomy	Watchful waiting with or without analgesics and antibiotics	Tonsillitis	Episodes of moderately or severely sore throat (children)	MD=0.62 (0.22-1.03)*	Low
Carotid endarterectomy and Aspirin 325 mg daily	Aspirin 325 mg daily	Asymptomatic carotid stenosis	Perioperative stroke or death, or stroke of any territory or type during follow up	RR=6.49 (2.53-16.61)	

*our re-analysis using a random effects meta-analysis model shows that the 95% confidence interval includes the null (results are inconclusive)

RR: risk ratio; OR: odds ratio; HR: hazard ratio; MD: mean difference; BCC: basal cell carcinoma of the skin; GERD: Gastro-oesophageal reflux disease; PCOS: polycystic ovarian syndrome

For peer review only - http://bmjopen.bmj.com/site/about/guidelines.xhtml

For peer review only

For peer review only - http://bmjopen.bmj.com/site/about/guidelines.xhtml

Table 4. GRADE assessment across specialties

Specialty	Very Low	Low	Moderate	High	None available
Cardiac surgery	0 (0)	1 (25)	0 (0)	2 (50)	1 (25)
Dermatology	0 (0)	3 (33)	6 (67)	0 (0)	0 (0)
General surgery	9 (69)	3 (23)	0 (0)	1 (8)	0 (0)
Neurosurgery	5 (50)	2 (20)	1 (10)	0 (0)	2 (20)
Obstetrics and gynecology	14 (45)	4 (13)	7 (23)	1 (3)	5 (16)
Ophthalmology	2 (20)	5 (50)	0 (0)	0 (0)	3 (30)
Orthopaedic surgery	2 (20)	6 (60)	1 (10)	0 (0)	1 (10)
Otolaryngology	1 (14)	1 (14)	4 (57)	0 (0)	1 (14)
Thoracic surgery	0 (0)	1 (50)	1 (50)	0 (0)	0 (0)
Urology	0 (0)	0 (0)	0 (0)	0 (0)	1 (100)
Vascular surgery	0 (0)	1 (17)	2 (33)	0 (0)	3 (50)

For peer review only

References

1 Howick J, Koletsi D, Ioannidis JPA, *et al.* Most healthcare interventions tested in Cochrane Reviews are not effective according to high quality evidence: a systematic review and meta-analysis. *J Clin Epidemiol*. 2022;S0895-4356(22)00100-7.

2 Ergina PL, Cook JA, Blazeby JM, *et al.* Challenges in evaluating surgical innovation. *The Lancet*. 2009;374:1097–104.

3 Maruthappu M, Gilbert BJ, El-Harasis MA, *et al.* The influence of volume and experience on individual surgical performance: a systematic review. *Ann Surg*. 2015;261:642–7.

4 Martling A, Cedermark B, Johansson H, *et al.* The surgeon as a prognostic factor after the introduction of total mesorectal excision in the treatment of rectal cancer. *British Journal of Surgery*. 2002;89:1008–13.

5 McGrath PD. Relation Between Operator and Hospital Volume and Outcomes Following Percutaneous Coronary Interventions in the Era of the Coronary Stent. *JAMA*. 2000;284:3139.

6 Gelijns AC, Ascheim DD, Parides MK, *et al.* Randomized trials in surgery. *Surgery*. 2009;145:581–7.

7 Barkun JS, Aronson JK, Feldman LS, *et al.* Evaluation and stages of surgical innovations. *Lancet*. 2009;374:1089–96.

8 McCulloch P, Altman DG, Campbell WB, *et al.* No surgical innovation without evaluation: the IDEAL recommendations. *Lancet*. 2009;374:1105–12.

9 Janiaud P, Agarwal A, Tzoulaki I, *et al.* Validity of observational evidence on putative risk and protective factors: appraisal of 3744 meta-analyses on 57 topics. *BMC Med*. 2021;19:157.

10 Belbasis L, Bellou V, Ioannidis JPA. Conducting umbrella reviews. *bmjmed*. 2022;1:e000071.

11 Chapter V: Overviews of Reviews. https://training.cochrane.org/handbook/current/chapter-v (accessed 7 November 2023)

12 Page MJ, McKenzie JE, Bossuyt PM, *et al.* The PRISMA 2020 statement: an updated guideline for reporting systematic reviews. *BMJ*. 2021;n71.

13 [Dataset] Zavalis E, Saraswathula A, Rameau A, *et al.* Availability of evidence and comparative effectiveness for surgical versus drug interventions: an overview of systematic reviews. 2023. https://doi.org/10.17605/OSF.IO/RK7HU

14 Balshem H, Helfand M, Schünemann HJ, *et al.* GRADE guidelines: 3. Rating the quality of evidence. *Journal of Clinical Epidemiology*. 2011;64:401–6.

For peer review only - http://bmjopen.bmj.com/site/about/guidelines.xhtml

15 Hartung J, Knapp G. A refined method for the meta-analysis of controlled clinical trials with binary outcome. *Stat Med*. 2001;20:3875–89.

16 IntHout J, Ioannidis JPA, Borm GF. The Hartung-Knapp-Sidik-Jonkman method for random effects meta-analysis is straightforward and considerably outperforms the standard DerSimonian-Laird method. *BMC Med Res Methodol*. 2014;14:25.

17 Weber F, Knapp G, Ickstadt K, *et al.* Zero-cell corrections in random-effects meta-analyses. *Res Syn Meth*. 2020;11:913–9.

18 R: The R Project for Statistical Computing. https://www.r-project.org/ (accessed 29 March 2022)

19 Ioannidis JPA. The Proposal to Lower *P* Value Thresholds to .005. *JAMA*. 2018;319:1429.

20 Stanley TD, Doucouliagos H, Ioannidis JPA, *et al.* Detecting publication selection bias through excess statistical significance. *Research Synthesis Methods*. 2021;12:776–95.

21 Viechtbauer W. Conducting Meta-Analyses in *R* with the **metafor** Package. *J Stat Soft*. 2010;36. doi: 10.18637/jss.v036.i03

22 Phelps PO, Abariga SA, Cowling BJ, *et al.* Antimetabolites as an adjunct to dacryocystorhinostomy for nasolacrimal duct obstruction. *Cochrane Database Syst Rev*. 2020;4:CD012309.

23 Rees K, Beranek-Stanley M, Burke M, *et al.* Hypothermia to reduce neurological damage following coronary artery bypass surgery. *Cochrane Database Syst Rev*. 2001;CD002138.

24 Hoare BJ, Wallen MA, Imms C, *et al.* Botulinum toxin A as an adjunct to treatment in the management of the upper limb in children with spastic cerebral palsy (UPDATE). *Cochrane Database Syst Rev*. 2010;2010:CD003469.

25 Shylasree TS, Bryant A, Howells RE. Chemoradiation for advanced primary vulval cancer. *Cochrane Database Syst Rev*. 2011;2011:CD003752.

26 Lansbury L, Leonardi-Bee J, Perkins W, *et al.* Interventions for non-metastatic squamous cell carcinoma of the skin. *Cochrane Database Syst Rev*. 2010;CD007869.

27 Moreno G, Corbalán J, Peñaloza B, *et al.* Topical corticosteroids for treating phimosis in boys. *Cochrane Database Syst Rev*. 2014;CD008973.

28 Mitra S, Scrivens A, von Kursell AM, *et al.* Early treatment versus expectant management of hemodynamically significant patent ductus arteriosus for preterm infants. *Cochrane Database Syst Rev*. 2020;12:CD013278.

29 Flower A, Liu JP, Lewith G, *et al.* Chinese herbal medicine for endometriosis. *Cochrane Database Syst Rev*. 2012;CD006568.

For peer review only - http://bmjopen.bmj.com/site/about/guidelines.xhtml

30 Görk AS, Ehrenkranz RA, Bracken MB. Continuous infusion versus intermittent bolus doses of indomethacin for patent ductus arteriosus closure in symptomatic preterm infants. *Cochrane Database Syst Rev*. 2008;2008:CD006071.

31 Zhao J-G, Wang J, Huang W-J, *et al.* Interventions for treating simple bone cysts in the long bones of children. *Cochrane Database Syst Rev*. 2017;2:CD010847.

32 Bettany-Saltikov J, Weiss H-R, Chockalingam N, *et al.* Surgical versus non-surgical interventions in people with adolescent idiopathic scoliosis. *Cochrane Database Syst Rev*. 2015;CD010663.

33 Kang D, Han J, Neuberger MM, *et al.* Transurethral radiofrequency collagen denaturation for the treatment of women with urinary incontinence. *Cochrane Database Syst Rev*. 2015;CD010217.

34 Colquitt JL, Pickett K, Loveman E, *et al.* Surgery for weight loss in adults. *Cochrane Database Syst Rev*. 2014;CD003641.

35 Palmer JS, Monk AP, Hopewell S, *et al.* Surgical interventions for symptomatic mild to moderate knee osteoarthritis. *Cochrane Database Syst Rev*. 2019;7:CD012128.

36 Laupattarakasem W, Laopaiboon M, Laupattarakasem P, *et al.* Arthroscopic debridement for knee osteoarthritis. *Cochrane Database Syst Rev*. 2008;CD005118.

37 Liu JP, Yang H, Xia Y, *et al.* Herbal preparations for uterine fibroids. *Cochrane Database Syst Rev*. 2013;CD005292.

38 Cirocchi R, Trastulli S, Pressi E, *et al.* Non-operative management versus operative management in high-grade blunt hepatic injury. *Cochrane Database Syst Rev*. 2015;2015:CD010989.

39 Page MJ, Massy-Westropp N, O'Connor D, *et al.* Splinting for carpal tunnel syndrome. *Cochrane Database Syst Rev*. 2012;2012:CD010003.

40 Nikolaidis I, Fouyas IP, Sandercock PA, *et al.* Surgery for cervical radiculopathy or myelopathy. *Cochrane Database Syst Rev*. 2010;2010:CD001466.

41 Cheuk DKL, Wong V, Wraige E, *et al.* Surgery for scoliosis in Duchenne muscular dystrophy. *Cochrane Database Syst Rev*. 2015;2015:CD005375.

42 Weitz M, Portz S, Laube GF, *et al.* Surgery versus non-surgical management for unilateral ureteric-pelvic junction obstruction in newborns and infants less than two years of age. *Cochrane Database Syst Rev*. 2016;7:CD010716.

43 Abraham A, Kumar S, Chaudhry S, *et al.* Surgical interventions for diaphyseal fractures of the radius and ulna in children. *Cochrane Database Syst Rev*. 2011;CD007907.

For peer review only

44 Chang MY, Coleman AL, Tseng VL, *et al.* Surgical interventions for vertical strabismus in superior oblique palsy. *Cochrane Database Syst Rev*. 2017;2017:CD012447.

45 Monk AP, Davies LJ, Hopewell S, *et al.* Surgical versus conservative interventions for treating anterior cruciate ligament injuries. *Cochrane Database Syst Rev*. 2016;4:CD011166.

46 Gosler MW, Testroote M, Morrenhof JW, *et al.* Surgical versus non-surgical interventions for treating humeral shaft fractures in adults. *Cochrane Database Syst Rev*. 2012;1:CD008832.

47 Smith TO, Donell S, Song F, *et al.* Surgical versus non-surgical interventions for treating patellar dislocation. *Cochrane Database Syst Rev*. 2015;CD008106.

48 Abudou M, Chen X, Kong X, *et al.* Surgical versus non-surgical treatment for thoracolumbar burst fractures without neurological deficit. *Cochrane Database Syst Rev*. 2013;CD005079.

49 Cataneo AJM, Cataneo DC, de Oliveira FHS, *et al.* Surgical versus nonsurgical interventions for flail chest. *Cochrane Database Syst Rev*. 2015;2015:CD009919.

50 Khalil HS, Nunez DA. Functional endoscopic sinus surgery for chronic rhinosinusitis. *Cochrane Database Syst Rev*. 2006;CD004458.

51 Tóth M, Shah A, Hu K, *et al.* Endoscopic cyclophotocoagulation (ECP) for open angle glaucoma and primary angle closure. *Cochrane Database Syst Rev*. 2019;2:CD012741.

52 Fakhry F, Fokkenrood HJ, Spronk S, *et al.* Endovascular revascularisation versus conservative management for intermittent claudication. *Cochrane Database Syst Rev*. 2018;3:CD010512.

53 Araujo ST, Moreno DH, Cacione DG. Percutaneous thrombectomy or ultrasound-accelerated thrombolysis for initial management of acute limb ischaemia. *Cochrane Database Syst Rev*. 2022;1:CD013486.

54 Jenks S, Yeoh SE, Conway BR. Balloon angioplasty, with and without stenting, versus medical therapy for hypertensive patients with renal artery stenosis. *Cochrane Database Syst Rev*. 2014;2014:CD002944.

55 O'Connell NE, Wand BM, McAuley J, *et al.* Interventions for treating pain and disability in adults with complex regional pain syndrome. *Cochrane Database Syst Rev*. 2013;2013:CD009416.

56 Korfitsen CB, Mikkelsen M-LK, Ussing A, *et al.* Usefulness of Cochrane Reviews in Clinical Guideline Development-A Survey of 585 Recommendations. *Int J Environ Res Public Health*. 2022;19:685.

57 Hozo I, Djulbegovic B, Parish AJ, *et al.* Identification of threshold for large (dramatic) effects that would obviate randomized trials is not possible. *Journal of Clinical Epidemiology*. 2022;145:101–11.

For peer review only - http://bmjopen.bmj.com/site/about/guidelines.xhtml

58 Kolaski K, Logan LR, Ioannidis JPA. Guidance to best tools and practices for systematic reviews. *Syst Rev*. 2023;12:96.

59 Goldkuhle M, Narayan VM, Weigl A, *et al.* A systematic assessment of Cochrane reviews and systematic reviews published in high-impact medical journals related to cancer. *BMJ Open*. 2018;8:e020869.

60 Kim DD, Tang JY, Ioannidis JPA. Network geometry shows evidence sequestration for medical vs. surgical practices: treatments for basal cell carcinoma. *Journal of Clinical Epidemiology*. 2014;67:391–400.

61 Hirshman BR, Tang JA, Jones LA, *et al.* Impact of medical academic genealogy on publication patterns: An analysis of the literature for surgical resection in brain tumor patients: Medical Academic Genealogy. *Ann Neurol*. 2016;79:169–77.

62 Jackson D, White IR. When should meta-analysis avoid making hidden normality assumptions? *Biometrical Journal*. 2018;60:1040–58.

63 Flacco ME, Manzoli L, Boccia S, *et al.* Head-to-head randomized trials are mostly industry sponsored and almost always favor the industry sponsor. *Journal of Clinical Epidemiology*. 2015;68:811–20.

64 Lathyris DN, Patsopoulos NA, Salanti G, *et al.* Industry sponsorship and selection of comparators in randomized clinical trials. *European Journal of Clinical Investigation*. 2010;40:172–82.

For peer review only

For peer review only - http://bmjopen.bmj.com/site/about/guidelines.xhtml

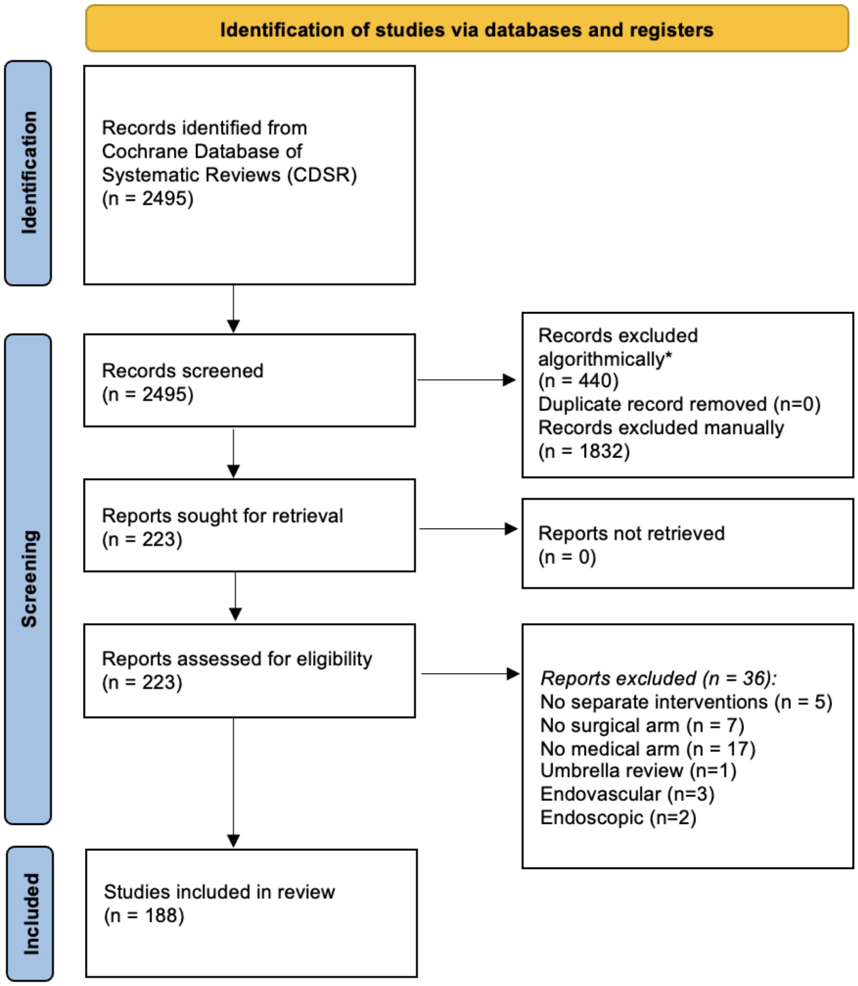

Figure 1. PRISMA study selection flowchart

219x227mm (118 x 118 DPI)

Availability of evidence and comparative effectiveness for surgical versus drug interventions: an overview of systematic reviews

Emmanuel A. Zavalis[1,2*], Anaïs Rameau[3*], Anirudh Saraswathula[4*], Joachim Vist[1], Ewoud Schuit[5,6],

John P. A. Ioannidis[2,7]

*co-first authors

1 Department of Learning Informatics Management and Ethics, Karolinska Institutet, Stockholm, Sweden

2 Meta-Research Innovation Center at Stanford (METRICS), Stanford University, Stanford, CA, USA

3 Sean Parker Institute for the Voice, Department of Otolaryngology–Head and Neck Surgery, Weill Cornell Medical College, New York, NY, USA

4 Department of Otolaryngology–Head and Neck Surgery, Johns Hopkins University School of Medicine, Baltimore, MD, USA

5 Julius Center, University Medical Center Utrecht, Utrecht University, Utrecht, the Netherlands

6 Cochrane Netherland, University Medical Center Utrecht, Utrecht University, Utrecht, the Netherlands

7 Stanford Prevention Research Center, Department of Medicine, and Department of Epidemiology and Population Health, Stanford University School of Medicine, Stanford, CA, USA

Supplementary Materials - Index

Supplementary Data

Supplement 1 – List of included studies *pag. 2*

Supplementary Figures and Tables

Supplementary Table 1 *pag. 7*

Supplementary Table 2 *pag. 8*

For peer review only

Supplementary Data

Supplement 1 – List of included studies

CDSR_ID	Title	Specialty	Comparison available
CD005624.PUB4	Interventions for great saphenous vein incompetence	vascular surgery	No
CD006931.PUB2	Submacular surgery for choroidal neovascularisation secondary to age-related macular degeneration	ophthalmology	No
CD002764.PUB2	Surgery for the resolution of symptoms in malignant bowel obstruction in advanced gynaecological and gastrointestinal cancer	general surgery	No
CD007119.PUB2	Interventions for restoring patency of occluded central venous catheter lumens	vascular surgery	No
CD008509.PUB3	Alpha-blockers as medical expulsive therapy for ureteral stones	urology	No
CD013085.PUB2	Balneotherapy for chronic venous insufficiency	vascular surgery	No
CD009959.PUB2	Interventions for the treatment of Frey's syndrome	otolaryngology	No
CD004008.PUB3	Interventions for trachoma trichiasis	ophthalmology	No
CD006134.PUB5	Oral contraceptives for functional ovarian cysts	obstetrics and gynecology	No
CD011650.PUB2	Management of people with early- or very early-stage hepatocellular carcinoma	general surgery	No
CD001081.PUB4	Carotid endarterectomy for symptomatic carotid stenosis	vascular surgery	Yes
CD010244.PUB2	Resection versus other treatments for locally advanced pancreatic cancer	general surgery	Yes
CD012432.PUB2	Interventions for managing medication-related osteonecrosis of the jaw	otolaryngology	No
CD010260.PUB2	Hysterectomy with radiotherapy or chemotherapy or both for women with locally advanced cervical cancer	obstetrics and gynecology	No
CD012602.PUB2	Methods for managing miscarriage: a network meta-analysis	obstetrics and gynecology	Yes
CD006983.PUB3	Decompressive surgery for treating nerve damage in leprosy	neurosurgery	Yes
CD009590.PUB2	Endometriosis: an overview of Cochrane Reviews	obstetrics and gynecology	No
CD005320.PUB2	Operative and non-operative treatment options for dislocation of the hip following total hip arthroplasty	orthopaedic surgery	No
CD010349.PUB2	Iodine-131-meta-iodobenzylguanidine therapy for patients with newly diagnosed high-risk neuroblastoma	neurosurgery	No
CD010712	Nonoperative treatment for lumbar spinal stenosis with neurogenic claudication	orthopaedic surgery	No
CD011478.PUB2	Type II or type III radical hysterectomy compared to chemoradiotherapy as a primary intervention for stage IB2 cervical cancer	obstetrics and gynecology	No
CD002116.PUB2	Drug treatment for faecal incontinence in adults	general surgery	No
CD005029.PUB2	Treatment for ataxia in multiple sclerosis	neurosurgery	No
CD008107.PUB2	Perioperative chemo(radio)therapy versus primary surgery for resectable adenocarcinoma of the stomach, gastroesophageal junction, and lower esophagus	general surgery	No
CD008602.PUB4	Interventions for congenital talipes equinovarus (clubfoot)	orthopaedic surgery	No
CD004461.PUB3	Interventions for recurrent idiopathic epistaxis (nosebleeds) in children	otolaryngology	No
CD006476.PUB3	Management for intussusception in children	general surgery	No
CD009166.PUB2	Cervical stitch (cerclage) for preventing preterm birth in multiple pregnancy	obstetrics and gynecology	No
CD002221.PUB2	Interventions for involutional lower lid entropion	ophthalmology	No
CD009379.PUB2	Amniotic membrane transplantation for acute ocular burns	ophthalmology	Yes
CD003296.PUB3	Retinoids for preventing the progression of cervical intra-epithelial neoplasia	obstetrics and gynecology	No
CD004917.PUB3	Interventions for infantile esotropia	ophthalmology	No
CD003431.PUB3	Non surgical therapy for anal fissure	general surgery	Yes
CD007340.PUB2	Bariatric surgery for non-alcoholic steatohepatitis in obese patients	general surgery	No

For peer review only

For peer review only - http://bmjopen.bmj.com/site/about/guidelines.xhtml

CDSR_ID	Title	Specialty	Comparison available
CD001122.PUB5	Laparoscopic ovarian drilling for ovulation induction in women with anovulatory polycystic ovary syndrome	obstetrics and gynecology	Yes
CD007156.PUB2	Interventions for the management of oral submucous fibrosis	otolaryngology	No
CD012802.PUB2	Ab interno supraciliary microstent surgery for open-angle glaucoma	ophthalmology	No
CD004399.PUB3	Medical versus surgical interventions for open angle glaucoma	ophthalmology	No
CD009266.PUB2	Non-steroidal antiandrogen monotherapy compared with luteinising hormone-releasing hormone agonists or surgical castration monotherapy for advanced prostate cancer	urology	No
CD010273.PUB2	Interventions for treating postpartum constipation	general surgery	No
CD009366.PUB2	Lumbar sympathectomy versus prostanoids for critical limb ischaemia due to non-reconstructable peripheral arterial disease	orthopaedic surgery	Yes
CD007060.PUB2	Liver resection versus other treatments for neuroendocrine tumours in patients with resectable liver metastases	general surgery	No
CD008088.PUB3	Anti-TNF-Œ± treatment for pelvic pain associated with endometriosis	obstetrics and gynecology	No
CD004982.PUB6	Treatment for superficial thrombophlebitis of the leg	vascular surgery	Yes
CD007939.PUB2	Single herbal medicine for diabetic retinopathy	ophthalmology	No
CD002000.PUB3	Bypass surgery for chronic lower limb ischaemia	vascular surgery	No
CD012017.PUB2	Grommets (ventilation tubes) for recurrent acute otitis media in children	otolaryngology	Yes
CD009968.PUB2	Botulinum toxin for upper oesophageal sphincter dysfunction in neurological swallowing disorders	general surgery	No
CD004272.PUB3	Surgery versus primary endocrine therapy for operable primary breast cancer in elderly women (70 years plus)	general surgery	Yes
CD007118.PUB2	Palliative cytoreductive surgery versus other palliative treatments in patients with unresectable liver metastases from gastro-entero-pancreatic neuroendocrine tumours	general surgery	No
CD006714.PUB2	Surgical versus medical methods for second trimester induced abortion	obstetrics and gynecology	Yes
CD011174.PUB2	Interventions for non-tubal ectopic pregnancy	obstetrics and gynecology	No
CD010541.PUB3	Surgery for epilepsy	neurosurgery	Yes
CD013034.PUB2	Surgery for patellar tendinopathy (jumper's knee)	orthopaedic surgery	Yes
CD007481.PUB3	Chemical pleurodesis versus surgical intervention for persistent and recurrent pneumothoraces in cystic fibrosis	thoracic surgery	No
CD003712.PUB3	Transmyocardial laser revascularization versus medical therapy for refractory angina	cardiac surgery	Yes
CD008997.PUB2	Non-resection versus resection for an asymptomatic primary tumour in patients with unresectable Stage IV colorectal cancer	general surgery	No
CD005081.PUB3	Medical and surgical treatment for ocular myasthenia	ophthalmology	No
CD013099.PUB2	Interventions for bacterial folliculitis and boils (furuncles and carbuncles)	general surgery	No
CD011837.PUB2	Medical and surgical interventions for the treatment of usual-type vulval intraepithelial neoplasia	obstetrics and gynecology	No
CD003951.PUB3	Surgical versus medical treatment with cyclooxygenase inhibitors for symptomatic patent ductus arteriosus in preterm infants	cardiac surgery	Yes
CD007261.PUB2	Interventions for managing temporomandibular joint osteoarthritis	orthopaedic surgery	No
CD003193.PUB4	Anticholinergic drugs versus non-drug active therapies for non-neurogenic overactive bladder syndrome in adults	urology	No
CD009493.PUB2	N-acetylcarnosine (NAC) drops for age-related cataract	ophthalmology	No
CD005198.PUB3	Therapeutic interventions for Burkitt lymphoma in children	otolaryngology	No
CD004981.PUB4	Treatment for femoral pseudoaneurysms	vascular surgery	No
CD003525.PUB2	Surgery for lateral elbow pain	orthopaedic surgery	No
CD013006.PUB2	Interventions for the management of obesity in people with bipolar disorder	general surgery	No
CD013404.PUB2	Surgical interventions for treating intracapsular hip fractures in older adults: a network meta-analysis	orthopaedic surgery	No
CD011725.PUB2	Indomethacin for intracranial hypertension secondary to severe traumatic brain injury in adults	neurosurgery	No

For peer review only - http://bmjopen.bmj.com/site/about/guidelines.xhtml

CDSR_ID	Title	Specialty	Comparison available
CD009526.PUB2	Ovarian surgery for symptom relief in women with polycystic ovary syndrome	obstetrics and gynecology	Yes
CD003855.PUB3	Surgery versus medical therapy for heavy menstrual bleeding	obstetrics and gynecology	Yes
CD009505.PUB2	Aromatase inhibitors for uterine fibroids	obstetrics and gynecology	No
CD003037.PUB2	Medical versus surgical methods for first trimester termination of pregnancy	obstetrics and gynecology	Yes
CD011169.PUB2	Selective oestrogen receptor modulators (SERMs) for endometriosis	obstetrics and gynecology	No
CD007924.PUB3	Medical interventions for high-grade vulval intraepithelial neoplasia	obstetrics and gynecology	No
CD008111.PUB2	Thymectomy for non-thymomatous myasthenia gravis	thoracic surgery	No
CD007223.PUB4	Medical treatments for incomplete miscarriage	obstetrics and gynecology	Yes
CD010308.PUB2	Interventions for melanoma in situ, including lentigo maligna	general surgery	No
CD007468.PUB4	Surgical interventions for the early management of Bell's palsy	neurosurgery	No
CD007792.PUB2	Palliative surgery versus medical management for bowel obstruction in ovarian cancer	general surgery	No
CD008455.PUB2	Interventions for treating bisphosphonate-related osteonecrosis of the jaw (BRONJ)	orthopaedic surgery	No
CD002115.PUB5	Management of faecal incontinence and constipation in adults with central neurological diseases	general surgery	No
CD006991.PUB2	Surgical versus medical interventions for chronic rhinosinusitis with nasal polyps	otolaryngology	No
CD001496.PUB2	Pharmacological and surgical interventions for the treatment of gastro-oesophageal reflux in adults and children with asthma	general surgery	No
CD008571.PUB2	Interventions for women with endometrioma prior to assisted reproductive technology	obstetrics and gynecology	No
CD006544.PUB3	Prostanoids for critical limb ischaemia	vascular surgery	No
CD003435.PUB2	Surgical decompression for cerebral oedema in acute ischaemic stroke	neurosurgery	Yes
CD013325.PUB2	Interventions for treating people with symptoms of bladder pain syndrome: a network meta-analysis	urology	No
CD001066.PUB3	Interventions for varicose veins and leg oedema in pregnancy	vascular surgery	No
CD006388.PUB2	Octreotide for the treatment of chylothorax in neonates	thoracic surgery	No
CD003658.PUB3	Needling for encapsulated trabeculectomy filtering blebs	ophthalmology	No
CD006152.PUB2	Decompressive surgery of lower limbs for symmetrical diabetic peripheral neuropathy	orthopaedic surgery	No
CD001896.PUB2	Surgical interruption of pelvic nerve pathways for primary and secondary dysmenorrhoea	obstetrics and gynecology	No
CD004699.PUB2	Surgery for local and locally advanced non-small cell lung cancer	thoracic surgery	No
CD002867	Treatments for secondary postpartum haemorrhage	obstetrics and gynecology	No
CD006373.PUB2	Interventions for treating functional dysphonia in adults	otolaryngology	No
CD001541.PUB3	Interventions for ingrowing toenails	general surgery	No
CD013469.PUB2	Surgical and medical interventions for abdominal aortic graft infections	vascular surgery	No
CD001219	Corticosteroids for the resolution of malignant bowel obstruction in advanced gynaecological and gastrointestinal cancer	general surgery	No
CD005304.PUB3	Interventions for primary (intrinsic) tracheomalacia in children	thoracic surgery	No
CD011498.PUB2	Non-surgical versus surgical treatment for oesophageal cancer	general surgery	Yes
CD002784.PUB3	Surgery versus thrombolysis for initial management of acute limb ischaemia	vascular surgery	Yes
CD006499.PUB4	Botulinum toxin for the treatment of strabismus	ophthalmology	Yes
CD005024.PUB3	Surgery for traumatic optic neuropathy	general surgery	No
CD003243.PUB3	Laparoscopic fundoplication surgery versus medical management for gastro-oesophageal reflux disease (GORD) in adults	general surgery	Yes
CD003118.PUB2	Interventions for the treatment of Morton's neuroma	orthopaedic surgery	No
CD001001.PUB3	Lung volume reduction surgery for diffuse emphysema	thoracic surgery	No
CD010784.PUB3	Medical and surgical interventions for the treatment of urinary stones in children	urology	No

For peer review only - http://bmjopen.bmj.com/site/about/guidelines.xhtml

CDSR_ID	Title	Specialty	Comparison available
CD000324.PUB2	Interventions for tubal ectopic pregnancy	obstetrics and gynecology	No
CD000526.PUB2	Interventions for treating tuberculous pericarditis	cardiac surgery	No
CD004156.PUB4	Treatment for spasticity in amyotrophic lateral sclerosis/motor neuron disease	neurosurgery	No
CD004159.PUB3	Treatment for meralgia paraesthetica	neurosurgery	No
CD006797.PUB2	Surgical resection versus non-surgical treatment for hepatic node positive patients with colorectal liver metastases	general surgery	No
CD007510.PUB3	Botulinum toxin for masseter hypertrophy	otolaryngology	No
CD011523.PUB2	Medical versus surgical treatment for refractory or recurrent peptic ulcer	general surgery	No
CD001802.PUB3	Tonsillectomy or adenotonsillectomy versus non-surgical treatment for chronic/recurrent acute tonsillitis	otolaryngology	Yes
CD007383.PUB3	Surgical versus non-surgical management of abdominal injury	general surgery	No
CD006981.PUB2	Treatment for sialorrhea (excessive saliva) in people with motor neuron disease/amyotrophic lateral sclerosis	otolaryngology	No
CD001829.PUB4	Interventions for treating oral leukoplakia to prevent oral cancer	otolaryngology	No
CD001934.PUB2	Surgical versus non-surgical interventions for vocal cord nodules	otolaryngology	No
CD003412.PUB3	Interventions for basal cell carcinoma of the skin	dermatology	Yes
CD003425.PUB4	Splenectomy versus conservative management for acute sequestration crises in people with sickle cell disease	general surgery	No
CD003983.PUB3	Decompressive craniectomy for the treatment of high intracranial pressure in closed traumatic brain injury	neurosurgery	Yes
CD004098.PUB2	Levothyroxine or minimally invasive therapies for benign thyroid nodules	general surgery	No
CD004437.PUB6	Thrombolytic therapy for pulmonary embolism	cardiac surgery	No
CD004927.PUB4	Surgical management of functional bladder outlet obstruction in adults with neurogenic bladder dysfunction	urology	No
CD005619.PUB3	Subacromial decompression surgery for rotator cuff disease	orthopaedic surgery	No
CD006032.PUB4	Steroids for traumatic optic neuropathy	ophthalmology	No
CD006746.PUB4	Laser peripheral iridoplasty for chronic angle closure	ophthalmology	No
CD007281.PUB2	Interventions for cutaneous Bowen's disease	dermatology	No
CD007404.PUB2	Interventions for central giant cell granuloma (CGCG) of the jaws	otolaryngology	No
CD007535.PUB4	Chinese herbal medicine for subfertile women with polycystic ovarian syndrome	obstetrics and gynecology	No
CD008280.PUB2	Interventions for atrophic rhinitis	otolaryngology	No
CD009244.PUB2	Interventions for anal canal intraepithelial neoplasia	general surgery	No
CD010287.PUB3	Aromatase inhibitors (letrozole) for subfertile women with polycystic ovary syndrome	obstetrics and gynecology	Yes
CD010651.PUB2	Surgical versus non-surgical management for pleural empyema	thoracic surgery	Yes
CD011160.PUB2	Anti-vascular endothelial growth factor for choroidal neovascularisation in people with pathological myopia	ophthalmology	Yes
CD012742.PUB2	Subconjunctival draining minimally-invasive glaucoma devices for medically uncontrolled glaucoma	ophthalmology	No
CD012743.PUB2	Ab interno trabecular bypass surgery with iStent for open-angle glaucoma	ophthalmology	Yes
CD012834.PUB2	Medical and surgical abortion for women living with HIV	obstetrics and gynecology	No
CD012879.PUB2	Shoulder replacement surgery for osteoarthritis and rotator cuff tear arthropathy	orthopaedic surgery	No
CD006131.PUB3	Interventions for Mooren's ulcer	dermatology	No
CD007677.PUB4	Pentoxifylline for the treatment of endometriosis-associated pain and infertility	obstetrics and gynecology	No
CD012740.PUB2	Ab interno trabecular bypass surgery with Schlemm´s canal microstent (Hydrus) for open angle glaucoma	ophthalmology	No
CD006151.PUB3	Fundoplication versus postoperative medication for gastro-oesophageal reflux in children with neurological impairment undergoing gastrostomy	general surgery	No
CD010081.PUB2	Interventions for hidradenitis suppurativa	dermatology	No
CD007630.PUB2	Surgical orbital decompression for thyroid eye disease	otolaryngology	Yes
CD011165.PUB2	Tonsillectomy or adenotonsillectomy versus non-surgical management for obstructive sleep-disordered breathing in children	otolaryngology	No

For peer review only - http://bmjopen.bmj.com/site/about/guidelines.xhtml

CDSR_ID	Title	Specialty	Comparison available
CD005656.PUB3	Intravitreal steroids for macular edema in diabetes	ophthalmology	No
CD009860.PUB2	Surgery for trigger finger	orthopaedic surgery	Yes
CD013502	Surgery for rotator cuff tears	orthopaedic surgery	Yes
CD002180	Surgery versus non-surgical treatment for bronchiectasis	thoracic surgery	No
CD010868.PUB2	Interventions for dissociated vertical deviation	ophthalmology	No
CD001408.PUB2	Botulinum toxin type A in the treatment of lower limb spasticity in children with cerebral palsy	orthopaedic surgery	No
CD003919.PUB2	Laser trabeculoplasty for open angle glaucoma	ophthalmology	Yes
CD010312.PUB2	Prostaglandins for management of retained placenta	obstetrics and gynecology	No
CD011693.PUB3	Ab interno trabecular bypass surgery with Trabectome for open-angle glaucoma	ophthalmology	No
CD008669.PUB3	Tonsillectomy for periodic fever, aphthous stomatitis, pharyngitis and cervical adenitis syndrome (PFAPA)	otolaryngology	No
CD008128.PUB2	Treatment of valvular heart disease during pregnancy for improving maternal and neonatal outcome	cardiac surgery	No
CD001923.PUB2	Carotid endarterectomy for asymptomatic carotid stenosis	vascular surgery	Yes
CD010960.PUB2	Injection therapies for Achilles tendinopathy	orthopaedic surgery	No
CD003738.PUB3	Interventions for preventing posterior capsule opacification	ophthalmology	No
CD013000.PUB2	Interventions for orbital lymphangioma	otolaryngology	No
CD008282	Adenoidectomy for recurrent or chronic nasal symptoms in children	otolaryngology	No
CD003263.PUB5	Interventions for vitiligo	dermatology	No
CD008583.PUB3	Ultrasound-guided transvaginal ovarian needle drilling for clomiphene-resistant polycystic ovarian syndrome in subfertile women	obstetrics and gynecology	No
CD007810.PUB2	Adenoidectomy for otitis media in children	otolaryngology	No
CD006181.PUB2	Prophylactic surgical ligation of patent ductus arteriosus for prevention of mortality and morbidity in extremely low birth weight infants	cardiac surgery	No
CD011917.PUB2	Surgery for limited-stage small-cell lung cancer	thoracic surgery	No
CD010264.PUB2	Surgical versus non-surgical treatment for lumbar spinal stenosis	orthopaedic surgery	Yes
CD008732.PUB2	Macular grid laser photocoagulation for branch retinal vein occlusion	ophthalmology	No
CD011680.PUB2	Interventions for necrotizing soft tissue infections in adults	general surgery	No
CD001801.PUB3	Grommets (ventilation tubes) for hearing loss associated with otitis media with effusion in children	otolaryngology	No
CD006205.PUB4	Interventions for the treatment of oral and oropharyngeal cancers: surgical treatment	otolaryngology	No
CD009245.PUB3	Interventions for the treatment of Paget's disease of the vulva	obstetrics and gynecology	No
CD012798.PUB3	Interventions for treating distal intestinal obstruction syndrome (DIOS) in cystic fibrosis	general surgery	No
CD008089.PUB2	Surgery for shoulder osteoarthritis	orthopaedic surgery	No
CD008497.PUB3	Deep brain and cortical stimulation for epilepsy	neurosurgery	No
CD004325.PUB2	Surgical versus non-surgical treatment for acute anterior shoulder dislocation	orthopaedic surgery	No
CD005048.PUB4	Interventions for dysphagia in oesophageal cancer	general surgery	No
CD000200.PUB2	Surgery for primary supratentorial intracerebral haemorrhage	neurosurgery	Yes
CD011031.PUB3	Laparoscopic surgery for endometriosis	obstetrics and gynecology	No
CD010796.PUB2	Surgery for treating hip impingement (femoroacetabular impingement)	orthopaedic surgery	No
CD006769.PUB2	Interventions for late trabeculectomy bleb leak	ophthalmology	No
CD001532.PUB5	Interventions for primary vesicoureteric reflux	urology	Yes
CD008104.PUB2	Interventions for treating osteochondral defects of the talus in adults	orthopaedic surgery	No
CD001552.PUB2	Surgical versus non-surgical treatment for carpal tunnel syndrome	orthopaedic surgery	Yes

For peer review only - http://bmjopen.bmj.com/site/about/guidelines.xhtml

Supplementary Figures and Tables

Supplementary table 1. Reviews per specialty

Specialty	Total reviews	Reviews with at least one comparison (%)
Cardiac surgery	6	2 (33)
Dermatology	5	1 (20)
General surgery	35	5 (14)
Neurosurgery	12	5 (42)
Obstetrics and gynecology	31	8 (26)
Ophthalmology	25	5 (20)
Orthopaedic surgery	23	6 (26)
Otolaryngology	23	3 (13)
Thoracic surgery	9	1 (11)
Urology	7	1 (14)
Vascular surgery	12	4 (33)

For peer review only

Supplementary Table 2. Inconclusive comparisons between surgery and drugs

Surgical arm	Drug arm	Disease	Outcome	Treatment effect (95% CI)	GRADE assessment
		Cardiac surgery			
Transmyocardial lazer revascularization	Continued medication	Refractory angina	Overall mortality	OR=1.12 (0.77-1.63)	High
			Postoperative mortality (30 d)	OR=1.19 (0.63-2.24)	High
Surgical closure	IV indomethacin	Patent ductus arteriosus	Death before discharge	RR=0.67 (0.34-1.31)	
		Dermatology			
Surgical excision	Imiquimod	BCC	Patient-rated good/excellent cosmetic outcome	RR=1 (0.94-1.06)	Low
		General surgery			
Surgery	Tamoxifen	Primary breast cancer	Overall survival	HR=0.98 (0.81-1.2)	Low
Laparoscopic fundoplication	Protein pump inhibitors	GERD	Health-related quality of life (<1 y)	SMD=0.14 (-0.02-0.3)+	Very Low
			Health-related QOL (1-5 y)	SMD=0.03 (-0.19-0.24)+	Very Low
			GORD-specific quality of life (1-5 y)	SMD=0.28 (-0.27-0.84)+	Very Low
Oesophagectomy	Chemoradiotherapy and/or radiotherapy	Oesophageal cancer	Short-term mortality	RR=0.39 (0.11-1.35)	Very Low
			Long-term mortality	RR=1.03 (0.92-1.14)	Low
			Medium-term health-related QOL	MD=-0.95 (-2.1-0.2)	Very Low
		Neurosurgery			
Decompressive surgery	Prednisolone	Leprosy	Change in sensory score after one year	MD=0.08 (-2.45-2.61)	Very Low
			Proportion of ulnar nerves with sensory improvement after one year	RR=1.13 (0.71-1.77)	Very Low
			Change in motor score after one year	MD=0.82 (-1.34-2.98)	Very Low
			Proportion of ulnar nerves with motor improvement after one year	RR=0.91 (0.64-1.28)	Very Low
Decompressive craniectomy	Medical treatment (including barbiturates)	High ICP in closed TBI	Neurological unfavourable outcome 6 mo	RR=1 (0.71-1.4)	Low
			Mortality 6 mo	RR=0.66 (0.43-1.01)	Moderate
		Obstetrics and gynaecology			
Suction aspiration	Vaginal or oral misoprostol	Abortion	Death or serious complication	RR=1 (0.04-25)	

For peer review only

Surgical arm	Drug arm	Disease	Outcome	Treatment effect (95% CI)	GRADE assessment
Suction aspiration	Misoprostol	Abortion	Composite outcome of death or serious complication	RR=1.53 (0.45-5.16)	Very Low
Suction aspiration	Misoprostol and mifepristone	Abortion	Complete miscarriage	RR=1.29 (0.96-1.73)	Very Low
			Composite outcome of death or serious complication	RR=0.14 (0.01-2.74)	Very Low
Suction aspiration	Vaginal suppositories or im inj. of 9-methylene-PGE2	Abortion	Abortion not completeted with intended method	OR=0.62 (0.02-16.6)	
			Ongoing pregnancy	OR=1.82 (0.54-6.25)	
			Pelvic infection	OR=0.46 (0.14-1.56)	
Dilatation and curettage	Misoprostol	Abortion	Composite outcome of death or serious complication	RR=0.79 (0.34-1.85)	Very Low
Laparoscopic ovarian drilling	Metformin, Clomiphene	PCOS	Menstrual regularity at 6 mo.	OR=1.02 (0.64-1.64)	Very Low
Laparoscopic ovarian drilling	Letrozele	PCOS	Menstrual regularity at 6 mo.	OR=1.08 (0.64-1.84)	Very Low
Laparoscopic ovarian drilling	Metformin, Letrozol	PCOS	Menstrual regularity at 6 mo.	OR=0.95 (0.49-1.81)	Very Low
Laparoscopic ovarian drilling	Metformin	PCOS	Menstrual regularity at 6 mo.	OR=1.51 (0.62-3.71)	Moderate
Laparoscopic ovarian drilling	Gonadotropins	PCOS	Improvement in androgenic symptoms 6 mo.	OR=3.02 (0.56-16.33)	Low
Laparoscopic ovarian drilling	Metformin	PCOS	Improvement in androgenic symptoms 6 mo.	OR=1 (0.42-2.37)	Low
Laparoscopic ovarian drilling	Letrozele	Infertility due to PCOS	Live birth	RR=0.72 (0.5-1.05)	Moderate
			Rate of ovarian hyperstimulation syndrome	RD=0 (-0.01-0.01)	High
Transcervical resection of endometrium using rollerball coagulation	Hormone therapy or antifibrinolytic	Heavy menstrual bleeding	Control of bleeding (cure or improvement to acceptable level) 5 y	RR=1.14 (0.97-1.34)	Very Low
			Overall satisfaction with treatment 5 y	RR=1.13 (0.94-1.37)	Very Low
			Ophthalmology		
Amniotic membrane transplantation and medication	Lubrication, Antibiotics and Pressure lowering medication	Acute ocular burns	Epithelial defect 21 d post-injury	RR=0.71 (0.27-1.85)	Low
Argon laser trabeculoplasty	IOP reducing medication	Open angle glaucoma	Visual field progression	RR=0.7 (0.42-1.16)	
			Optic neuropathy progression	RR=0.71 (0.38-1.34)	
Laser surgery	intravitreal anti-VEGF	Pathological myopia	Proportion of participants with a	RR=0.32 (0.08-1.33)	Low

For peer review only - http://bmjopen.bmj.com/site/about/guidelines.xhtml

Surgical arm	Drug arm	Disease	Outcome	Treatment effect (95% CI)	GRADE assessment
			gain of 3+ lines in BCVA at 1 y		
Surgical correction	Botulinum toxin	Strabismus	Improved ocular alignment > 10 dioptres, children	RR=1.1 (0.86-1.41)	Low
		Orthopaedic surgery			
Arthroscopic surgery	Sclerosing injection	Jumper's knee	Withdrawal rate	OR=1 (0.06-16.89)	Very Low
Open surgery	Corticosteroid injection	Trigger finger	Resolution of triggering	RR=1.48 (0.79-2.76)	Very low
Open section of the carpal ligament	NSAID and splinting or corticosteroid injections	Carpal tunnel syndrome	Improvement in clinical symptoms at three months of follow-up	RR=1.09 (0.91-1.32)	
Surgical rotator cuff repair	Non-operative treatment including corticosteroid injection and exercise	Rotator cuff tear	Pain (VAS) 12 mo	MD=-0.49 (-1.02-0.05)	Moderate
		Otolaryngology			
Surgical orbital decompression	IV Methylprednisolone 1x3 followed by oral prednisolone	Thyroid eye disease	Proportion of successes compared to the proportion of treatment failures as defined by the study authors based on the use of composite outcome scores	RR=0.16 (0.01-1.98)	
		Thoracic surgery			
Open thoracotomy	Thoracostomy drainage (with fibrinolytics)	Pleural empyema	Mortality	RR=NA (NA-NA)	Moderate
VATS	Thoracostomy drainage (with fibrinolytics)	Pleural empyema	Mortality	RR=0.8 (0.04-14.89)	Low
		Urology			
Surgical reimplantation of ureters	Antibiotics	Primary vesicoureteric reflux	Rate of patients with symptomatic UTI	RR=0.95 (0.67-1.35)	
		Vascular surgery			
Surgery including primary amputation	Thrombolysis (w/ rt-Pa or urokinase)	Acute limb ischaemia	Limb salvage (30 d)	OR=0.89 (0.27-2.91)	Low
Saphenofemoral disconnection	Therapeutic LMWH	Superficial thrombophlebitis	Symptomatic VTE	RR=5 (0.25-100)	
			Major bleeding	RR=NA	
Aspirin and carotid surgery	Aspirin	Carotid stenosis	Ipsilateral ischaemic stroke, and any operative stroke or death near occlusion	RR=0.89 (0.6-1.32)	Moderate

Abbreviations

RR: risk ratio

For peer review only - http://bmjopen.bmj.com/site/about/guidelines.xhtml

OR: odds ratio
HR: hazard ratio
MD: mean difference
SMD: standardized mean difference

BCC: basal cell carcinoma of the skin
GERD: Gastro-oesophageal reflux disease
GTN: glyceryl tri-nitrate
IOP: intra-ocular pressure
PCOS: polycystic ovarian syndrome
QOL: Quality of life

For peer review only

For peer review only - http://bmjopen.bmj.com/site/about/guidelines.xhtml

PRISMA 2020 Checklist

Section and Topic	Item #	Checklist item	Location where item is reported
**TITLE**			
Title	1	Identify the report as a systematic review.	1
**ABSTRACT**			
Abstract	2	See the PRISMA 2020 for Abstracts checklist.	4
**INTRODUCTION**			
Rationale	3	Describe the rationale for the review in the context of existing knowledge.	6
Objectives	4	Provide an explicit statement of the objective(s) or question(s) the review addresses.	4
**METHODS**			
Eligibility criteria	5	Specify the inclusion and exclusion criteria for the review and how studies were grouped for the syntheses.	7
Information sources	6	Specify all databases, registers, websites, organisations, reference lists and other sources searched or consulted to identify studies. Specify the date when each source was last searched or consulted.	7
Search strategy	7	Present the full search strategies for all databases, registers and websites, including any filters and limits used.	7
Selection process	8	Specify the methods used to decide whether a study met the inclusion criteria of the review, including how many reviewers screened each record and each report retrieved, whether they worked independently, and if applicable, details of automation tools used in the process.	7
Data collection process	9	Specify the methods used to collect data from reports, including how many reviewers collected data from each report, whether they worked independently, any processes for obtaining or confirming data from study investigators, and if applicable, details of automation tools used in the process.	8
Data items	10a	List and define all outcomes for which data were sought. Specify whether all results that were compatible with each outcome domain in each study were sought (e.g. for all measures, time points, analyses), and if not, the methods used to decide which results to collect.	8
	10b	List and define all other variables for which data were sought (e.g. participant and intervention characteristics, funding sources). Describe any assumptions made about any missing or unclear information.	8
Study risk of bias assessment	11	Specify the methods used to assess risk of bias in the included studies, including details of the tool(s) used, how many reviewers assessed each study and whether they worked independently, and if applicable, details of automation tools used in the process.	8
Effect measures	12	Specify for each outcome the effect measure(s) (e.g. risk ratio, mean difference) used in the synthesis or presentation of results.	8
Synthesis methods	13a	Describe the processes used to decide which studies were eligible for each synthesis (e.g. tabulating the study intervention characteristics and comparing against the planned groups for each synthesis (item #5)).	NA
	13b	Describe any methods required to prepare the data for presentation or synthesis, such as handling of missing summary statistics, or data conversions.	7-8
	13c	Describe any methods used to tabulate or visually display results of individual studies and syntheses.	7
	13d	Describe any methods used to synthesize results and provide a rationale for the choice(s). If meta-analysis was performed, describe the model(s), method(s) to identify the presence and extent of statistical heterogeneity, and software package(s) used.	8
	13e	Describe any methods used to explore possible causes of heterogeneity among study results (e.g. subgroup analysis, meta-regression).	Not relevant
	13f	Describe any sensitivity analyses conducted to assess robustness of the synthesized results.	Not relevant
Reporting bias assessment	14	Describe any methods used to assess risk of bias due to missing results in a synthesis (arising from reporting biases).	10
Certainty	15	Describe any methods used to assess certainty (or confidence) in the body of evidence for an outcome.	7-8

For peer review only - http://bmjopen.bmj.com/site/about/guidelines.xhtml

PRISMA 2020 Checklist

Section and Topic	Item #	Checklist item	Location where item is reported
assessment			
**RESULTS**			
Study selection	16a	Describe the results of the search and selection process, from the number of records identified in the search to the number of studies included in the review, ideally using a flow diagram.	11
	16b	Cite studies that might appear to meet the inclusion criteria, but which were excluded, and explain why they were excluded.	11
Study characteristics	17	Cite each included study and present its characteristics.	Supplement 1
Risk of bias in studies	18	Present assessments of risk of bias for each included study.	11 (GRADE)
Results of individual studies	19	For all outcomes, present, for each study: (a) summary statistics for each group (where appropriate) and (b) an effect estimate and its precision (e.g. confidence/credible interval), ideally using structured tables or plots.	12-13
Results of syntheses	20a	For each synthesis, briefly summarise the characteristics and risk of bias among contributing studies.	11
	20b	Present results of all statistical syntheses conducted. If meta-analysis was done, present for each the summary estimate and its precision (e.g. confidence/credible interval) and measures of statistical heterogeneity. If comparing groups, describe the direction of the effect.	p. 13 Table 2 & 3
	20c	Present results of all investigations of possible causes of heterogeneity among study results.	Not relevant
	20d	Present results of all sensitivity analyses conducted to assess the robustness of the synthesized results.	Not relevant
Reporting biases	21	Present assessments of risk of bias due to missing results (arising from reporting biases) for each synthesis assessed.	13
Certainty of evidence	22	Present assessments of certainty (or confidence) in the body of evidence for each outcome assessed.	13
**DISCUSSION**			
Discussion	23a	Provide a general interpretation of the results in the context of other evidence.	15
	23b	Discuss any limitations of the evidence included in the review.	16
	23c	Discuss any limitations of the review processes used.	16
	23d	Discuss implications of the results for practice, policy, and future research.	17
**OTHER INFORMATION**			
Registration and protocol	24a	Provide registration information for the review, including register name and registration number, or state that the review was not registered.	3
	24b	Indicate where the review protocol can be accessed, or state that a protocol was not prepared.	3
	24c	Describe and explain any amendments to information provided at registration or in the protocol.	9
Support	25	Describe sources of financial or non-financial support for the review, and the role of the funders or sponsors in the review.	2
Competing interests	26	Declare any competing interests of review authors.	2
Availability of data, code and other materials	27	Report which of the following are publicly available and where they can be found: template data collection forms; data extracted from included studies; data used for all analyses; analytic code; any other materials used in the review.	2

For peer review only

From: Page MJ, McKenzie JE, Bossuyt PM, Boutron I, Hoffmann TC, Mulrow CD, et al. The PRISMA 2020 statement: an updated guideline for reporting systematic reviews. BMJ 2021;372:n71. doi:

PRISMA 2020 Checklist

10.1136/bmj.n71

For more information, visit: http://www.prisma-statement.org/

For peer review only

PRISMA 2020 for Abstracts Checklist

Section and Topic	Item #	Checklist item	Reported (Yes/No)
**TITLE**			
Title	1	Identify the report as a systematic review.	Yes
**BACKGROUND**			
Objectives	2	Provide an explicit statement of the main objective(s) or question(s) the review addresses.	Yes
**METHODS**			
Eligibility criteria	3	Specify the inclusion and exclusion criteria for the review.	Yes
Information sources	4	Specify the information sources (e.g. databases, registers) used to identify studies and the date when each was last searched.	Yes
Risk of bias	5	Specify the methods used to assess risk of bias in the included studies.	Yes
Synthesis of results	6	Specify the methods used to present and synthesise results.	Yes
**RESULTS**			
Included studies	7	Give the total number of included studies and participants and summarise relevant characteristics of studies.	Yes
Synthesis of results	8	Present results for main outcomes, preferably indicating the number of included studies and participants for each. If meta-analysis was done, report the summary estimate and confidence/credible interval. If comparing groups, indicate the direction of the effect (i.e. which group is favoured).	Yes
**DISCUSSION**			
Limitations of evidence	9	Provide a brief summary of the limitations of the evidence included in the review (e.g. study risk of bias, inconsistency and imprecision).	Yes
Interpretation	10	Provide a general interpretation of the results and important implications.	Yes
**OTHER**			

For peer review only

Funding	11	Specify the primary source of funding for the review.	Yes
Registration	12	Provide the register name and registration number.	Yes

From: Page MJ, McKenzie JE, Bossuyt PM, Boutron I, Hoffmann TC, Mulrow CD, et al. The PRISMA 2020 statement: an updated guideline for reporting systematic reviews. BMJ 2021;372:n71. doi: 10.1136/bmj.n71

For more information, visit: http://www.prisma-statement.org/

For peer review only
